# Regional patterns of diachronic technological change in the Howiesons Poort of southern Africa

**DOI:** 10.1371/journal.pone.0239195

**Published:** 2020-09-17

**Authors:** Manuel Will, Nicholas J. Conard

**Affiliations:** 1 Department of Early Prehistory and Quaternary Ecology, University of Tübingen, Tübingen, Germany; 2 Senckenberg Center for Human Evolution and Paleoecology, University of Tübingen, Tübingen, Germany; Monash University, AUSTRALIA

## Abstract

The Howiesons Poort (HP) of southern Africa plays an important role in models on the early behavioral evolution of *Homo sapiens*. The HP is often portrayed as a coherent MSA industry characterized by early complex material culture. Recent work has emphasized parallel technological change through time across southern Africa potentially driven by ecological adaptations or demographic change. Here we examine patterns of diachronic variation within the HP and evaluate potential causal factors behind these changes. We test previous temporal assessments of the technocomplex at the local and regional level based on high-resolution quantitative data on HP lithic assemblages from Sibudu (KwaZulu-Natal) and comparisons with other southern African sites. At Sibudu, consistent unidirectional change in lithic technology characterizes the HP sequence. The results show a gradual reduction in typical HP markers such as the proportion of blades, backed pieces, and HP cores, as well as declining size of blades and backed artifacts. Quantitative comparisons with seven HP sites in South Africa suggest that lithic technology varies between regions over time instead of following similar changes. Concerning hypotheses of causal drivers, directional changes in lithic technology at Sibudu covary with shifting hunting patterns towards larger-sized bovids and a gradual opening of the vegetation. In contrast, variation in lithic technology shows little association with site use, mobility patterns or demographic expansions. Unlike at Sibudu, diachronic changes at other HP sites such as Diepkloof, Klasies River and Klipdrift appear to be associated with aspects of mobility, technological organization and site use. The regional diachronic patterns in the HP partly follow paleoclimatic zones, which could imply different ecological adaptations and distinct connection networks over time. Divergent and at times decoupled changes in lithic traits across sites precludes monocausal explanations for the entire HP, supporting more complex models for the observed technological trajectories.

## Introduction

The archaeological period of the Middle Stone Age (MSA) is confined to the African continent and dates to ~300–40 ka. The MSA has attracted much scholarly interest as it encompasses the biological origin [1, 2] and early cultural evolution of our species [3–6]. Current models for the cultural evolution of *Homo sapiens* differ in the timing of behavioral change, its causal factors, and geographical scope. Klein [3, 7–10] favors a sudden origin of modern behaviors within Africa at around ~50–40 ka. He explains this abrupt change by favorable neuronal genetic mutations that coincide with the beginnings of the Later Stone Age (LSA). In contrast, McBrearty and Brooks [4] propose a long, gradual and cumulative cultural evolution of modern humans within Africa that already began at ~200–150 ka. Taking a global scope, Conard [5, 11, 12] advances a model of “Mosaic Polycentric Modernity” which rejects the idea of a single source of cultural innovations in Africa. This scenario suggests multiple origins playing out on diverse timescales since the late Middle Pleistocene. Based on an updated review of the MSA record, Will *et al*. [13] proposed divergent trajectories for cultural innovations and complex behaviors among different parts of Africa during the Middle and Late Pleistocene, characterized by non-linear timelines.

Models on smaller spatial scales within Africa also exist, and recent discussions have focused on the rich South African MSA record. The region boasts numerous findings of early examples of complex material culture such as abstract engravings on ochre [14] and ostrich eggshell [15], organic tools [16], and shell beads [17], among other things [18 for an overview]. Many of these finds date to MIS 4 (~71–60 ka) and are associated with the Still Bay (SB) and Howiesons Poort (HP) technocomplexes. On this basis, Jacobs, Henshilwood and colleagues [19–22] developed a model that characterizes the behavioral evolution of *H*. *sapiens* in this region by abrupt and discontinuous cultural change, materialized by two short periods of exceptional cultural innovation. Other researchers have inferred a spread of these elements to the north and out of Africa, integrating them into larger-scale models for the evolution and dispersal of *Homo sapiens* [23–27]. This being said, more recent research within the southern African MSA has criticized this model of discontinuous evolution of cultural capacities on both empirical and theoretical grounds [28–35].

It is no coincidence that current models of cultural evolution center around the HP, the best-known MSA technocomplex in southern Africa. The HP occurs at >20 sites distributed over various ecological and geographical zones. Researchers define the HP by its characteristic lithic technology, including various geometric forms of backed tools, laminar technology and increased proportions of fine-grained and potentially non-local raw materials [36–43]. The period has yielded outstanding finds such as organic ornaments, bone tools, pigment use, abstract depictions, and microlithic technology [see 18, 21, 44]. Many studies have concluded that the HP is a distinctive cultural unit with regard to its lithic technology that can be used as a marker horizon [36–39, 44–50]. Chronologically, the HP generally falls within a relatively short time frame of ~66–59 ka [19, 20, 51]. However, ages of up to ~100 ka [52, 53] and as young as ~46–42 ka [54] complicate this assessment [55–57]. Researchers have explained the onset and disappearance of the HP have by various factors, including adaptations to ecological and environmental changes, including shifting territorial organization and mobility patterns [25, 58–61], changes in subsistence and hunting behavior [62, 63], increases in population size [20, 22, 64], or information transmission between closely connected groups [65].

Recent work in southern Africa has started to provide analyses of HP lithic technology on finer scale than the entire technocomplex. These studies have documented higher levels of intra- and inter-site techno-typological variability [32, 42, 66–72], and some indications for consistent diachronic trends within sites (see below). Such analyses have ramifications for the study of variability in the HP and the abandonment of its specific lithic technology. Our research focus here lies on the patterns and causes of temporal change during the HP on intra- and inter-site scales. We test previous hypotheses using new lithic data on diachronic changes in the rich and high-resolution HP sequence from Sibudu, one of the key MSA sites. We then compare the diachronic pattern from Sibudu in a quantitative manner to HP sequences from the rest of the subcontinent. These results help us to establish the pattern of behavioral variation during the HP across southern Africa and evaluate potential causes of these changes.

## Research questions and expectations

Our study examines three main research questions through a quantitative analysis of lithic assemblages from the HP at Sibudu and comparisons with other southern African localities.

1. What is the nature of temporal change in lithic technology during the HP at Sibudu?
2. Is the pattern of diachronic change uniform across HP sequences in southern Africa?
3. What are the causal factors driving technological changes in the HP over time?

Regarding the first question, detailed work on the HP lithic assemblages at Sibudu from Lyn Wadley’s excavation has produced manifold, high-quality data on tool assemblages, core reduction and functional interpretation [67–70, 73–78]. These studies emphasized detailed qualitative analyses of individual assemblages, with little emphasis on examining diachronic trends within the HP sequence. The amount of stratigraphic variability and the direction of temporal changes in the high-resolution HP sequence of Sibudu remain presently open questions in need of additional research.

Recent assessment of other HP sites with long stratigraphic sequences such as Diepkloof, Klipdrift, Rose Cottage and Umhlatuzana have produced a picture of consistent diachronic variability with two or three phases or “developmental stages” within the HP [40, 42, 79–82]. At the longest and well-studied HP sequence of Diepkloof, the excavators distinguish an Early, Intermediate and Late phase. Porraz *et al*. [42: 3395] further propose that at most southern African sites, the industries probably correspond to the Intermediate and Late phase (“classic HP”) from Diepkloof. Other researchers have also suggested that similar typological and technological trends characterize the HP across sites [40, 49, 71, 82, 83]. If correct, this would be evidence for parallel behavioral trajectories across the subcontinent, several thousands of years and multiple biomes with important implications for communication networks and patterns of cultural evolution. Our comparative analyses of new data from Sibudu with seven HP sequences aims to test this hypothesis of a parallel and uniform pattern of temporal changes in HP lithic technology and typology across southern African sites.

Regarding our third question, we are interested in the causal factors behind technological variability in the HP of Sibudu and South Africa. Many potential and often conflicting factors driving cultural changes have been proposed for the HP and the MSA more generally. Table 1 provides an overview of potential archaeological correlates and expectations of these causal hypotheses. Sibudu with its rich, high-resolution stratigraphy and organic preservation allows us to examine some of these hypotheses in detail [see e.g. 84], and we extend this on a coarser scale to other HP sites in as much as the published data permit.

**Table 1:** Proposed causal factors for changes in southern African MSA lithic technology, their archaeological correlates and expectations.

Causal factor	Archaeological correlate	Expectation
Ecological adaptations	Paleoenvironmental reconstructions (faunal/botanical remains); Rainfall zones	Similar ecologies yield similar technological signals; different technological signals in different ecologies
Territoriality and mobility patterns	Raw material procurement distances; Lithic technology	In/decrease in non-local rocks; more/less formalized technologies
Demographic expansion	Lithic density; faunal density; occupation intensity (e.g. geoarchaeology); site number	Increase in lithic and faunal densities; Higher frequency of sites; higher anthropogenic signal in sediments
Subsistence behavior	Faunal remains; Lithic technology (e.g. tool types)	Change in lithic technology (e.g. tool types) correlates with prey changes
Information networks	Lithic technology (e.g. learned behaviors; lithic domains; attribute data)	Higher similarity in multiple lithic domains across like-aged and geographically closer sites

## Materials and methods

### The site of Sibudu: Overview and excavation methods

**Fig 1:**
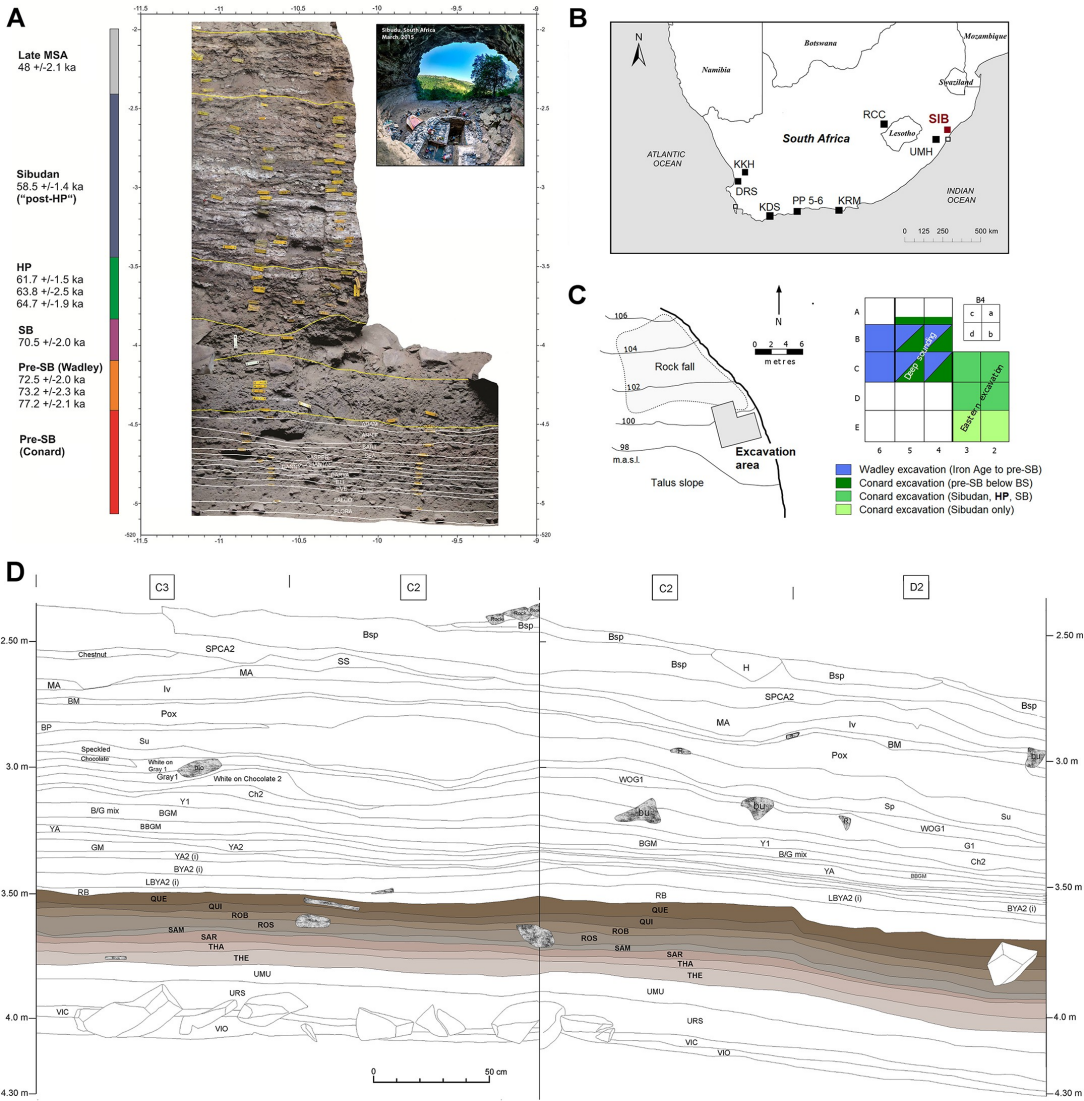
The site of Sibudu. A) Composite stratigraphy with indication of chronometric ages and cultural attributions (2D georectified photomosaic created with Agisoft Metashape of photos taken by Mohsen Zeidi in 2018 with a Nikon Coolpix P510 digital camera in daylight, not color-corrected); B) Geographical map of Sibudu (SIB) and comparative HP sites. C) Horizontal plan of the rockshelter floor and excavation grid, with indication of the Wadley and Conard excavations in different vertical levels. D). Stratigraphic section of the Eastern Excavation (combined north and east profile) of Sibudu with all HP layers (THE-QUE) excavated during 2016–2018 indicated in color. (Figure by V.C. Schmid, M. Zeidi, M. Haaland, A. Val and M. Will).

Sibudu is a rockshelter overlooking the uThongathi River in KwaZulu-Natal, South Africa, located about 40 km north of Durban (Fig 1). Lyn Wadley directed 25 field seasons at Sibudu from 1998–2011. On Wadley’s invitation, the archaeological work at Sibudu has been continued by a team of the University of Tübingen under N. Conard’s direction since 2011. Wadley’s team excavated the MSA deposits of the site over an area of 21 m^2^ to up to three meters. This sequence dates between <77–38 ka and includes Wadley’s pre-SB, SB, HP, “post-HP”, late MSA, and final MSA strata [85–88]. The new field work focused on re-excavating the upper portion of the site, beginning at the top of the “post-HP”, and has now recovered the full HP sequence. All necessary permits were obtained for the described study, which complied with all relevant regulations. The research permit to conduct archaeological excavations at Sibudu is issued under the KwaZulu-Natal heritage Act No. 4 by Amafa AkwaZulu-Natali (permit number: REF:0011/14; object 2031CA 070). All recovered archaeological specimens (including those of this study) are housed in the KwaZulu-Natal Museum in Pietermaritzburg, 237 Jabu Ndlovu Street. The specimen numbers of this study are C2.2377–4332; C3.2204–4629; D2.1829–4111; D3.2189–4484 (including sub-numbers).

Sibudu features excellent organic preservation and high densities of archaeological remains in its thick and high-resolution sequence. We adapted our excavation methods to the rich archaeological materials and complex stratigraphy. We excavated in 1–3 cm thick *Abträge* (singular *Abtrag*) to establish high-resolution cultural chronological units that follow the contours of the stratigraphic sequence. Unlike spits, *Abträge* follow the natural slope of the sediments and do not crosscut geological strata. We group these *Abträge* in larger stratigraphic units that we call find horizons (i.e. layers), with individual HP strata consisting of 2–5 *Abträge* (~4–10 cm). The find horizons constitute our main analytical units. All sediments have been excavated with careful piece-plotting of artifacts >3 cm, using a Leica total station. We collected smaller material by screening buckets of sediment, which are subsequently sorted and catalogued. While we aimed for maximal comparison with the previous profile [e.g. 31, 34], we were not able to reproduce stratigraphic findings from Wadley’s fieldwork in this part of the sequence. This difficulty is associated with the lack of clear stratigraphic markers, the sloping of sediments (Fig 1), lateral facies shifts and different field methods. In order to emphasize this difference, we followed a new naming system based on the names of people in alphabetical order, similar to those used by colleagues on the Western Cape [42]. While this approach complicates direct comparisons to the Wadley excavations, it does reflect the reality on the ground instead of pressing our results in a stratigraphic scheme that does not fit.

### Stratigraphic sequence and lithic assemblages from the HP

The HP at Sibudu lies directly below the Sibudan [34] and on top of the SB. We excavated this part on 4 m^2^ and a total volume of 1.23 m^3^. The depositional sequence of ~30–50 cm thickness includes a succession of 8 find horizons (in 25 *Abträge*) which are almost entirely of anthropogenic origin, without any major disturbances or obvious post-depositional movement of finds [89, 90]. From bottom to top these are layers Theodora, Thabo, Sarah, Saman, Rosa, Robert, Quincy and Quentin (Fig 1). Densities of lithic and other remains are extraordinarily high in this part of the sequence, attesting to multiple intense and repeated occupations during the HP (S1 Table in S1 File). The sequence has previously been dated to between ~65–60 ka by OSL [19, 57].

The total material from the Conard HP excavations encompasses stone artifacts (n = 97,360), faunal remains (~60 kg), ochre (n = 422), as well as charcoal, botanical remains and organic tools. All finds >3 cm are labelled and stored in the database with unique find numbers, allowing individual piece identification and precise 3D spatial location. The HP assemblages vary in sample size, but not drastically (Table 2). Debitage <30 mm was sampled for raw material and used in density calculations. Lithic densities are very high (~51,000–124,000 n/m^3^) for an MSA site and similar to or higher than the overlying Sibudan at Sibudu (S1 Table in S1 File).

**Table 2:** Distribution of debitage categories (≥30 mm) and small debitage (<30 cm) in each layer of the Sibudu HP.

Layer	Blanks	Tools	Core	Angular debris	Total >30 mm	Small debitage <30mm
QUENTIN	930	39	18	38	1025	13986
QUINCY	416	26	7	9	458	6799
ROBERT	655	61	16	21	753	11291
ROSA	717	60	11	14	802	13327
SAMAN	591	40	12	11	654	8684
SARAH	392	36	6	12	446	5225
THABO	1315	103	23	38	1479	14265
THEODORA	1990	129	43	52	2214	15952
Total	7006	494	136	195	7831	89529

### Methods of lithic analysis

We followed an attribute analysis approach for all lithics >30 mm (n = 7831) that focuses on the recording of comparative quantitative data and subsequent statistical analyses. Attribute analysis informs on technological behaviors by providing data of the numerous traces on individual artifacts that result from the knapping process [e.g. 91–93]. Recently, MSA studies in southern Africa have strived to produce more comparable technological analyses based on quantitative attribute data, a crucial step towards more standardized lithic studies [29, 31, 47, 81, 94–96]. Our database consisted of ~40 discrete and metric attributes on blanks, tools, cores, based on MSA lithic studies and relevant to our main questions (S1 Text in S3 File and S1 Dataset). All recorded attributes are entered into an Access Database, allowing for subsequent statistical analyses on the assemblage level or selected samples.

The large size cut-off (>30 mm) has resulted in an overall reduced proportion of small bladelets compared to other HP sites using a cut-off >20 mm. The proportions presented for these blank types here should thus be seen as an underestimation. Since the size cut-off was applied to all HP layers, this methodical choice does not affect the diachronic comparability of blank types among our excavations. It did, however, lead to differences in assessment compared to previous studies at Sibudu with regard to the proportion of microlithic strategies [see for more details: 67, 69, 70, 73]. This being said, retouched artefacts and cores have been analyzed regardless of size, including n = 129 backed pieces and n = 50 cores with maximum dimension <30 mm and occurring in all HP layers. The following presentation of data will focus on diachronic trends within the sequence instead of detailed descriptions of individual assemblages or the HP as a whole. Yet, we test for diachronic trends among all major domains of lithic technology [*sensu* 97] in order to characterize change along multiple axes of stone knapping behavior. More detailed assessments of the technology and *chaîne opératoires* of the HP can be found in previous work on the Wadley material (see above). Our results did not provide contradictory information to these findings unless otherwise stated, but adds more quantitative data in a diachronic framework.

### Methods of statistical analysis

To test for temporal variation in the HP sequence at Sibudu, we employ multiple levels of statistical analyses. While many of these statistical procedures have been applied to southern African MSA assemblages [e.g. 47, 48, 95], specific tests for assessing diachronic trends have not been performed for any HP sequence so far. We calculated relative proportions (in %), means and coefficients of variation (CV) [see 98] to assess central tendencies and dispersion. Some of these parameters are also reported for other HP sequences, allowing for direct comparison. We performed univariate analyses on various lithic attributes (e.g. raw material frequency) in association with time (i.e. stratigraphic layer) to test for the statistical significance of potential diachronic trends. We chose non-parametric Kendall rank correlation coefficient (Kendall’s tau; τ) as the most appropriate test, since it can process both continuous and discrete ordinal variables, is robust against outliers, performs well with small sample sizes (e.g. here for layers n = 8) and works on data without normal distribution [99]. Compared to Spearman’s rho, Kendalls tau exhibits a smaller gross error-sensitivity, making it a more robust method (i.e. less sensitive towards outliers) that also works better with small sample sizes [see 99]. These tests assess linear trends (i.e. consistent increase or decrease), with the identification of other chronological patterns, such as quadratic or cubic, requiring parametric tests and n>30. Such data are unavailable for Sibudu or any of the other studied HP sites. In addition, we used parametric (t-test, ANOVA) and non-parametric (Chi-Square; Mann-Whitney U; Kruskal-Wallis) methods depending on data distribution and sample size [100–103] to test for significant differences in distributions of categorical data and means of metric variables between assemblages. We employ *k*-means clustering and Principal Component Analyses (PCA) in order to examine and illustrate diachronic patterns in the HP sequence at Sibudu taking into account multiple lithic variables at once. *k*-means clustering [104] partitions the number of observations into a lower number of *k* clusters to minimize the within-cluster variance of the multivariate dataset used, with each observation (layer) attributed to a cluster with the nearest mean. PCA reduces the number of dimensions of multivariate datasets and summarizes the main aspects of variability in the given sample by calculating uncorrelated indices that reflect combinations of variables [105] (for an example on lithic attribute data, see [106]). Prior to the PCA analyses, variables were normalized by Box-Cox Transformations.

## Results

### Raw material procurement and use

**Fig 2:**
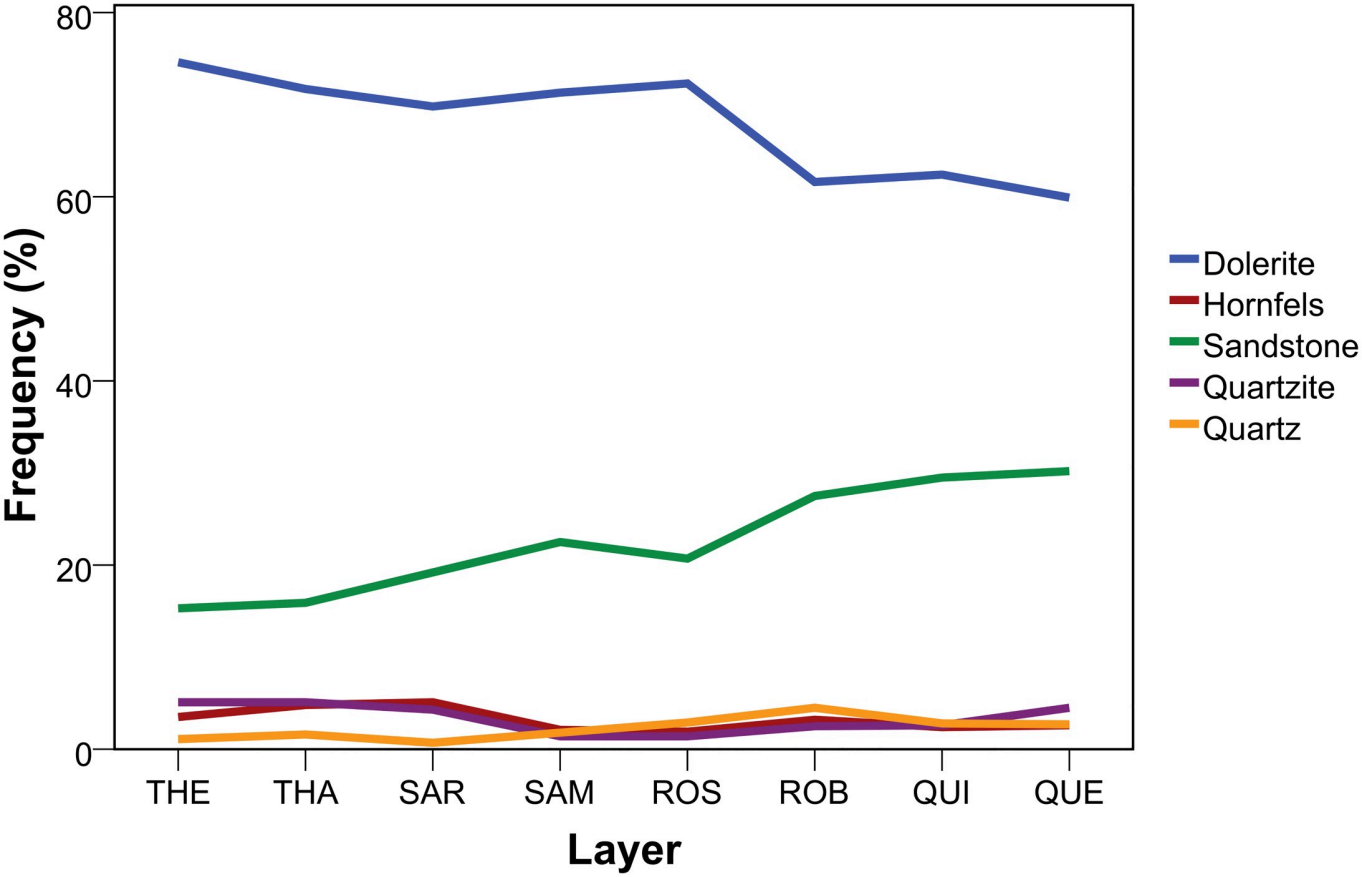
Diachronic variation of raw materials throughout the HP at Sibudu. THE = oldest layer; QUE = youngest layer.

Knappers during the HP at Sibudu principally used the same rock types as during other occupation phases: dolerite, hornfels, sandstone, quartzite, quartz, as well as small amounts of CCS, crystal quartz and jasper (subsumed under “other”). Except for hornfels and the other category, all raw materials can be locally procured (<10 km). The HP at Sibudu shows a dominance of dolerite (69%) followed by sandstone (21%) with all other raw materials <4%. Tabulating raw materials per layer shows a consistent diachronic trend throughout the sequence, particularly with regard to the two most frequently procured dolerite and sandstone (Fig 2; Table 3). Dolerite reaches a peak in the lowermost assemblages (THEODORA-THABO; 75–72%) followed by a slight decline in the middle part of the sequence (SARAH-ROSA; 72–70%), and the lowest value for the uppermost layers (ROBERT-QUENTIN; 62–60%). Sandstone shows a diametrically opposed pattern with a steady increase throughout the sequence and the lowest values at the bottom (15–16%) compared to the uppermost assemblages (QUINCY-QUENTIN: ~30%). The diachronic trends in both raw materials follow a significant monotonic relationship (dolerite: τ_b_ = -0.643, p = 0.026; n = 8; sandstone: τ_b_ = 0.929, p = 0.001; n = 8) with the variability between these raw materials being highly correlated (τ_b_ = -0.714, p = 0.013; n = 8). The gradual and significant nature of this change is further supported by a Chi-Square test (χ2 (7, n = 7058) = 158.5, p<0.001; ϕc = 0.150) and the distribution of the adjusted residuals (S2 Table in S1 File).

**Table 3:** Raw material distribution for lithic debitage >30 mm (n) by layer for the Sibudu HP.

Layer	Dolerite	Hornfels	Sandstone	Quartzite	Quartz	CCS/Other	Total
QUENTIN	614	27	310	46	28	0	1025
QUINCY	286	11	135	12	13	1	458
ROBERT	464	24	207	19	34	5	753
ROSA	580	15	166	11	23	7	802
SAMAN	466	14	147	9	12	6	654
SARAH	311	23	86	19	3	4	446
THABO	1060	71	236	75	24	13	1479
THEODORA	1651	77	339	112	25	10	2214
Total	5432	262	1626	303	162	46	7831

In terms of differential use, cortical cover is similar for all raw materials and by archaeological layer, having no cortex on the vast majority of pieces (72–83%), with highly cortical pieces (>60%) fluctuating between 3–6%. Sandstone was predominantly used for producing flakes (89%) but not blades and bladelets (~10%), whereas hornfels (59%) and other raw materials (54%) show high values for blades and bladelets. There is some variation for blade manufacture by raw material: Dolerite was most frequently used in the middle and upper part of the HP, sandstone particularly in the uppermost levels (QUENTIN-QUINCY; 12–13%) and quartzite only plays a role for blades in THEODORA-SARAH (5–7%). Tools in all assemblages of the HP were preferentially produced on hornfels, quartz and other raw materials, with quartz demonstrating overabundant cores (S3-S4 Tables in S1 File). The upper part of the sequence shows a stronger preference for quartz for retouching (ROBERT-QUINCY; 15–23%; mostly biface manufacture) compared to the lower and middle section (THEODORA-SAMAN; 1–8%). In sum, whereas the procurement of different raw materials shows consistent temporal trends, the approach to knapping for each rock type is comparatively stable throughout the sequence albeit with some diachronic change between the lowermost and uppermost assemblages.

### Blank production

**Fig 3:**
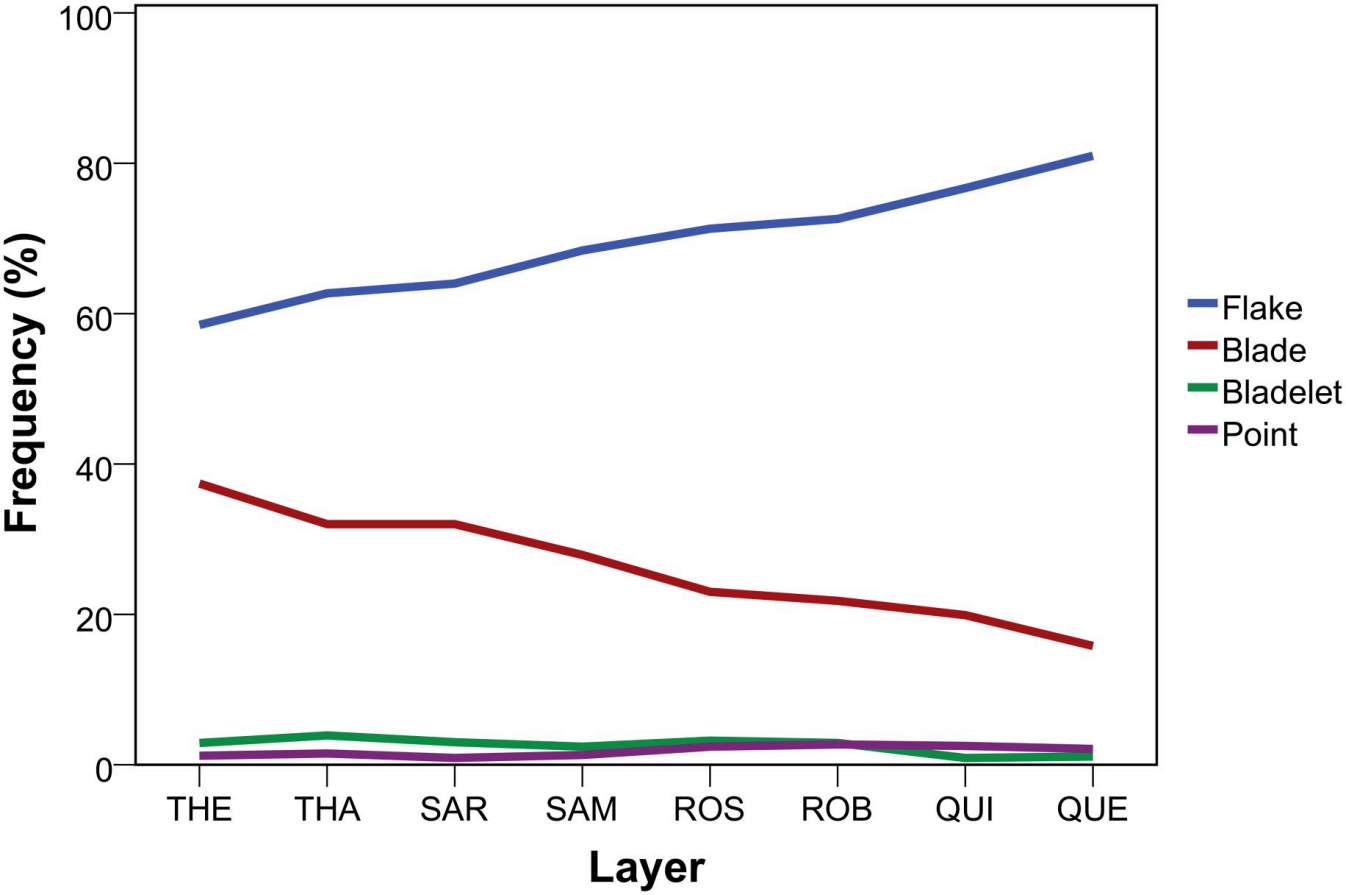
Diachronic variation of blank types produced throughout the HP at Sibudu. THE = oldest layer; QUE = youngest layer.

The overall frequency of unretouched blanks among all lithic find categories within the HP sequence is very similar (Table 2). Yet, the relative abundance of flakes and blades in particular varies strongly throughout the stratigraphic column (Fig 3). As expected for the HP, blades dominate the sample of blanks >3 cm with a total of 28.5% (n = 2133). The abundance by layer reveals an underlying temporal trend with blades showing a continuous decrease from the bottom (THEODORA; 37.4%) to the top (QUENTIN; 15.8%) of the HP (Table 4) that fits a highly significant negative monotonic relationship (τ_b_ = -0.982, p = 0.001; n = 8). Conversely, flake proportions continuously increase from the bottom (THEODORA; 58.5%) to the top (QUENTIN; 81%). A combined test of blade and flake proportions support this significant association (χ2 (7, n = 7163) = 218.5, p<0.001; ϕc = 0.175) and the consistent nature of the change is reflected in the adjusted residuals (S5 Table in S1 File). On an even finer level of stratigraphic resolution of the 25 consecutive *Abträge of* the HP, we find principally the same picture (τ_b_ = -0.778, p = <0.001; n = 25; S1 Fig in S2 File; S6 Table in S1 File), with the highest proportion of blades from *Abtrag* 2 in THEODRA (39.6%; n = 150) and the lowest abundance in *Abtrag* 2 of QUENTIN (11.9%; n = 52). The decrease of blades is not associated with blank sample size, but there is a negative correlation with increased sandstone use (τ_b_ = -0.929, p = 0.002; n = 8).

**Table 4:** Distribution of blank types for lithic debitage >30 mm (n) by layer for the Sibudu HP.

Layer	Flake	Blade	Bladelet	Point	TOTAL
QUENTIN	785	153	11	20	969
QUINCY	339	88	4	11	442
ROBERT	520	156	21	19	716
ROSA	555	179	25	19	778
SAMAN	431	176	15	8	630
SARAH	274	137	13	4	428
THABO	890	454	55	21	1420
THEODORA	1236	790	62	26	2114
Total	5030	2133	206	128	7497

The types of blanks used for retouching also vary. Blades (56.6% vs. 27.6%) and bladelets (14.5% vs. 2.9%) are much more frequent in the tool assemblages compared to their overall representation among all blanks, with flakes (67.7% vs. 25.8%) showing the opposite trend. Diachronically, blades are preferentially selected for retouch at the base of the HP (THEODORA-THABO: 12–19% flakes; 68–70% blades) whereas flakes are equally frequently used for secondary modification at the top (ROBERT-QUENTIN: 38–46% flakes; 38–42% blades).

**Fig 4:**
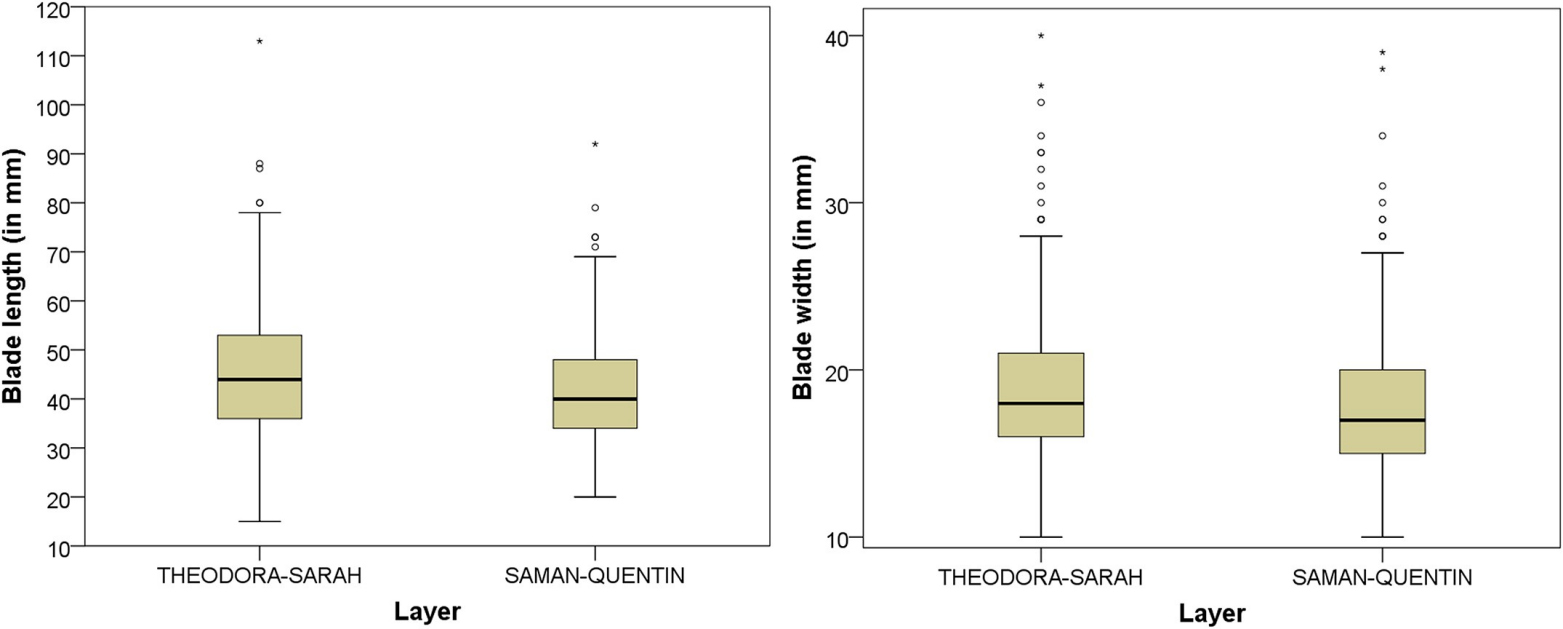
Box plot for length and width of blades between layers THEODORA-SARAH and SAMAN-QUENTIN.

Building on work from replication studies [107, 108] and previous analyses of MSA assemblages [e.g. 81], we examined diachronic trends in blade production by their metric attributes (see S1 Dataset for all measurements of blades and bladelets) and assessment of knapping techniques. In terms of dimensions, two diachronic trends are apparent: A stronger reduction in blade length coupled with a minor reduction in blade width (Table 5; S7 Table in S1 File). Length and width are on average largest in the lowermost assemblages THEODORA and SARAH (Length = 46.2–46.4 mm; Width 18.6–18.5 mm), followed by lower values throughout SAMAN-QUENTIN (Length = 44.5–40.5 mm; Width 17.3–18.1 mm). A t-test confirms the presence of significant differences between these two samples (length: t = 2.985; df = 550; p = 0.003; width: t = 4.837; df = 1434; p<0.001; Fig 4). A more detailed ANOVA and post-hoc analyses on the large sample of blade widths (F(3,1961) = 6.373; p<0.001) suggests that the most marked and significant changes exist between the bottom (THEODORA-THABO) vs. topmost layers (QUENTIN-QUINCY; S7 Table in S1 File & Fig 2). The analyses find no diachronic trend in blade thickness. Regarding metric standardization of blades, there is no consistent change, with overall CVs for length, width and thickness lowest at the top (QUENTIN; CVs = 21–32%) but also in the lower part (SARAH; CVs = 19–23%). Knapping technique of blades is a second axis of variation. Six discrete and metric variables yield a diachronic signal between the lower and middle (THEODORA-ROSA) versus upper part (ROBERT-QUINCY) of the sequence (Table 6). The lower and middle vs. upper layers feature less developed bulbs which are more frequently absent (46–60% vs. 34–50%), fewer shattered bulbs (3–6% vs. 7–13%), more lipping (22–31% vs. 11–17%), a lower mean EPA (80.2–81.2° vs. 82.7–83.7°) and slightly thinner platforms on average (3.5–4.1 mm vs. 3.8–4.2 mm). Multivariate *k*-means cluster analyses and PCA with these attributes confirm this patterning through time (S3 Fig in S2 File). The treatment of platforms remains stable, with faceting (18–30%) and abrasion (1–7%) varying in a non-directional manner (Table 6).

**Table 5:** Distribution of blade length and width (in mm) by layer for the Sibudu HP.

	Length				Width			
Layer	n	Mean	Max	CV (%)	n	Mean	Max	CV (%)
QUENTIN	46	41.2	69	23.1	135	17.5	34	21.1
QUINCY	31	44.5	79	30.8	81	17.5	29	21.7
ROBERT	41	42.4	71	25.2	144	18.1	39	25.4
ROSA	42	43.2	92	29.9	163	17.3	30	22.0
SAMAN	41	40.5	73	28.1	159	17.5	28	19.4
SARAH	40	46.4	68	22.8	124	18.5	29	19.1
THABO	93	43.3	113	32.4	423	18.4	44	22.8
THEODORA	218	46.2	88	27.1	744	18.6	37	21.7
TOTAL	552	43.3	113		1973	18.2	44	

**Table 6:** Attributes assessed to reconstruct knapping technique and platform treatment for complete and proximal blades (in % until otherwise noted) by layer for the Sibudu HP.

Layer	Developed bulb	Absent bulb	Shattered bulb	Lip	EPA (in °)	Facetted platform	Abraded platform	Platform thickness (mean, mm)	TOTAL (n)
QUENTIN	12.2	34.1	13.4	11.0	82.7	17.1	4.9	4.2	82
QUINCY	12.1	50.0	6.9	17.2	83.7	24.1	6.9	3.8	58
ROBERT	13.0	49.4	13.0	14.3	83.8	22.1	1.3	4.2	77
ROSA	8.6	45.7	6.2	23.5	81.2	29.6	4.9	3.9	81
SAMAN	10.6	51.1	3.2	22.3	81.2	21.3	3.2	3.6	94
SARAH	6.4	60.3	5.1	21.8	81.2	24.4	6.4	4.1	78
THABO	7.7	52.7	6.4	31.4	80.7	22.3	7.3	3.8	220
THEODORA	6.6	59.3	4.5	30.3	80.2	18.2	5.0	3.5	423
TOTAL	8.4	53.4	6.3	25.5	81.1	22.9	5.7	3.7	1113

### Core reduction

**Fig 5:**
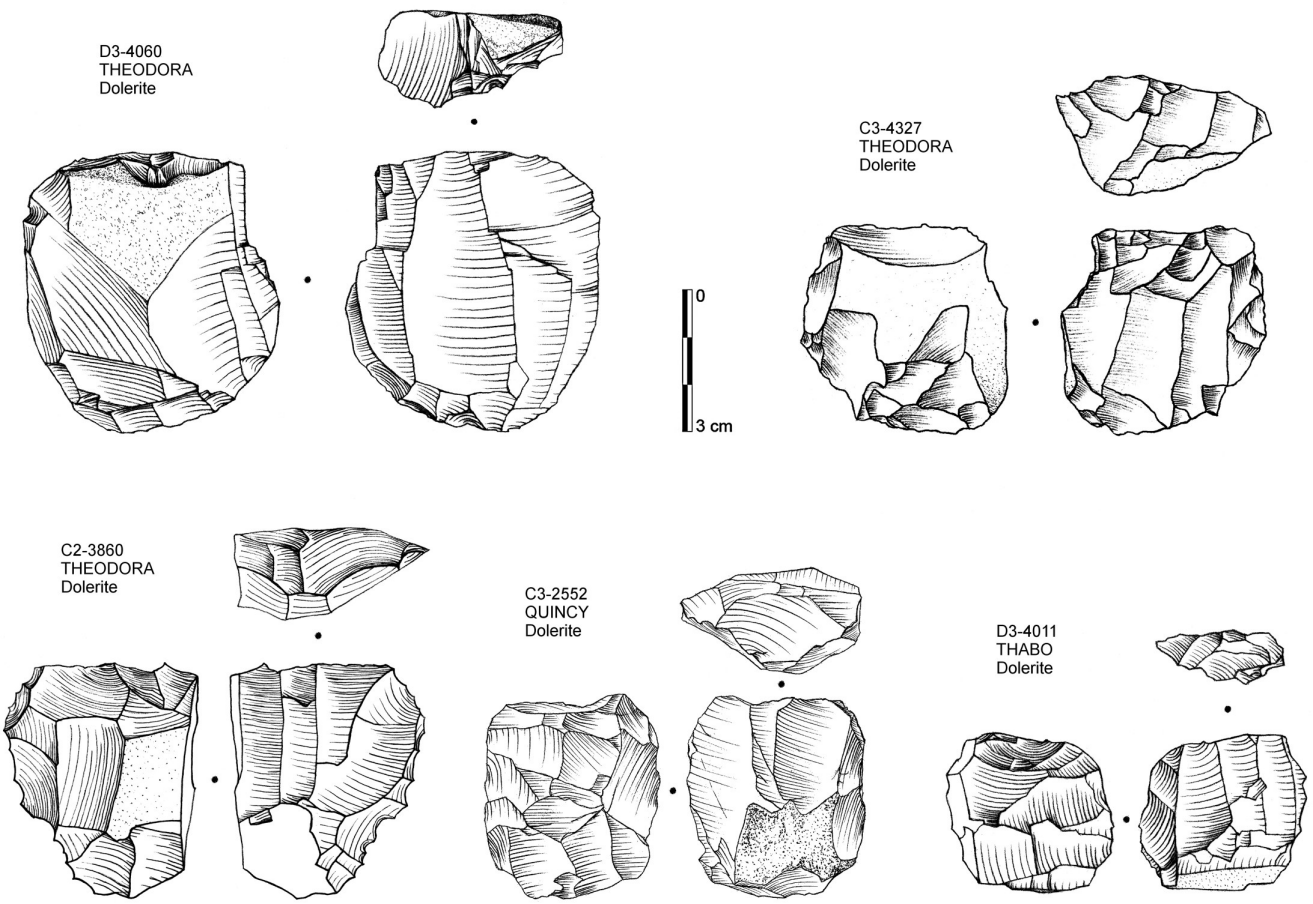
Drawings of HP cores from Sibudu with indication of find number, raw material and layer.

We classified cores according to the taxonomy developed by Conard *et al*. [109] (2004) for Africa providing a broad comparable framework for MSA studies. Among all cores from the HP at Sibudu, the vast majority can be classified as platform (44%; n = 58) and bipolar (41%; n = 55) reduction systems (S8 Table in S1 File). Parallel cores conforming largely to a Levallois system are uncommon (10%; n = 13) with only one potential inclined core in the sequence (1%). In terms of the products, blade (n = 39) and bladelet cores (n = 34) make up the majority (55%), with the remainder bearing mostly flake negatives. Dolerite was the preferred raw material for platform and parallel cores, whereas quartz and quartzite were frequently reduced in a bipolar manner (S4 Table in S1 File). Platform cores constitute the only reduction system performed on all raw materials. Among its platform cores, the HP at Sibudu features typical HP blade cores (Fig 5; *sensu* Villa et al. 2010) which have so far been exclusively found in this technocomplex in the southern African MSA. While HP cores occur in all analyzed layers, their relative abundance is much higher in the lower (THEODORA-THABO; 31–43%) and middle part of the sequence (SARAH-ROSA; 17–40%) with a strong decline in the uppermost assemblages (ROBERT-QUENTIN; 6–14%). Yet, this decrease in HP blade cores provides only a near-significant negative relationship (τ_b_ = -0.546, p = 0.061; n = 8). No comparable trends are found in parallel and bipolar cores and their products. Cores tend to be heavier, larger and thicker on average in the lowermost layers (THEODORA-THABO; 17.7–42.5 g, length: 29.6–34.8 mm; thickness: 15.2–19.6 mm) compared to the upper sequence (ROBERT-QUENTIN; 5.6–24.7 g; length: 20.8–29.5 mm; thickness 11.6–17 mm), yet this variation does not conform to a simple unidirectional decrease from bottom to top and is prone to extreme values within small sample sizes.

### Tool manufacture

**Fig 6:**
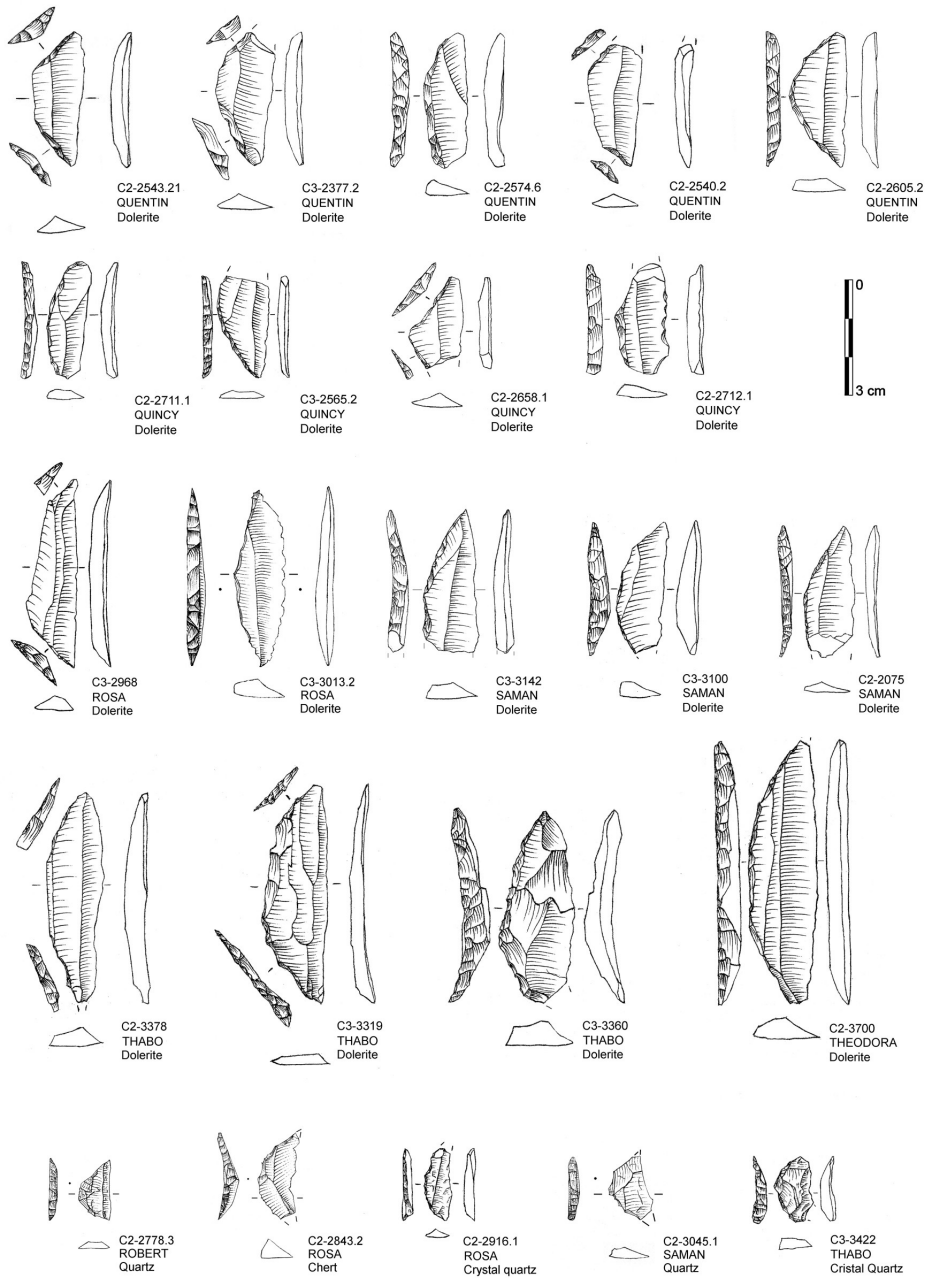
Drawings of various forms of backed pieces from Sibudu with indication of find number, raw material and layer.

**Fig 7:**
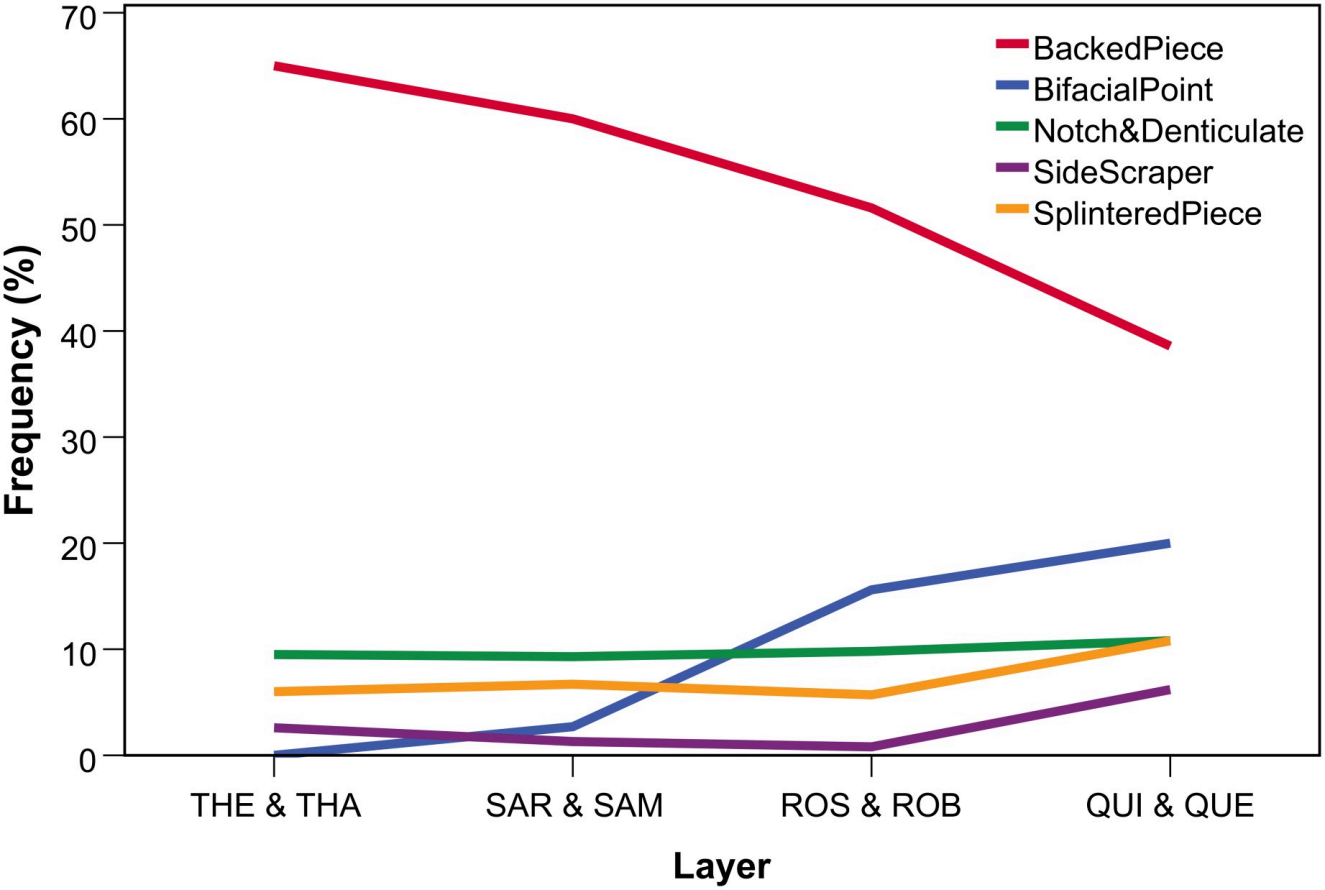
Diachronic variation of classic tool types throughout the HP at Sibudu.

Backed pieces including various distinct morphological sub-types such as segments and trapezoid constitute the most frequent tool type overall (57.3%) and in each layer with a total of n = 283 specimens recovered (Table 7; Fig 6). Other tool types are much less abundant, including notches and denticulates (n = 48; 9.7%), bifacial points (n = 34; 6.9%), splintered pieces (n = 33; 6.7%), lateral retouch (n = 25; 5.1%), scrapers (n = 12; 2.4%), unifacial points (n = 10; 2.0%) and rare strangulated pieces (n = 3; 0.6%). There is marked diachronic variation among the distribution of various retouched forms (Fig 7). For backed pieces, there is a continuous decrease from the bottom to top, with QUINCY and QUENTIN (64.7%), ROSA and ROBERT (60.0%), SARAH and SAMAN (51.6%), THEODORA and THABO (38.5%). A closer look at individual assemblages (Table 7) and by Abträge (S9 Table in S1 File; S4 Fig in S2 File) illustrates that this trend is not monotonous, but the Kendall’s tau-b correlation supports the overall linear trend from bottom to top by *Abträge* (τ_b_ = 0.356, p = 0.013; n = 25). A combined test of backed and bifacial piece proportions by layers supports this significant association (χ2 (3, n = 317) = 44.2, <0.001; ϕc = 0.417) with the biggest in-sequence change for both associated with QUINCY and QUENTIN (see S10 Table in S1 File). This fits the observation that densities of backed pieces have a minimum of 58–120 n/m^3^ in QUENTIN and QUENTY and frequencies <25% of backed pieces occur only in *Abtrag* 1–2 of QUENTIN and the lowermost *Abtrag* of QUINCY. The morphologies of backed pieces are relatively stable with trapezoids fluctuating only between 26–39% without clear diachronic trends, and segments at 46–63% with the uppermost layers reaching the highest values (QUENTIN and QUINCY; 59–63%).

**Table 7:** Frequency of tool types (n) for each lithic assemblage and the entire Sibudu HP.

Layer	Backed piece	Notch. & Dent.	Bifacial point	Unifacial point	Side scraper	Lateral retouch	Splintered piece	Strangulated piece	Minimal retouch
QUENTIN	17	2	10	1	3	1	5	0	0
QUINCY	8	5	3	2	1	3	2	0	2
ROBERT	27	4	14	2	1	4	3	0	6
ROSA	36	8	5	0	0	2	4	1	5
SAMAN	32	3	1	0	1	1	1	0	1
SARAH	13	4	1	1	0	4	4	0	8
THABO	72	9	0	4	1	3	6	1	6
THEODORA	78	13	0	0	5	7	8	1	15
Total	283	48	34	10	12	25	33	3	43

**Fig 8:**
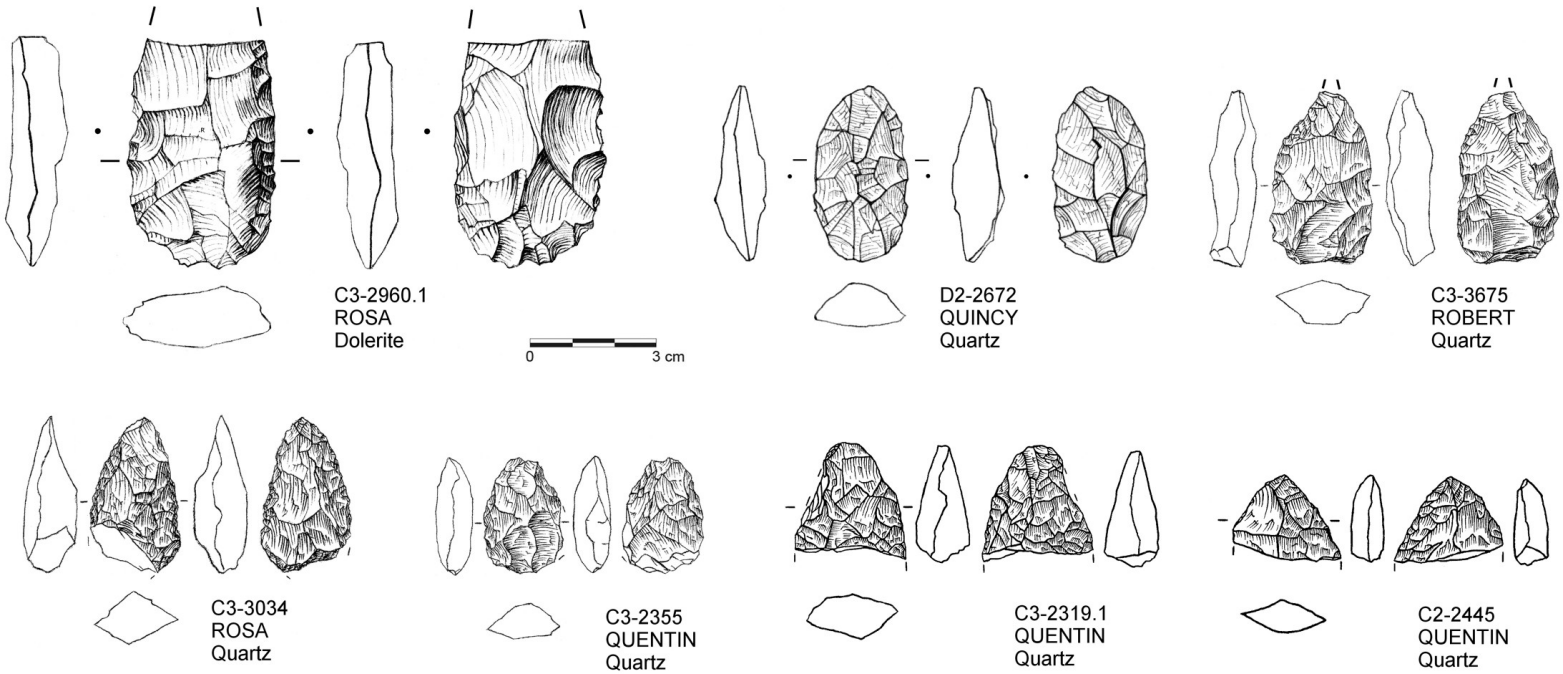
Drawings of bifacial pieces from Sibudu with indication of find number, raw material and layer. THE = oldest layer; QUE = youngest layer.

Bifacial points (Fig 8) constitute the other major tool class with marked diachronic trends, with notched and denticulated pieces being relatively stable throughout the sequence (Fig 7). Bifacial points are absent at the base of the HP (THEODORA and THABO), occur rarely in the middle (SARAH-ROSA; 2.5–8.2%; 25.8 n/m^3^), but frequently at the top (ROBERT-QUENTIN; 22.5–25.6%; 57.6 n/m^3^). A more detailed description of such pieces at Sibudu can be found elsewhere [e.g. 67]. Yet, the specimens from the Conard excavations fit these descriptions in being predominantly manufactured on quartz (n = 24; 70.6%), shaped in an asymmetric manner rarely resulting in facial or lateral symmetries and an overall much smaller mean size (length = 35.5 mm; width = 22.3 mm; thickness = 10.1 mm; total n = 34) compared to bifacial points from the Still Bay further below in the stratigraphy.

At Sibudu, all HP assemblages are characterized by high absolute tool type diversity (>7). Yet, when adjusting for tool sample size, relative diversity is lowest at the bottom of the sequence (THEODORA-THABU; TCI: 0.6–0.9), whereas QUENTIN and QUINCY have the highest values (1.8–2.7). These trends are associated with a focus on backed pieces at the base of the HP and much reduced production of these types at the top. Whether this reflects a decrease in tool standardization can be checked by analyzing the CVs of metrics for backed pieces. The sample size corrected CVs (CV*(1 + 1/4n); [98] for all backed pieces for length, width and thickness made only on dolerite contradict this hypothesis: The CVs are generally lowest for the uppermost assemblages (QUINCY-QUENTIN; CVlength = 23.1%; CVwidth = 22.8%; CVthick = 31.4%) and highest for the bottom (THEODORA-SARAH; CVlength = 31.3%; CVwidth = 24.6%; CVthick = 35.4%), suggesting that while the overall production of backed pieces decreases through time, their standardization slightly increases. Looking at absolute sizes of backed pieces on dolerite throughout the sequence (n = 221) reveals a similar picture (see S1 Dataset for all measurements of retouched pieces). Whereas the bottom of the sequence features the largest backed pieces on average in all dimensions (THEODORA-SARAH; Length = 40.7 mm; Width = 14.5 mm; Thickness = 4.8 mm), the uppermost layers yield the smallest (QUINCY-QUENTIN; Length = 36.4 mm; Width = 12.8 mm; Thickness = 4.4 mm), with the intermediate layers lying in between (SAMAN-ROBERT; Length = 40.1 mm; Width = 13.8 mm; Thickness = 4.5 mm). This size reduction (S5 Fig in S2 File) is not found to be statistically significant by Kruskal-Wallis tests for length (H = 0.692; p = 0.708), thickness (H = 1.477; p = 0.478) and width (H = 5.641; p = 0.06) likely due to the small sample sizes in QUINCY-QUENTIN (n = 20; n = 7). The general size changes match with the variation in blade metrics that form the majority of tool blanks. A profound difference in sizes of backed pieces between raw materials, with quartz and crystal quartz being the smallest and truly microlithic in size (mean length = 17.3 mm, mean width 8.2 mm) and dolerite the largest (mean length = 40.0 mm, mean width = 14.1 mm), have previously been discussed in more detail [77] and are confirmed here.

There are some final differences in the use of raw materials for producing backed pieces, but not bifacial points, in the HP stratigraphic column. For backed pieces, QUENTIN and QUINCY show an almost exclusive use of dolerite with an absence of other raw materials, whereas assemblages below feature backed pieces also on hornfels, CCS, crystal quartz and quartzite. To some extent this might reflect absolute sample size, yet the pattern fits with an increased size standardization of backed pieces towards the top of the HP at Sibudu. In contrast, bifacial pieces are strongly associated with quartz in all layers, with only few other specimens on dolerite (n = 6), crystal quartz (n = 2), CCS (n = 1), and quartzite (n = 1).

### Additional lithic measures

**Fig 9:**
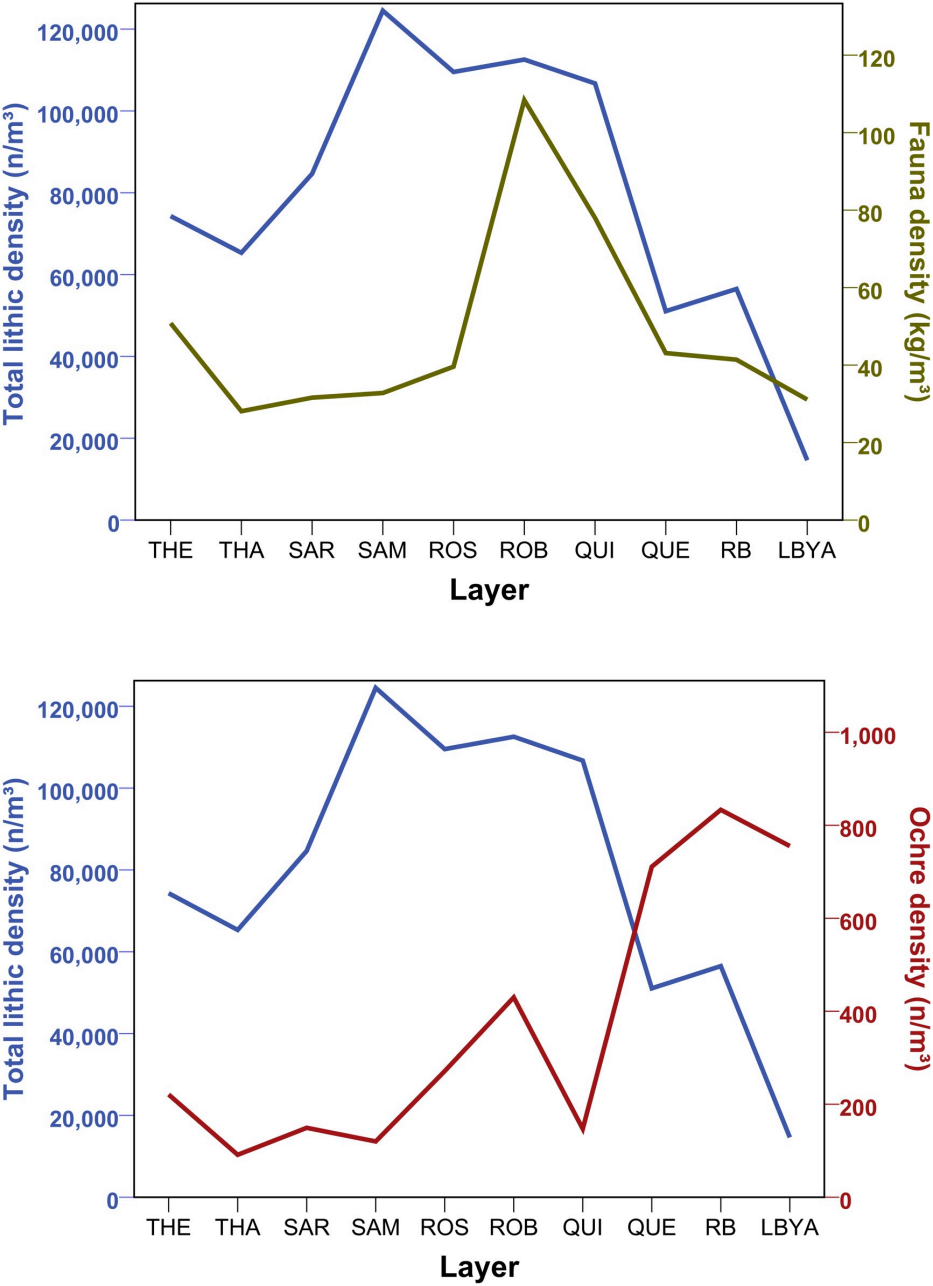
Diachronic variation of lithic, faunal and ochre densities throughout the HP at Sibudu. THE = oldest layer; QUE = youngest layer.

Calculation of reduction intensities—measured as blank/core ratio [110] and the average size of cores and blanks [110, 111]–do not find clear diachronic trends. The size measures support a scenario in which the bottom layers (THEODORA-SARAH) are the largest and least reduced, with the middle and upper part of the HP showing more intense reduction. The blank/core ratio backs this scenario for the uppermost assemblage QUENTIN, but THEODORA also yields similar values. In terms of total lithic find density (Fig 9), the HP at Sibudu has a peak in the middle and upper part (SAMAN-QUINCY: 100,000–116,000 n/m^3^) followed by the lowermost assemblages (THEODORA-SARAH: 59,000–78,000n/m^3^). Layer QUENTIN shows the lowest densities (48,000 n/m^3^) and a sharp drop compared to QUINCY (Fig 9). All of these densities are very high (S1 Table in S1 File) with the layers reflecting dense accumulations of lithic remains. Comparing lithic with faunal densities (kg/m^3^), yields a slightly different pattern (Fig 9) in which most assemblages have similar values except for peaks in ROBERT and QUINCY (78–108 kg/m^3^). Yet, the drop in densities between QUINCY (78 kg/m^3^) and QUENTIN (43 kg/m^3^) is repeated. The comparison of ochre (n/m^3^) with lithic densities and faunal remains reveals a different picture (Fig 9), with ochre density in the uppermost HP assemblage (QUENTIN: 711 n/m^3^) whereas the bottom part of the sequence (THEODORA-SAMAN: 91–221 n/m^3^) yields the lowest values.

### Multivariate analyses

**Fig 10:**
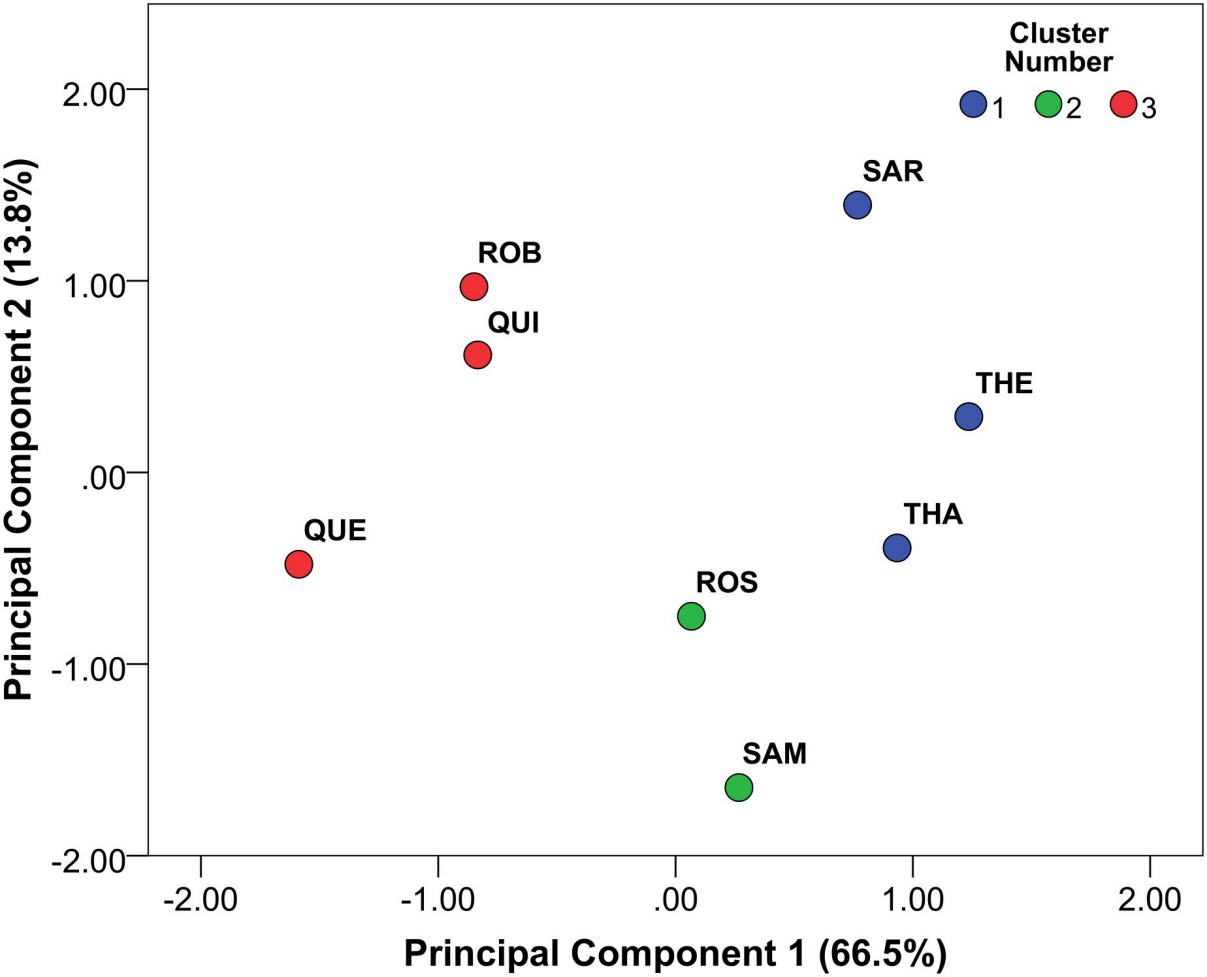
Results from the PCA analyses (PC1 and PC2), with individual assemblages as points labelled by their respective cluster number from the *k*-means cluster analysis.

A final test of diachronic trends throughout the HP of Sibudu was performed by examining key variables from the lithic assemblages in combination (dolerite%; sandstone%; quartz%; Tool%; Blade%; Flake%; Bladelet%; BifacialPoint%; BackedPiece%; MeanBladeLength; MeanBladeWidth; ShatteredBulb%; Lipping%; AbsentBulb%; EPA) via cluster analysis and PCA. Opting for a number of 3 partitions in the *k*-means cluster analysis supports a temporal trend in combining THEODORA-SARAH (Cluster 1); SAMAN-ROSA (Cluster 2) and ROBERT-QUENTIN (Cluster 3; see S11 Table in S1 File). Entering the same variables in a PCA and displaying the resulting indices (PC1 and PC2) for each HP layer with the attribution of the cluster analyses (Fig 10) underscores the diachronic partition. The order of the HP stratigraphic sequence is almost completely reconstructed by PC1 that explains 66.5% of the total variance. Negative loading on PC1 by individual components is particularly achieved (ordered by magnitude) by the proportion of flakes, sandstone, mean EPA and bifacial point percentage (S12 Table in S1 File). In contrast, positive loading on PC1 is mostly influenced by proportion of blades, lipping, dolerite and absence of bulbs. The largest positive influence on PC2 is found in mean blade length and width, with the strongest negative loading on the proportion of backed pieces.

## Discussion

### Diachronic change in the HP of Sibudu

Based on the rich and high-resolution stratigraphic sequence of Sibudu, we presented a statistical test of temporal trends within the HP from Conard’s excavation. Employing a quantitative assessment of lithic attributes on large samples allows us to detect directional trends and gradual change, if present. What is the extent and pattern of temporal changes in lithic technology during the HP at Sibudu? The quantitative results from our analyses of 8 layers of the Sibudu HP (n = 7831 lithics) document abundant change in the frequency of variables across all principal technological domains of stone knapping [*sensu* 97], including raw material procurement, core reduction, blade/flake production and tool manufacture. This also applies to more fine-grained observations such as flaking traces related to knapping techniques and other measures such as lithic densities. Data on individual *Abträge* reproduce this picture of abundant variability (S1 & S4 Figs in S2 File). Distortion of these results by different analysts or incommensurable attribute identification can be precluded since the assemblages were studied exclusively by one researcher (MW).

**Fig 11:**
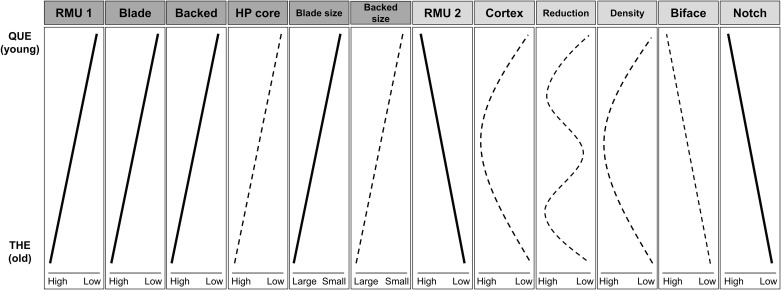
Schematic overview of diachronic trends in the HP sequence at Sibudu. Trajectories of the lines are based on descriptive information (increase, decrease, one peak, multimodal/curving), analytical results are reflected in the type of lines (dashed lines = not significant; bold lines = significant). Blade size = Blade length and width; Backed size = Backed piece length and width; RMU 1 = Dolerite; RMU 2 = Sandstone; Reduction = Assemblage reduction intensity; Density = Lithic and faunal find density.

Our statistical analyses of the HP lithic assemblages at Sibudu find consistent and significant trends in most of the key techno-typological markers. Fig 11 provides a schematic overview on all trends, distinguishing between statistically significant and non-significant patterns. The temporal trends align to show a picture of a consistent decrease in typical HP elements throughout the sequence. This includes the proportion of blades, backed pieces, and HP cores, as well as declining size of blades and backed artifacts. These conclusions are supported by the multivariate analyses. Interestingly, frequencies of bifacial pieces and use of coarser-grained raw materials increase throughout the sequence. Only a few measures, such as the shape of backed pieces, the economy of raw materials, cortex values, find densities, and reduction intensities do not show consistent trends. This, however, also implies that reduction intensities and reduction stages of individual assemblages cannot explain the observed changes.

How do these results compare to previous work within the HP of Sibudu? Our techno-typological and raw material results are in broad agreement with earlier technological and mostly qualitative work of Wadley’s HP sequence at Sibudu. As one example, bipolar technology is frequent and mostly associated with quartz throughout the HP in both this study and inWadley’s excavation [68, 69]. High frequencies of bipolar knapping appear to distinguish the HP of Sibudu from other sites [69] where this technology is usually rare [e.g. 82]. Another similarity concerns the abundance of bifacial points as a characteristic of the later HP at Sibudu [67], not the earliest HP as at Diepkloof [42]. While our study found abundant and directed diachronic variability, this apparent discrepancy to previous work can be explained by a different focus with maximal technological reconstruction of individual assemblages as key goal [e.g. 49, 68–70]. We do, however, find comparable trends in raw materials used for segments to Wadley [76] and Wadley and Mohapi [77]. Wadley and Mohapi [77: 2603] mention that backed pieces are most frequent at the base of the HP and our results confirm this observation. In contrast to Wadley and Mohapi [77], we found that size for segments decreases through times when corrected for raw materials (i.e. only looking at dolerite). At present, we cannot explain the large differences in the percentage of backed pieces for the uppermost HP between our study (38.5%) and Soriano *et al*. [49] with 65%, or the divergent observation of a decrease vs. increase in blade width. This discrepancy could have its roots in the different methods and sampling strategies between the two studies.

### Regionally divergent patterns of temporal change characterize the HP of southern Africa

Is the pattern of temporal changes in technology uniform across the HP sequences in southern Africa? Recent research has suggested synchronous changes across the HP in southern Africa [e.g. 40, 42, 71]. A comparable effort to compare diachronic trends has been undertaken by Douze *et al*. [82] based on the mostly qualitative comparison of the HP from Klipdrift with six other sites. Here we build and expand on this important work by focusing on a more quantitative and statistical treatment of five comparable variables. We gathered relevant numerical data from a total of seven HP sites with three or more stratigraphic units (see Table 8) and performed the same statistical analyses on lithic attributes as for Sibudu. These traits include information on the main (fine-grained) raw material used (“RMU1”), the proportion of blades, and the abundance of backed pieces, which are considered as primary indicators of HP techno-typological systems [e.g. 65, 68, 82]. Additionally, we analyzed data on the secondary main (coarse-grained) raw material used at the sites and frequencies of notched and splintered pieces. Results, test statistics and graphics for these comparisons for individual variables by site can be found in S2-S8 Text in S3 File. Quantitative attribute data on technological aspects such as platform treatment and blade metrics were not available for many of the comparative sites. This narrowed down comparable data to a few central characteristics that have been gathered for all localities.

**Table 8:** Overview of HP sites used for the comparative quantitative study with reference to sources of the numerical lithic data and absolute dating.

Site	HP layers (n)	Deposit thickness (in m)	Numerical data?	Sequence age	Reference
Diepkloof	24	1.5	RMU; Blanks; Tools; Blade & backed piece dimensions	110–57 ka (Tribolo) / 65–56 ka (Jacobs & Roberts 2017)	Lithics: [42]; Dating: [52, 57]
Klipdrift	7	0.7	RMU; Blanks; Tools; Blade dimensions; Find densities; Cortex	66–59 ka	Lithics: [82]; Dating: [112]
Klasies River	12 (Cave 1A, 10–21)	1.5	RMU; Blanks; Tools	67–63 ka	Lithics: [36, 47]; Dating: [57]
Klein Kliphuis	12 (spits)	0.4	RMU; Tools; Blanks (elongation)	66–60 ka	Lithics: [29, 113]; Mackay own data; Dating: [19]
Rose Cottage Cave	4	0.3	RMU; Blanks; Tools; HP cores; Knapping technique; Blade dimensions	65–63	Lithics & Dating: [81]; Dating: [19]
Pinnacle Point 5–6	4/5	2.3	RMU; Blanks; Tools; Blade & segment dimensions	73–56 ka	Lithics: [95]; Lithics & Dating: [95, 114];
Umhlatuzana	5 (spits 22–26; but potential mixing)	0.6	RMU; Tools; Blanks	~60 ka	Lithics: [80]; Dating: [115]

**Fig 12:**
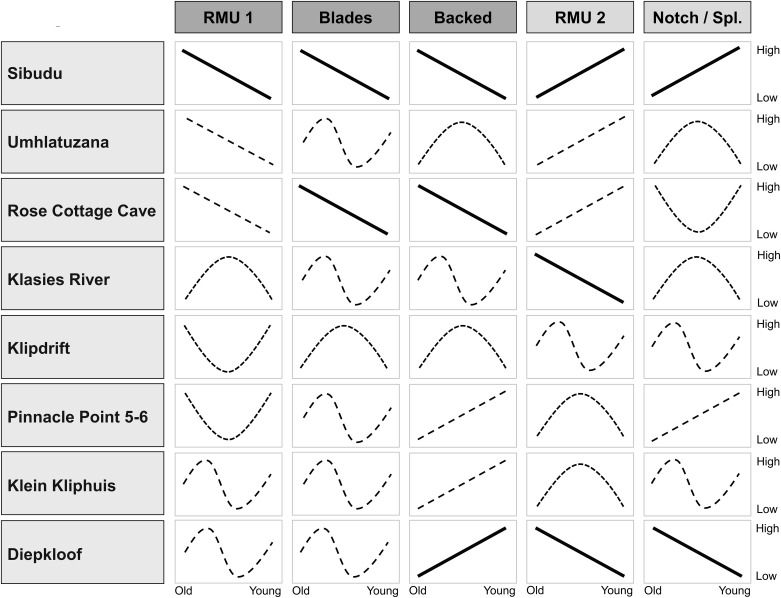
Schematic overview of regional diachronic trends in the HP of South Africa. Trajectories of the lines are based on descriptive information (increase, decrease, one peak/minimum, multimodal/curving), analytical results are reflected in the type of lines (dashed lines = not significant; bold lines = significant). RMU 1 = Most frequent raw material (fine-grained); RMU 2 = Second most frequent raw material (coarse grained). Notch/Spl. = Notches & denticulates / Splintered pieces (depending on site).

The composite picture of diachronic trends for the seven HP sites for five quantitative attributes in comparison with the Sibudu sequence is shown in Fig 12. The lines summarize the combination of descriptive (temporal patterns) and analytical (statistical significance) information. Only 11 out of 40 diachronic trajectories reach p<0.05 in their Kendall rank correlation coefficients. Descriptively, chronological trends could be divided into unidirectional increase, unidirectional decrease, U-shaped trajectories with one peak/minimum and a multimodal pattern with two or more peaks separated by multiple local minima.

The composite diagram (Fig 12) shows high variability in temporal trajectories for the main HP variables between sites. Localities in the summer rainfall zone (SRZ) of the eastern part of South Africa—Umhlatuzana and Rose Cottage Cave—show overall the closest trajectories to Sibudu, which might be a function of their geographical proximity. This being said, the HP at Umhlatuzana differs from close-by Sibudu in showing non-directional trends for the proportion of blades and backed pieces, without a clear decrease throughout the sequence. Sites in the year-round rainfall zone (YRZ) of southern South Africa include the long sequences of Klasies River, Klipdrift and Pinnacle Point 5–6 (PP5-6). These localities show abundant variability, often with one or multiple peaks, without any significant unidirectional diachronic trends within the HP for any of the studied variables (except for a decrease in quartzite at Klasies River). The YRZ sites show a tendency for some HP markers to cluster in the middle of the sequences, but not consistently. The amount of backed pieces at PP5-6 increases through time, providing a picture opposite to Sibudu. In the winter rainfall zone (WRZ) of the Western Cape, the HP sequences of Diepkloof and Klein Kliphuis are comparable to one another in their diachronic trends. They show abundant fluctuation in silcrete use and blade proportions, but consistent and nearly significant increases in the frequency of backed pieces towards the top of their sequences [see also 83]. Combined with the proportion of other raw materials and notches/splintered pieces, the WRZ sites provide a picture opposed to the temporal trends in raw material, technological and typological markers at Sibudu and the SRZ sites. Whereas the youngest HP at Sibudu shows the weakest markers of this technocomplex, the Late HP at Diepkloof constitutes its most classic manifestation, such as the highest frequencies of backed pieces and typical HP blade cores [e.g. 42]. In sum, these results diverge from the expectations of uniform temporal variability in the HP of southern Africa. Instead we find a pattern of heterogeneous diachronic change across sites and regions.

These conclusions are at odds with a recent qualitative assessment by Douze *et al*. [82], finding comparable trends in knapping traces and other technological markers across Klipdrift, Rose Cottage Cave, Diepkloof and Klasies River. Their conclusion of “strong recurrences in the patterns of changes during the HP over large spatial scales” [82: 20] was not corroborated by this quantitative study. This being said, Douze *et al*. [82: 16] also report on some qualitative differences between the studied HP sites and particularly between the sequences of Klipdrift and Sibudu also found here. Our results are also in contrast with Villa *et al*. [40], who found similar changes for blade metrics, knapping technique, decrease in standardization and increase in the frequency of flakes at Klasies River and Rose Cottage over time. While these changes might well be comparable for their samples, we found different diachronic patters for changes in the frequencies of blades, backed pieces and the most used (fine-grained) raw materials (Fig 12) based on a quantitative comparison with the data from the Singer and Wymer [36] sequence (also [47: Appendix 2], see S4 Text in S3 File) and data by Soriano *et al*. [81]. Instead of a “gradual and parallel process of change” [40: 654], we identify consistent but regionally variable patterns. Also, Sibudu does not show a consistent decrease in standardization of blades over time, and the size decrease in blades and backed pieces is not mirrored at Klasies (largest pieces in the lower middle part [47: Table 94]), Diepkloof (largest pieces in the middle [42]), Klipdrift, and PP 5–6 (largest pieces at the top [82, 114]). Interesting in this regard is the emergence of an independent form of flake manufacture as a unifying feature among many assemblages towards the end of the HP at Diepkloof [42], Rose Cottage Cave [81], Klasies River [40] and Klipdrift [82]. At Sibudu, this observation finds quantitative support in the form of an increased share of flakes among all blanks and tools through time.

Our results fit with one of the few quantitative inter-site analyses of HP technology by Clarkson [66]. He found regional traditions (SRZ, WRZ and YRZ) of core reduction methods based on a multivariate morphometric analysis of cores from five sites. In a similar vein, Mackay *et al*. [65: 36] in their large-scale meta study of the MSA in South Africa observed complex temporal trends for backed pieces in the WRZ and YRZ, and noted potential differences to the SRZ. Finally, our results back a recent assessment of the HP as “multimodal, suggesting a sequence of on-going adaptive responses, rather than a single static entity” [83] which was based on a quantitative comparison of Klein Kliphuis and Diepkloof in the WRZ. Our study documents the expansion of this pattern to the YRZ and SRZ, and underscores the complex variability in this technocomplex. Similar observations of variable “abrupt shifts, gradual shifts and time-restricted shifts” [82: 14] for HP markers have been made for the Klipdrift sequence. From an intra-site perspective, these asynchronous and intricate trends at Klipdrift, Diepkloof and Klein Kliphuis do not conform to the mostly gradual and unidirectional changes at Sibudu and attest to variability in diachronic changes within the HP. Apart from stone tools, this pattern of distinct regional signals in material culture and local traditions fits the conclusion reached by a study of the specific bone tool technology at Sibudu during the HP and beyond [16].

Our statistical comparisons of HP sequences face a couple of challenges. One is the different reporting of lithic data, both in terms of its presentation (numerical vs. qualitative) and scale (assemblages vs. whole technocomplex). Ultimately this allowed only comparison of a handful of variables across all eight HP sequences and the exclusion of sites without such data. Another problem concerns the number of discernible HP layers. Their low number at all sites (n<30) does not allow for statistical testing of non-linear trends. The observed multimodal trajectories could encompass a mix of various patterns (e.g. quadratic or cubic) that might imply different factors at work such as cyclic adaptations, drift or noise. Finally, it is likely that not all sequences sample the entire HP, with many areas potentially missing its early parts (*sensu* [42]). Yet, we refrained at present from incorporating chronometric ages to our comparisons due to ongoing debates on the dating of the HP [52, 55–57], making chronological correlations of HP assemblages extremely difficult. Instead, we used a conservative approach by using relative stratigraphic order of layers at all HP sites, as have many others. We thus agree with recent assessments [18: 167, 68] that this problem of diverging ages is not solvable here, but emphasize that six out of seven HP sequences date roughly to the same period of ~70–60 ka (Table 8). Potential ways forward could be the comparative dating between laboratories of HP sequences, the quantitative study of data per individual assemblages, and the excavation of more sites to test the patterns found in this study.

### Multiple causes of behavioral change in the HP of southern Africa

How can we explain the consistent pattern of temporal change in lithic technology within Sibudu and the variable diachronic trends across the HP in southern Africa? The origins and abandonment of HP lithic technology have been variously explained by adaptations to environmental changes, including shifting territorial organization and mobility patterns [25, 58, 60], changes in subsistence and hunting [62, 63], increases in population size [20, 22, 64], or changing networks of information transmission between connected groups [65]. Table 1 provides the expectations and archaeological correlates of these hypotheses. Any of these hypotheses is conceivable, though multiple causal factors likely acted sequentially or in concert. Consequently, we follow the concept of multiple working hypotheses [116] (for archaeology see the exemplary work by G. Isaac [117]) which allows us to weigh and compare the explanatory potential of all of these ideas insofar as available data permit.

For Sibudu, rich contextual data exist to assess different hypotheses for causal factors behind observed behavioral changes. In terms of environmental causation (Table 1), various ecological proxies exist, but mostly on the scale of the technocomplex. Information from phytoliths, charcoal, seeds, and stable isotopes suggests thick evergreen forest with many closed-environment species and high humidity for the local environment of Sibudu for the HP [118–122]. Yet, layer-by-layer data on hunted macro-fauna suggest gradual opening of the local landscape during the HP dating to ~65–60 ka [84, 123]. In terms of subsistence, all HP layers show a dominance of smaller bovids preferring wooded habitats such as the blue duiker [124, 125], but there are consistent diachronic trends. Clark [84, 123] found significant differences between the lower (PGS) and middle (GS) vs. upper (GR) part of the HP in the proportion of open vs- closed-dwelling species. More open landscape prey in the upper part of the sequence correlates with a steady decline of small game. These slowly opening environments reflected in prey representation co-vary with the consistent techno-typological changes found here, as already suspected by Clark [84: 67]. The covariation of these factors suggests a feedback between changing prey types, reduced subsistence intensification and preferred lithic weaponry (e.g. decline in backed pieces), that ultimately led to the abandonment of this technology in the following Sibudan. This interpretation is strengthened by use-wear and residue studies suggesting the dominant use of backed pieces as inserts for (composite) hunting weapons such as in bow and arrow technology at Sibudu [75, 78, 126, 127]. In terms of environment and subsistence driving technological change within the HP, a different conclusion is reached at Diepkloof [42, 128]–here the use of backed pieces as weapons is also disputed [128]–and Klipdrift where “changes in lithic strategies are generally not synchronous with changes in subsistence behaviors or environmental conditions” [82: 19]. The expectations of this causal factor (Table 1) are not met at these sites.

The HP backed tools have also been interpreted as specific adaptations to ecological instability in MIS 4 [60, 61]. Testing this hypothesis is difficult, since paleoenvironmental data for HP sites are available on different levels of spatial and temporal resolution, complicating correlations between data sets [see 129–131]. Yet, diachronic frequencies of backed pieces vary strongly between localities (Fig 12), and site-specific data from Klipdrift and Diepkloof do not show any co-variation between paleoenvironmental indicators and technological change [42, 82]. These observations cast doubt on strict one-to-one ecological adaptations for these sites.

Variations in the organization of mobility and territoriality constitute another potential cause for changes in lithic technology (Table 1). At Sibudu, commonly used proxies for these factors such as reduction intensities, cortex patterns and densities of lithics, fauna and ochre show no clear co-variation with the main techno-typological changes. This observation suggests that mobility and site-use strategies had a minor impact on the overall diachronic trajectory of lithic technology in the HP of Sibudu. Only the top layer of the HP (QUENTIN) shows some expected signals of reduced mobility and lower site occupation intensity [*sensu* 132–135]: low densities of lithic and faunal remains, high amounts of sandstone suggesting a reduced procurement radius, and increased reduction intensity coupled with more expedient core reduction such as bipolar. Considering a recent model of reduced mobility as an explanation towards more expedient lithic strategies across the interface of the HP and the following Sibudan [73], this trend may have already begun at the end of the HP. At Klasies River and Rose Cottage Cave, there is a tendency towards more expedient technology and ephemeral occupations at the top of the HP [e.g. 40] and Klasies River also shows a lower abundance of non-local tool stone in the youngest layers. These data suggest a more circumscribed mobility radius that provides some links to Sibudu. Yet, these patterns are not found for the WRZ sequences at Diepkloof, Klein Kliphuis and PP 5–6. Here, increase of non-local tool stone at the top of the HP sequences is associated with another increase of formal blade technologies and production of backed pieces but also more intense stays at the sites, as shown by geoarchaeological work [136, 137]. These observations contradict the general expectations of a reduced procurement radius [134, 135, 138–141].

Demography has been of particular interest as a driver behind behavioral change in the MSA [22, 64], but is difficult to assess from the archaeological record alone [142]. Reconstructions of (effective) population sizes from recent and ancient DNA of southern Africa open up a new and promising route [143, 144] but data are so for limited in quantity and only available for the Holocene. At Sibudu, indirect archaeological measures of population sizes such as reduction intensities and find densities do not covary with technological change. Frequent trampled hearths, bedding and high density of stone artefacts indicate intense site use during the entire HP [90, 145]. While the uppermost HP layer shows less intense occupations, the overall trajectory in lithic technology at Sibudu is not driven by major changes in population size based on measures of reduction intensity and find density. Demographic estimates from recent and ancient DNA in southern Africa provide a picture of decreasing and low population sizes toward the end of MIS 4 [143, 144] that could fit data from the uppermost HP at Sibudu, but subtle temporal changes on millennial scales and potential regional differences are currently not resolved. No other HP sites have provided comparable lithic density values (S1 Table in S1 File), yet geoarchaeological work at Diepkloof finds a marked change in the mode of occupation within the Late HP [136]. This phase is characterized by more intense stays potentially by more people and associated with an increase of blade technology and backed pieces. At Klipdrift, periods of intense occupation covary with increased use of HP technology [82, 146]. At PP5-6, higher find densities and occupation intensity are likewise interpreted as potential increase of group size [95, 137]. In conclusion, there is some evidence for an expected correlation (Table 1) between more intense occupations and increased levels of blade technology, backing and non-local tool stone procurement for sites in the WRZ and YRZ. However, such correlations are not found for sites in the SRZ and genetic estimates of effective population sizes across southern Africa [143, 144] cast general doubts on this hypothesis.

On a regional, inter-site level, we can evaluate the expectations of different connection networks of shared information between groups effecting changes in lithic technology (Table 1). The distinct temporal trajectories at sites in the spatially removed WRZ and SRZ conform to expectations of higher connectivity *within* but reduced connections *between* these regions. This trend appears to increase particularly towards the end of the HP. A reduced exchange of information between groups over time fits with the pattern of even more regional variability in lithic technology seen in early MIS 3 [e.g. 65]. To further this interpretation in future studies, cultural transmission theory [147–151] could be applied to cases like Sibudu based on the observed directional shifts in stone knapping behaviors that are likely related to mechanisms of information transmission (see e.g. for the Still Bay: [152]).

In conclusion, identifying causes behind the heterogeneous patterns of temporal variation between HP sequences constitutes a difficult task. Relevant data to test expectations (Table 1) are not available at the necessary level of resolution for all sites, and different HP sequences provided different correlations with potential causal factors. Yet, a notable feature of the temporal variation between HP sites is the partial alignment based on geographical proximity and paleoecological zones. This co-variation could indicate that specific ecological adaptations drove technological change. At Sibudu, this interpretation matches the co-variation of high-resolution faunal and lithic data. On a regional level, there is a continuation and even increase of HP technologies in areas with stable climate in MIS 4 (WRZ), whereas more variable regimes show gradual abandonment (SRZ). In this line of reasoning, spatially distinct selection pressures from environmental circumstances would have led to comparable behavioral adaptations within these regions, coupled with increased connectivity of groups within these areas [e.g. 65, 82, 152]. Yet, even within the HP we see asynchronous changes that do not align. Different technological markers of the HP sometimes change contemporaneously, such as at Sibudu, yet show distinct temporal trajectories at other sites such as Diepkloof or Klasies River (Fig 12). This pattern of divergent and at times decoupled changes precludes a single, all-encompassing explanation. Instead these observations point towards more complex causal scenarios. In sum, this study was able to reject several previous causal hypotheses for technological change in the HP on a site-by-site level and identify multiple drivers for temporal change on a regional scale.

The findings of our study suggest some future lines of inquiry that might be fruitful. Studies of long-term behavioral change should consider historical contingency, path dependence, multicausality and emergent properties. Complexity theory has shown that different initial (technological) starting conditions in any historical system can lead to drastic, unpredicted and qualitatively different outcomes down the line [153–156]. Tracing the evolutionary sequences of change within the HP across multiple scales thus helps to understand the causes and long-term consequences of behavioral change. To this end, behavioral adaptations can be conceived within multidimensional fitness landscapes [157]: the landscapes feature multiple adaptive peaks and valleys of different height shaped by social, demographic and environmental parameters [13, 158–160], instead of single optimal solutions to ecological challenges as is often assumed. People during the HP shared general behaviors, but varied their technological strategies based on different ecological and socio-cultural circumstances that they had to deal with. As a result, multiple and divergent evolutionary trajectories materialized, such as the ones that we observed between HP sequences. Given this complex nature of temporal changes, the answer regarding causal drivers of behavioral change in the HP likely lies in a combination of factors acting in different ways on contingent technological trajectories. Testing such models requires more small-scale and spatially-sensitive studies with multifaceted archaeological and paleoenvironmental data of high resolution from well-dated stratigraphic sequences.

## Conclusion

Consistent diachronic changes characterize the HP sequence of Sibudu, with a decrease of typical lithic markers such as the frequencies of backed pieces and blades. Additionally, different temporal trends exist for the HP between sites and regions within southern Africa based on a set of techno-typological variables currently available for direct quantitative comparison. Our results help to inform debates about the multiple causes driving these behavioral changes and to better assess the variable end of the HP across regions.

Research on the HP in the past decades has tended to view this technocomplex as a monolithic block indicative of early complex culture. This view was first popularized in models of cultural evolution within southern Africa [19, 21, 44]. Subsequently, some scholars exported and borrowed these ideas on larger scales of Africa and beyond [23–25, 27, 64]. These studies have viewed the HP as a cultural unit with high behavioral complexity in contrast to the more variable and changing nature of the archaeological record before and after (e.g. the “post-HP”). There is now growing evidence from multiple strands of research that this picture is not correct and requires refinement based on new empirical and theoretical observations [18, 28, 31, 33, 40, 42, 65, 72, 152]. To this list we can add the regional and intricate pattern of technological changes through time in the HP. While the HP as a technocomplex has certain unifying features across southern Africa [e.g. 49, 65, 68] it is much more variable in its lithic and non-lithic expressions [e.g. 16] in space and time than was previously thought. Central features of the HP, such as the frequency of producing blades and backed pieces, show different temporal trends among sites.

This intricate pattern of diachronic change suggests that diverse factors were responsible for the appearance and disappearance of HP technologies across regions. Some of this variability can be associated with regional adaptations to different ecological circumstances based on functional needs. Subtler differences in the rhythm of change between sites of the same region more likely reflect variable organization of technology and mobility, demographic variables, and dynamics of cultural transmission. The record of the HP in southern Africa, a well-resolved and well-studied technocomplex in the MSA, serves as a cautionary tale against neglecting variability in favor of big-picture scenarios and master narratives. In order to assess patterns of behavioral change in the HP of southern Africa and the MSA more generally, researchers will need a better baseline of comparable high-resolution data on lithic artefacts.

## Supporting information

S1 File Supporting information S1-S12 Tables. (DOCX)

S2 File Supporting information S1-S5 Figs. (DOCX)

S3 File Supporting information S1-S8 Text. (DOCX)

S1 Dataset Raw data on formal pieces and blades. (XLSX)
