# Supplementary material for: Regional patterns of diachronic technological change in the Howiesons Poort of southern Africa
Source: PLoS One. 2020 Sep 17;15(9):e0239195. doi: 10.1371/journal.pone.0239195 (PMC7498030; doi:10.1371/journal.pone.0239195)

**SUPPLEMENTARY INFORMATION FIGURES S1-S5**

**Figure S1.** Diachronic distribution of blank types by *Abtrag* in the HP of Sibudu.


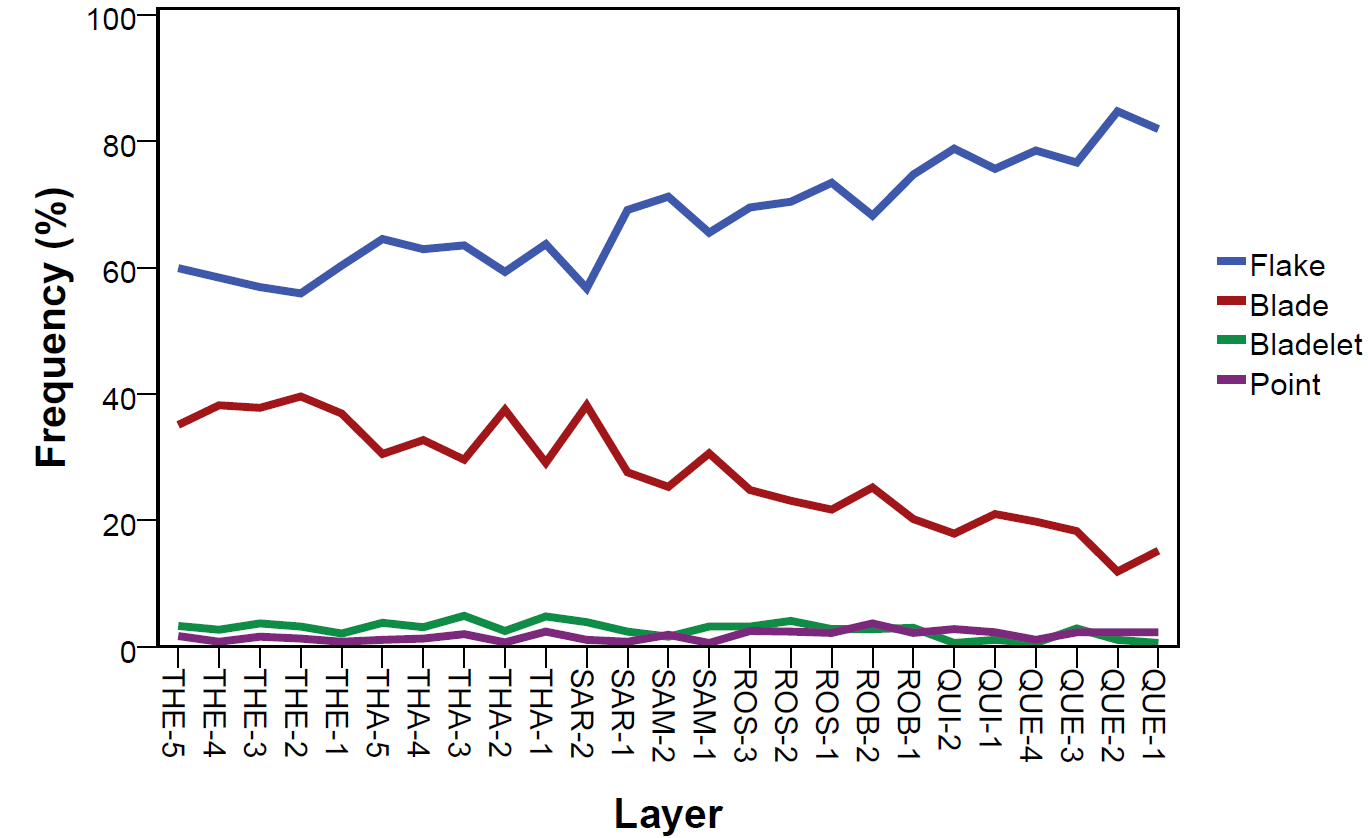


**Figure S2.** Box plot comparison of blade width by 2-layer assemblages for the HP at Sibudu.


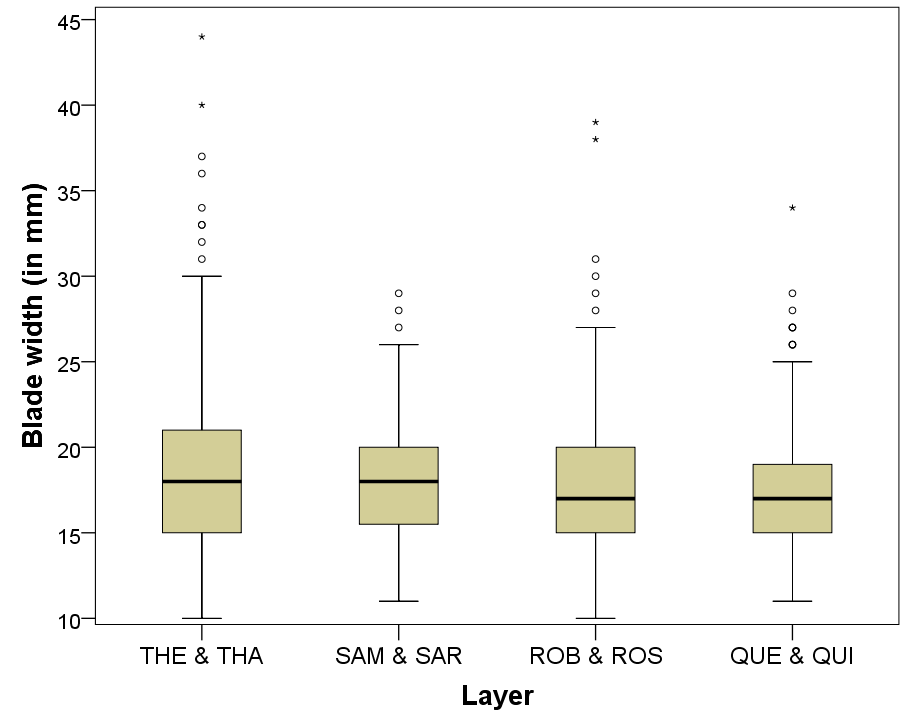


**Figure S3.** PCA for knapping technique attributes with indication of group membership by multivariate *k*-means cluster analyses.

**
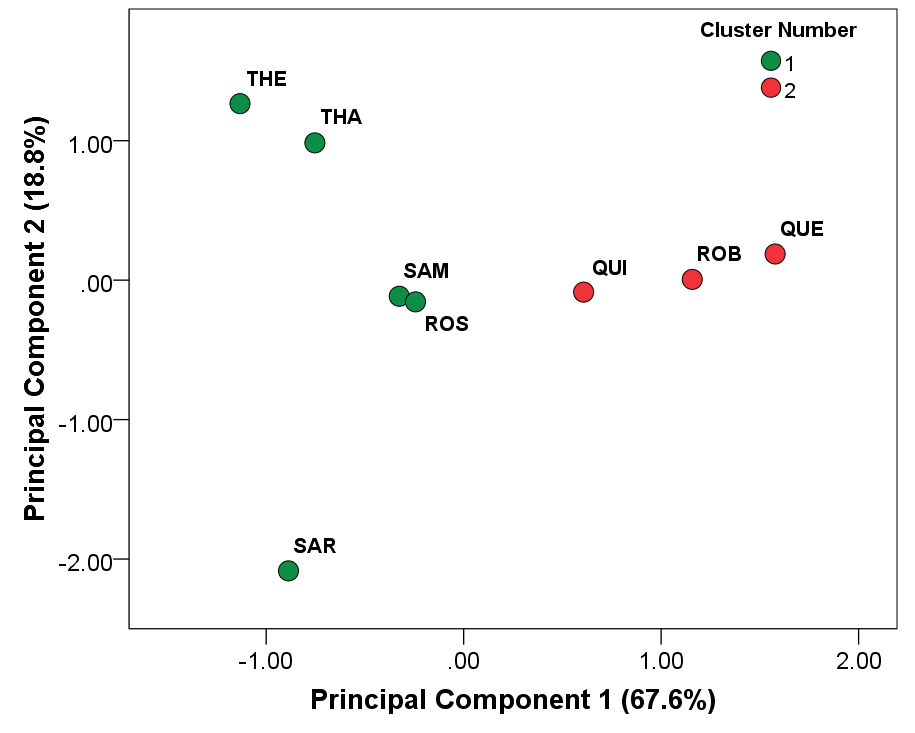
**

**Figure S4.** Diachronic distribution of classic tool types by *Abtrag* in the HP of Sibudu. Note the small sample size (n) for some of the *Abträge* (see SOM Table 2).

**
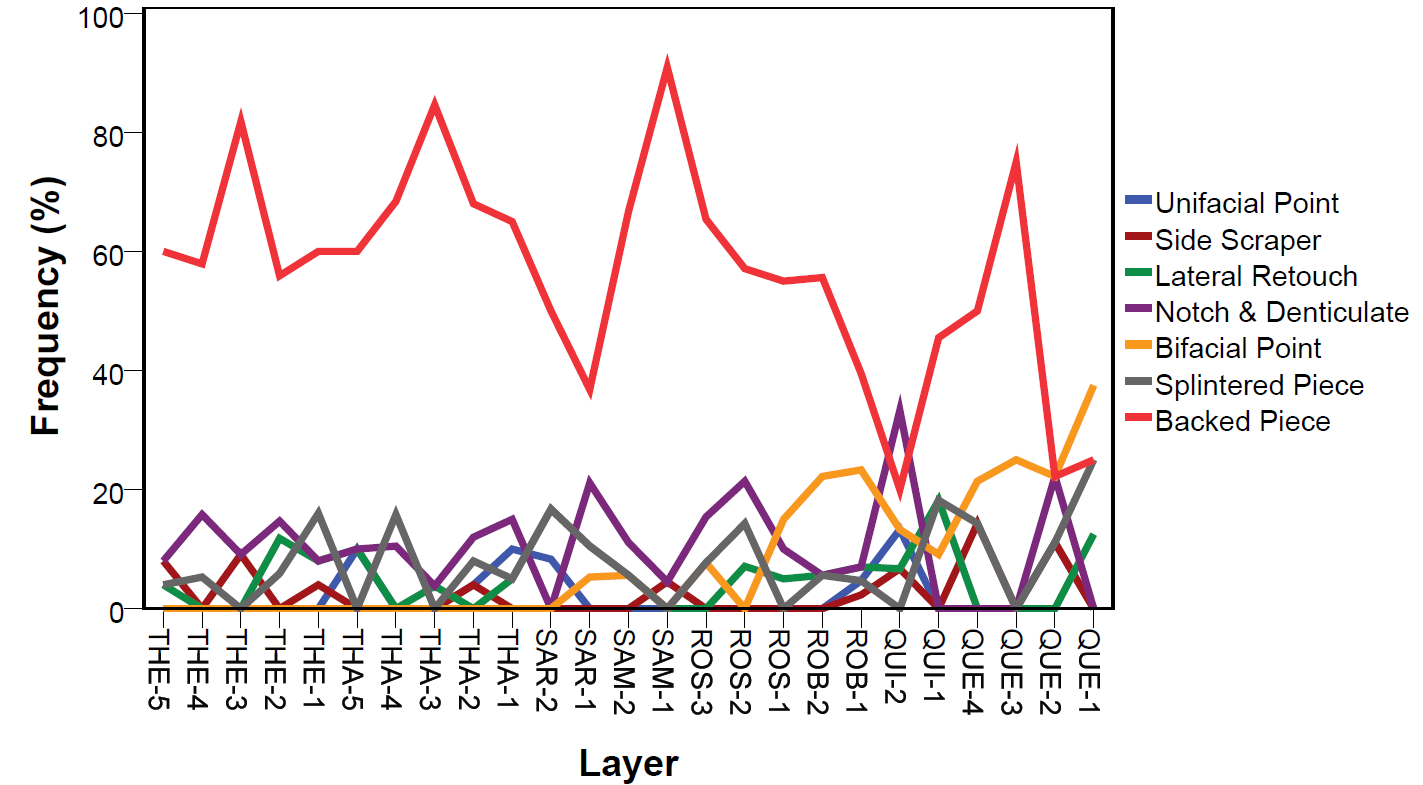
**

**Figure S5**. Box plots of Kruskal-Wallis tests for length (H=0.692; p=0.708), thickness (H=1.477; p=0.478) and width (H=5.641; p=0.06) of backed pieces.


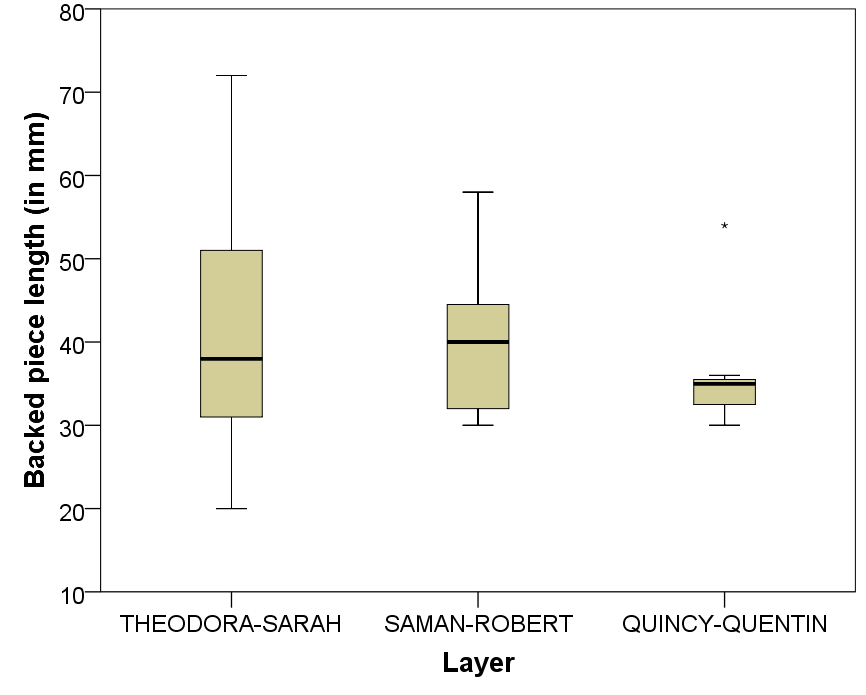


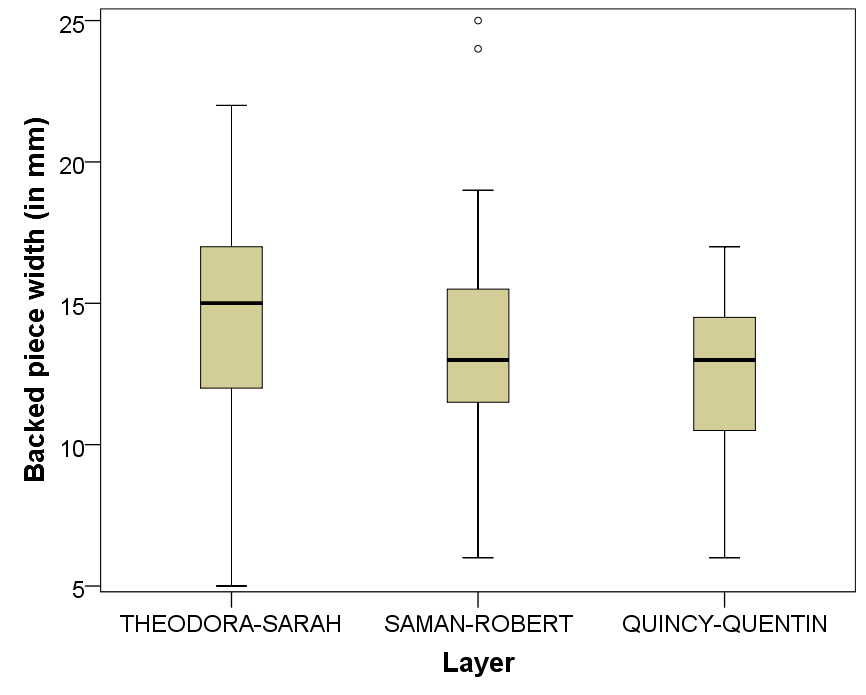


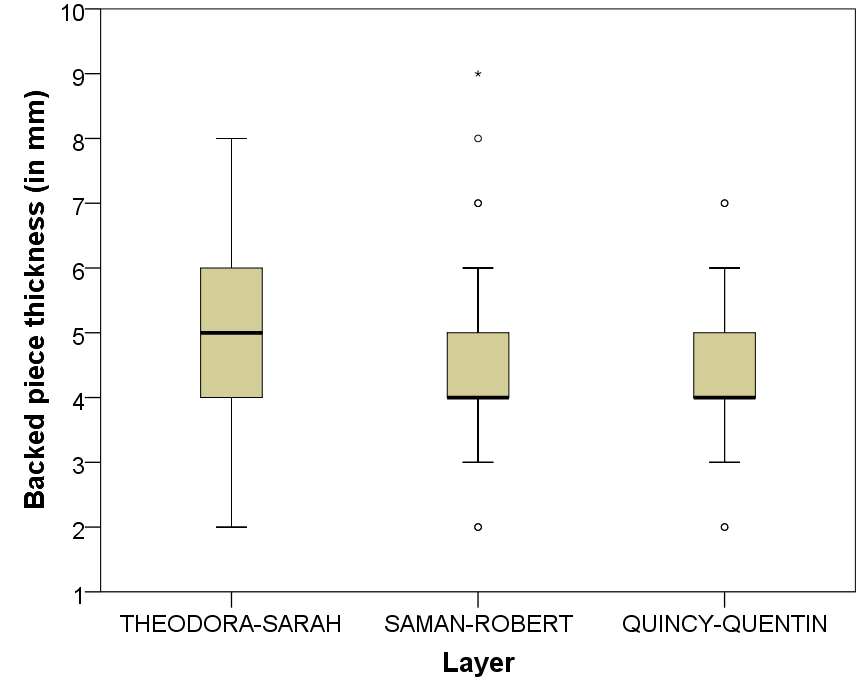

Supplement: S1 File — (DOCX) [file pone.0239195.s001.docx]
