# Supplementary material for: Regional patterns of diachronic technological change in the Howiesons Poort of southern Africa
Source: PLoS One. 2020 Sep 17;15(9):e0239195. doi: 10.1371/journal.pone.0239195 (PMC7498030; doi:10.1371/journal.pone.0239195)
Supplement: S2 File — (DOCX) [file pone.0239195.s002.docx]

**SUPPLEMENTARY INFORMATION TABLES S1-S12**

*METHODS*

**S1 Table.** Comparison of published lithic density values (n/m³) between MSA sites.

| **Site** | **Technocomplex/Age** | **Lithics n/m³** | **Reference** |
| --- | --- | --- | --- |
| Hoedjiespunt 1 | Early MSA (MIS 5e) | 933-4146 | Own calculation |
| Pinnacle Point 13B | MIS 6-5 | 14-4165 | Marean 2010 |
| Sibudu | MIS 4 (HP) | ~51,000-124,000 | This study |
| Klasies River | MIS 5 (MSA II) | 49,463-51,692 | Brenner & Wurz 2019 |
| Blombos Cave | MIS 5-4 (“pre-SB”; SB) | 2935-30,208 | Henshilwood et al. 2001 |

*RESULTS*

**S2 Table.** Results of Chi-Square test for raw material, comparing the numerical distribution of sandstone and dolerite by layer. Note the distribution of adjusted residuals throughout the sequence (e.g. for dolerite from highly negative in QUE to highly positive in THE). Significant associations (adjusted residuals >1.96, Agresti 2007; Sharpe 2015) marked in bold, found for 5 out of 8 HP layers.

| **AH * RMU Crosstabulation** | | | | | |
| --- | --- | --- | --- | --- | --- |
|  | | | RMU | | Total |
|  |  |  | Dolerite | Sandstone |  |
| AH | QUE | Count | 614 | 310 | 924 |
|  |  | Expected Count | 711.1 | 212.9 | 924.0 |
|  |  | Standardized Residual | -3.6 | 6.7 |  |
|  |  | Adjusted Residual | **-8.1** | **8.1** |  |
|  | QUI | Count | 286 | 135 | 421 |
|  |  | Expected Count | 324.0 | 97.0 | 421.0 |
|  |  | Standardized Residual | -2.1 | 3.9 |  |
|  |  | Adjusted Residual | **-4.5** | **4.5** |  |
|  | ROB | Count | 464 | 207 | 671 |
|  |  | Expected Count | 516.4 | 154.6 | 671.0 |
|  |  | Standardized Residual | -2.3 | 4.2 |  |
|  |  | Adjusted Residual | **-5.1** | **5.1** |  |
|  | ROS | Count | 580 | 166 | 746 |
|  |  | Expected Count | 574.1 | 171.9 | 746.0 |
|  |  | Standardized Residual | .2 | -.4 |  |
|  |  | Adjusted Residual | .5 | -.5 |  |
|  | SAM | Count | 466 | 147 | 613 |
|  |  | Expected Count | 471.8 | 141.2 | 613.0 |
|  |  | Standardized Residual | -.3 | .5 |  |
|  |  | Adjusted Residual | -.6 | .6 |  |
|  | SAR | Count | 311 | 86 | 397 |
|  |  | Expected Count | 305.5 | 91.5 | 397.0 |
|  |  | Standardized Residual | .3 | -.6 |  |
|  |  | Adjusted Residual | .7 | -.7 |  |
|  | THA | Count | 1060 | 236 | 1296 |
|  |  | Expected Count | 997.4 | 298.6 | 1296.0 |
|  |  | Standardized Residual | 2.0 | -3.6 |  |
|  |  | Adjusted Residual | **4.6** | **-4.6** |  |
|  | THE | Count | 1651 | 339 | 1990 |
|  |  | Expected Count | 1531.6 | 458.4 | 1990.0 |
|  |  | Standardized Residual | 3.1 | -5.6 |  |
|  |  | Adjusted Residual | **7.5** | **-7.5** |  |
| Total | | Count | 5432 | 1626 | 7058 |
|  |  | Expected Count | 5432.0 | 1626.0 | 7058.0 |

| **Chi-Square Tests** | | | |
| --- | --- | --- | --- |
|  | Value | df | Asymptotic Significance (2-sided) |
| Pearson Chi-Square | 158.506^a^ | 7 | .000 |
| Likelihood Ratio | 154.482 | 7 | .000 |
| N of Valid Cases | 7058 |  |  |
| a. 0 cells (0.0%) have expected count less than 5. The minimum expected count is 91.46. | | | |

| **Symmetric Measures** | | | |
| --- | --- | --- | --- |
|  | | Value | Approximate Significance |
| Nominal by Nominal | Phi | .150 | .000 |
|  | Cramer's V | .150 | .000 |
| N of Valid Cases | | 7058 |  |

**S3 Table.** Raw material retouch index (RMRI; Orton 2008) for raw materials in the HP of Sibudu. RMRI values >1 indicate preference for retouch.

| **Raw Material** | **% tools** | **% total assemblage** | **RMRI** |
| --- | --- | --- | --- |
| Dolerite | 69.2 | 69.4 | 1.00 |
| Sandstone | 2.0 | 20.8 | 0.09 |
| Hornfels | 6.1 | 3.3 | 1.85 |
| Quartzite | 6.8 | 3.9 | 1.74 |
| Quartz | 9.4 | 2.1 | 4.47 |
| CCS & Other | 6.6 | 0.6 | 10.92 |

**S4 Table.** Number of cores and selected Conard et al. (2004) core types (n) by raw material for the HP of Sibudu. Note the high number for quartz (bipolar) cores.

| **Layer** | **Parallel** | **Platform** | **Inclined** | **Bipolar** | **TOTAL** |
| --- | --- | --- | --- | --- | --- |
| Dolerite | 11 | 45 | 1 | 3 | 61 |
| Hornfels |  |  |  | 1 | 1 |
| Sandstone | 1 | 4 |  |  | 5 |
| Quartzite |  | 2 |  | 5 | 7 |
| Quartz | 1 | 4 |  | 45 | 57 |
| CCS & Other |  | 3 |  | 1 | 4 |
| TOTAL | 13 | 58 | 1 | 55 | 127 |

**S5 Table.** Results of Chi-Square test for blank type, comparing the numerical distribution of blades and flakes by layer. Note the distribution of adjusted residuals throughout the sequence (e.g. for blades from highly negative in QUE to highly positive in THE). Significant associations (adjusted residuals >1.96, Agresti 2007; Sharpe 2015) marked in bold, found for 6 out of 8 HP layers.

| **AH * Blank Crosstabulation** | | | | | | | | | |
| --- | --- | --- | --- | --- | --- | --- | --- | --- | --- |
|  | | | | | Blank | | | | Total |
|  |  |  |  |  | Blade | | Flake | |  |
| AH | QUE | Count | | | 153 | | 785 | | 938 |
|  |  | Expected Count | | | 279.3 | | 658.7 | | 938.0 |
|  |  | Standardized Residual | | | -7.6 | | 4.9 | |  |
|  |  | Adjusted Residual | | | **-9.7** | | **9.7** | |  |
|  | QUI | Count | | | 88 | | 339 | | 427 |
|  |  | Expected Count | | | 127.2 | | 299.8 | | 427.0 |
|  |  | Standardized Residual | | | -3.5 | | 2.3 | |  |
|  |  | Adjusted Residual | | | **-4.3** | | **4.3** | |  |
|  | ROB | Count | | | 156 | | 520 | | 676 |
|  |  | Expected Count | | | 201.3 | | 474.7 | | 676.0 |
|  |  | Standardized Residual | | | -3.2 | | 2.1 | |  |
|  |  | Adjusted Residual | | | **-4.0** | | **4.0** | |  |
|  | ROS | Count | | | 179 | | 555 | | 734 |
|  |  | Expected Count | | | 218.6 | | 515.4 | | 734.0 |
|  |  | Standardized Residual | | | -2.7 | | 1.7 | |  |
|  |  | Adjusted Residual | | | **-3.4** | | **3.4** | |  |
|  | SAM | Count | | | 176 | | 431 | | 607 |
|  |  | Expected Count | | | 180.8 | | 426.2 | | 607.0 |
|  |  | Standardized Residual | | | -.4 | | .2 | |  |
|  |  | Adjusted Residual | | | -.4 | | .4 | |  |
|  | SAR | Count | | | 137 | | 274 | | 411 |
|  |  | Expected Count | | | 122.4 | | 288.6 | | 411.0 |
|  |  | Standardized Residual | | | 1.3 | | -.9 | |  |
|  |  | Adjusted Residual | | | 1.6 | | -1.6 | |  |
|  | THA | Count | | | 454 | | 890 | | 1344 |
|  |  | Expected Count | | | 400.2 | | 943.8 | | 1344.0 |
|  |  | Standardized Residual | | | 2.7 | | -1.8 | |  |
|  |  | Adjusted Residual | | | **3.6** | | **-3.6** | |  |
|  | THE | Count | | | 790 | | 1236 | | 2026 |
|  |  | Expected Count | | | 603.3 | | 1422.7 | | 2026.0 |
|  |  | Standardized Residual | | | 7.6 | | -4.9 | |  |
|  |  | Adjusted Residual | | | **10.7** | | **-10.7** | |  |
| Total | | Count | | | 2133 | | 5030 | | 7163 |
|  |  | Expected Count | | | 2133.0 | | 5030.0 | | 7163.0 |
| **Chi-Square Tests** | | | | | | | |  |  |
|  | | | Value | df | | Asymptotic Significance (2-sided) | |  |  |
| Pearson Chi-Square | | | 218.466^a^ | 7 | | .000 | |  |  |
| Likelihood Ratio | | | 226.447 | 7 | | .000 | |  |  |
| N of Valid Cases | | | 7163 |  | |  | |  |  |
| a. 0 cells (0.0%) have expected count less than 5. The minimum expected count is 122.39. | | | | | | | |  |  |

| **Symmetric Measures** | | | |
| --- | --- | --- | --- |
|  | | Value | Approximate Significance |
| Nominal by Nominal | Phi | .175 | .000 |
|  | Cramer's V | .175 | .000 |
| N of Valid Cases | | 7163 |  |

**S6 Table.** Diachronic distribution (in %) of blank types by *Abtrag* in the HP of Sibudu.

| **Layer** | **Abtrag** | **Flake** | **Blade** | **Bladelet** | **Point** | **TOTAL n** |
| --- | --- | --- | --- | --- | --- | --- |
| QUENTIN | 1 | 81.9 | 15.2 | 0.6 | 2.3 | 342 |
| QUENTIN | 2 | 84.7 | 11.9 | 1.1 | 2.3 | 261 |
| QUENTIN | 3 | 76.6 | 18.3 | 2.9 | 2.3 | 175 |
| QUENTIN | 4 | 78.5 | 19.8 | 0.6 | 1.1 | 177 |
| QUINCY | 1 | 75.6 | 21.0 | 1.1 | 2.3 | 262 |
| QUINCY | 2 | 78.8 | 17.9 | 0.6 | 2.8 | 179 |
| ROBERT | 1 | 74.7 | 20.2 | 3.0 | 2.2 | 501 |
| ROBERT | 2 | 68.2 | 25.2 | 2.8 | 3.7 | 214 |
| ROSA | 1 | 73.4 | 21.7 | 2.8 | 2.2 | 323 |
| ROSA | 2 | 70.4 | 23.1 | 4.1 | 2.4 | 169 |
| ROSA | 3 | 69.5 | 24.8 | 3.2 | 2.5 | 282 |
| SAMAN | 1 | 65.5 | 30.6 | 3.2 | 0.6 | 310 |
| SAMAN | 2 | 71.2 | 25.3 | 1.6 | 1.9 | 316 |
| SARAH | 1 | 69.1 | 27.6 | 2.4 | 0.8 | 246 |
| SARAH | 2 | 56.7 | 38.2 | 3.9 | 1.1 | 178 |
| THABO | 1 | 63.7 | 29.1 | 4.8 | 2.4 | 251 |
| THABO | 2 | 59.3 | 37.5 | 2.5 | 0.7 | 275 |
| THABO | 3 | 63.5 | 29.6 | 4.9 | 2.0 | 307 |
| THABO | 4 | 62.9 | 32.7 | 3.1 | 1.3 | 318 |
| THABO | 5 | 64.5 | 30.5 | 3.8 | 1.1 | 262 |
| THEODORA | 1 | 60.3 | 36.9 | 2.1 | 0.8 | 521 |
| THEODORA | 2 | 55.9 | 39.6 | 3.2 | 1.3 | 379 |
| THEODORA | 3 | 56.9 | 37.8 | 3.7 | 1.6 | 376 |
| THEODORA | 4 | 58.4 | 38.2 | 2.7 | 0.8 | 377 |
| THEODORA | 5 | 59.9 | 35.1 | 3.3 | 1.7 | 461 |

**S7 Table.** Summary statistics of ANOVA test for blade width at Sibudu. 4= THE&THA; 3=SAM&SAR; 2=ROB&ROS; 1=QUE&QUI. Significant post-hoc differences are marked in boldface. See SOM Figure 3 for illustration of these results.

| **Descriptives** | | | | | | | |
| --- | --- | --- | --- | --- | --- | --- | --- |
| BladeWidth | | | | | | | |
|  | N | Mean | Std. Deviation | Std. Error | 95% Confidence Interval for Mean | |  |
|  |  |  |  |  | Lower Bound | Upper Bound |  |
| 1 | 215 | 17.56 | 3.672 | .250 | 17.07 | 18.06 |  |
| 2 | 305 | 17.77 | 4.134 | .237 | 17.31 | 18.24 |  |
| 3 | 283 | 17.95 | 3.463 | .206 | 17.55 | 18.36 |  |
| 4 | 1162 | 18.54 | 4.039 | .118 | 18.31 | 18.78 |  |
| Total | 1965 | 18.23 | 3.953 | .089 | 18.06 | 18.41 |  |

| **Test of Homogeneity of Variances** | | | |
| --- | --- | --- | --- |
| BladeWidth | | | |
| Levene Statistic | df1 | df2 | Sig. |
| 3.675 | 3 | 1961 | **.012** |

| **ANOVA** | | | | | |
| --- | --- | --- | --- | --- | --- |
| BladeWidth | | | | | |
|  | Sum of Squares | df | Mean Square | F | Sig. |
| Between Groups | 296.407 | 3 | 98.802 | 6.373 | **.000** |
| Within Groups | 30399.773 | 1961 | 15.502 |  |  |
| Total | 30696.180 | 1964 |  |  |  |

| **Multiple Comparisons** | | | | | | | |
| --- | --- | --- | --- | --- | --- | --- | --- |
| Dependent Variable: BladeWidth | | | | | | | |
|  | (I) AH | (J) AH | Mean Difference (I-J) | Std. Error | Sig. | 95% Confidence Interval | |
|  |  |  |  |  |  | Lower Bound | Upper Bound |
| Games-Howell | 1 | 2 | -.211 | .345 | .928 | -1.10 | .68 |
|  |  | 3 | -.388 | .324 | .630 | -1.22 | .45 |
|  |  | 4 | -.982^*^ | .277 | **.003** | -1.70 | -.27 |
|  | 2 | 1 | .211 | .345 | .928 | -.68 | 1.10 |
|  |  | 3 | -.177 | .314 | .943 | -.98 | .63 |
|  |  | 4 | -.771^*^ | .265 | **.020** | -1.45 | -.09 |
|  | 3 | 1 | .388 | .324 | .630 | -.45 | 1.22 |
|  |  | 2 | .177 | .314 | .943 | -.63 | .98 |
|  |  | 4 | -.594 | .238 | .061 | -1.21 | .02 |
|  | 4 | 1 | .982^*^ | .277 | **.003** | .27 | 1.70 |
|  |  | 2 | .771^*^ | .265 | **.020** | .09 | 1.45 |
|  |  | 3 | .594 | .238 | .061 | -.02 | 1.21 |

**S8 Table.** Diachronic distribution of Conard et al. (2004) core type (n) by layer in the HP of Sibudu. Numbers of cores in parentheses among platform variants refer to typical HP cores.

| **Layer** | **Parallel** | **Platform** | **Inclined** | **Bipolar** | **Initial/Other** | **IBR** | **TOTAL** |
| --- | --- | --- | --- | --- | --- | --- | --- |
| QUENTIN | 3 | 3 (2) |  | 10 | 1 | 1 | 18 |
| QUINCY | 1 | 3 (1) |  | 3 |  |  | 7 |
| ROBERT | 1 | 4 (1) |  | 10 |  | 1 | 16 |
| ROSA |  | 4 (4) |  | 6 |  |  | 10 |
| SAMAN | 2 | 4 (2) |  | 6 |  |  | 12 |
| SARAH | 1 | 3 (1) |  | 1 | 1 |  | 6 |
| THABO | 1 | 13 (9) | 1 | 8 |  |  | 23 |
| THEODORA | 4 | 24 (11) |  | 11 | 1 | 3 | 43 |
| Total | 13 | 58 | 1 | 55 | 3 | 5 | 135 |

**S9 Table.** Diachronic distribution of classic tool types (in %) by *Abtrag* in the HP of Sibudu. Note the small sample size (n) for some of the *Abträge*.

| **Layer** | **Abtrag** | **Backed piece** | **Bifacial point** | **Notch & Denticulate** | **Splintered piece** | **Unifacial point** | **Lateral retouch** | **Minimal retouch** | **Scraper** | **TOTAL n** |
| --- | --- | --- | --- | --- | --- | --- | --- | --- | --- | --- |
| QUENTIN | 1 | 25.0 | 37.5 | 0.0 | 25.0 | 0.0 | 12.5 | 0.0 | 0.0 | 8 |
| QUENTIN | 2 | 22.2 | 22.2 | 22.2 | 11.1 | 11.1 | 0.0 | 0.0 | 11.1 | 9 |
| QUENTIN | 3 | 75.0 | 25.0 | 0.0 | 0.0 | 0.0 | 0.0 | 0.0 | 0.0 | 8 |
| QUENTIN | 4 | 50.0 | 21.4 | 0.0 | 14.3 | 0.0 | 0.0 | 0.0 | 14.3 | 14 |
| QUINCY | 1 | 45.5 | 9.1 | 0.0 | 18.2 | 0.0 | 18.2 | 9.1 | 0.0 | 11 |
| QUINCY | 2 | 20.0 | 13.3 | 33.3 | 0.0 | 13.3 | 6.7 | 6.7 | 6.7 | 15 |
| ROBERT | 1 | 39.5 | 23.3 | 7.0 | 4.7 | 4.7 | 7.0 | 11.6 | 2.3 | 43 |
| ROBERT | 2 | 55.6 | 22.2 | 5.6 | 5.6 | 0.0 | 5.6 | 5.6 | 0.0 | 18 |
| ROSA | 1 | 55.0 | 15.0 | 10.0 | 0.0 | 0.0 | 5.0 | 15.0 | 0.0 | 20 |
| ROSA | 2 | 57.1 | 0.0 | 21.4 | 14.3 | 0.0 | 7.1 | 0.0 | 0.0 | 14 |
| ROSA | 3 | 65.4 | 7.7 | 15.4 | 7.7 | 0.0 | 0.0 | 3.8 | 0.0 | 26 |
| SAMAN | 1 | 90.9 | 0.0 | 4.5 | 0.0 | 0.0 | 0.0 | 0.0 | 4.5 | 22 |
| SAMAN | 2 | 66.7 | 5.6 | 11.1 | 5.6 | 0.0 | 5.6 | 5.6 | 0.0 | 18 |
| SARAH | 1 | 36.8 | 5.3 | 21.1 | 10.5 | 0.0 | 10.5 | 15.8 | 0.0 | 19 |
| SARAH | 2 | 50.0 | 0.0 | 0.0 | 16.7 | 8.3 | 16.7 | 0.0 | 0.0 | 12 |
| THABO | 1 | 65.0 | 0.0 | 15.0 | 5.0 | 10.0 | 5.0 | 0.0 | 0.0 | 20 |
| THABO | 2 | 68.0 | 0.0 | 12.0 | 8.0 | 4.0 | 0.0 | 4.0 | 4.0 | 25 |
| THABO | 3 | 84.6 | 0.0 | 3.8 | 0.0 | 0.0 | 3.8 | 7.7 | 0.0 | 26 |
| THABO | 4 | 68.4 | 0.0 | 10.5 | 15.8 | 0.0 | 0.0 | 5.3 | 0.0 | 19 |
| THABO | 5 | 60.0 | 0.0 | 10.0 | 0.0 | 10.0 | 10.0 | 10.0 | 0.0 | 10 |
| THEODORA | 1 | 60.0 | 0.0 | 8.0 | 16.0 | 0.0 | 8.0 | 4.0 | 4.0 | 25 |
| THEODORA | 2 | 55.9 | 0.0 | 14.7 | 5.9 | 0.0 | 11.8 | 5.9 | 0.0 | 34 |
| THEODORA | 3 | 81.8 | 0.0 | 9.1 | 0.0 | 0.0 | 0.0 | 0.0 | 9.1 | 22 |
| THEODORA | 4 | 57.9 | 0.0 | 15.8 | 5.3 | 0.0 | 0.0 | 21.1 | 0.0 | 19 |
| THEODORA | 5 | 60.0 | 0.0 | 8.0 | 4.0 | 0.0 | 4.0 | 16.0 | 8.0 | 25 |

**S10 Table.** Results of Chi-Square test for tool type, comparing the numerical distribution of backed and bifacial pieces by 2-layer combinations (due to sample size limitations). Note the distribution of adjusted residuals throughout the sequence (e.g. for backed pieces from highly negative in QUE-QUI to highly positive in THE-THA). Significant associations (adjusted residuals >1.96, Agresti 2007; Sharpe 2015) marked in bold, found for 3 out of 4 HP layer combinations.

| **AH * Tool Crosstabulation** | | | | | |
| --- | --- | --- | --- | --- | --- |
|  | | | Tool | | Total |
|  |  |  | Back Piece | Bif Point |  |
| AH | QUE&QUI | Count | 25 | 13 | 38 |
|  |  | Expected Count | 33.9 | 4.1 | 38.0 |
|  |  | % within AH | 65.8% | 34.2% | 100.0% |
|  |  | Standardized Residual | -1.5 | 4.4 |  |
|  |  | Adjusted Residual | **-5.0** | **5.0** |  |
|  | ROB&ROS | Count | 63 | 19 | 82 |
|  |  | Expected Count | 73.2 | 8.8 | 82.0 |
|  |  | % within AH | 76.8% | 23.2% | 100.0% |
|  |  | Standardized Residual | -1.2 | 3.4 |  |
|  |  | Adjusted Residual | **-4.2** | **4.2** |  |
|  | SAM&SAR | Count | 45 | 2 | 47 |
|  |  | Expected Count | 42.0 | 5.0 | 47.0 |
|  |  | % within AH | 95.7% | 4.3% | 100.0% |
|  |  | Standardized Residual | .5 | -1.4 |  |
|  |  | Adjusted Residual | 1.6 | -1.6 |  |
|  | THA&THE | Count | 150 | 0 | 150 |
|  |  | Expected Count | 133.9 | 16.1 | 150.0 |
|  |  | % within AH | 100.0% | 0.0% | 100.0% |
|  |  | Standardized Residual | 1.4 | -4.0 |  |
|  |  | Adjusted Residual | **5.8** | **-5.8** |  |
| Total | | Count | 283 | 34 | 317 |
|  |  | Expected Count | 283.0 | 34.0 | 317.0 |
|  |  | % within AH | 89.3% | 10.7% | 100.0% |

| **Chi-Square Tests** | | | |
| --- | --- | --- | --- |
|  | Value | df | Asymptotic Significance (2-sided) |
| Pearson Chi-Square | 55.228^a^ | 3 | .000 |
| Likelihood Ratio | 61.884 | 3 | .000 |
| N of Valid Cases | 317 |  |  |
| a. 1 cells (12.5%) have expected count less than 5. The minimum expected count is 4.08. | | | |

| **Symmetric Measures** | | | |
| --- | --- | --- | --- |
|  | | Value | Approximate Significance |
| Nominal by Nominal | Phi | .417 | .000 |
|  | Cramer's V | .417 | .000 |
| N of Valid Cases | | 317 |  |

**S11 Table**. Summary tables of the *k*-means cluster analysis finding three clusters: THEODORA-SARAH (Cluster 1); SAMAN-ROSA (Cluster 2) and ROBERT-QUENTIN (Cluster 3).

| **Cluster Membership** | | | |
| --- | --- | --- | --- |
| Case Number | Layer | Cluster | Distance |
| 1 | THE | 1 | 8.234 |
| 2 | THA | 1 | 15.646 |
| 3 | SAR | 1 | 20.530 |
| 4 | SAM | 2 | 11.842 |
| 5 | ROS | 2 | 11.842 |
| 6 | ROB | 3 | 9.690 |
| 7 | QUI | 3 | 14.589 |
| 8 | QUE | 3 | 14.508 |

| **Initial Cluster Centers** | | | |
| --- | --- | --- | --- |
|  | Cluster | | |
|  | 1 | 2 | 3 |
| Dolerite | 74.60 | 71.30 | 59.90 |
| Sandstone | 15.30 | 22.50 | 30.20 |
| Quartz | 1.10 | 1.80 | 2.70 |
| Tool | 5.80 | 6.10 | 3.80 |
| Blade | 37.40 | 27.90 | 15.80 |
| Flake | 58.50 | 68.40 | 81.00 |
| BifacialPoint | .00 | 2.50 | 25.60 |
| BackedPiece | 60.50 | 80.00 | 43.60 |
| Bladelet | 2.90 | 2.40 | 1.10 |
| BladeLength | 46.20 | 40.50 | 41.20 |
| BladeWidth | 18.60 | 17.50 | 17.50 |
| ShatteredBulb | 4.50 | 3.20 | 13.40 |
| Lipping | 30.30 | 22.30 | 11.00 |
| EPA | 80.20 | 81.20 | 83.70 |
| AbsentBulb | 59.30 | 51.10 | 34.10 |

| **Final Cluster Centers** | | | |
| --- | --- | --- | --- |
|  | Cluster | | |
|  | 1 | 2 | 3 |
| Dolerite | 72.03 | 71.80 | 61.30 |
| Sandstone | 16.80 | 21.60 | 29.07 |
| Quartz | 1.13 | 2.35 | 3.33 |
| Tool | 6.97 | 6.80 | 5.87 |
| Blade | 33.80 | 25.45 | 19.17 |
| Flake | 61.73 | 69.85 | 76.77 |
| BifacialPoint | .97 | 5.35 | 20.03 |
| BackedPiece | 55.83 | 69.50 | 39.57 |
| Bladelet | 3.27 | 2.80 | 1.63 |
| BladeLength | 45.30 | 41.85 | 42.70 |
| BladeWidth | 18.50 | 17.40 | 17.70 |
| ShatteredBulb | 5.33 | 4.70 | 11.10 |
| Lipping | 27.83 | 22.90 | 14.17 |
| EPA | 80.70 | 81.20 | 83.73 |
| AbsentBulb | 57.43 | 48.40 | 44.50 |

| **Distances between Final Cluster Centers** | | | |
| --- | --- | --- | --- |
| Cluster | 1 | 2 | 3 |
| 1 |  | 22.048 | 41.792 |
| 2 | 22.048 |  | 38.830 |
| 3 | 41.792 | 38.830 |  |

**S12 Table**. General information and loading patterns of the PCA by individual components for all HP assemblages at Sibudu.

| **Communalities** | | |
| --- | --- | --- |
|  | Initial | Extraction |
| Dolerite | 1.000 | .946 |
| Sandstone | 1.000 | .956 |
| Quartz | 1.000 | .867 |
| Tool | 1.000 | .792 |
| Flake | 1.000 | .965 |
| Blade | 1.000 | .950 |
| Bladelet | 1.000 | .963 |
| BackedPiece | 1.000 | .945 |
| BifacialPoint | 1.000 | .936 |
| BladeLength | 1.000 | .909 |
| BladeWidth | 1.000 | .755 |
| ShatteredBulb | 1.000 | .803 |
| Lipping | 1.000 | .905 |
| EPA | 1.000 | .964 |
| AbsentBulb | 1.000 | .871 |
| Extraction Method: Principal Component Analysis. | | |

| **Component Matrix^a^** | | | |
| --- | --- | --- | --- |
|  | Component | | |
|  | 1 | 2 | 3 |
| Dolerite | .930 | -.286 | .012 |
| Sandstone | -.971 | .065 | -.094 |
| Quartz | -.750 | .037 | .551 |
| Tool | .428 | .400 | .670 |
| Flake | -.974 | -.111 | -.073 |
| Blade | .968 | .108 | -.048 |
| Bladelet | .746 | -.003 | .637 |
| BackedPiece | .516 | -.784 | .254 |
| BifacialPoint | -.935 | .153 | .197 |
| BladeLength | .552 | .713 | -.310 |
| BladeWidth | .657 | .563 | .081 |
| ShatteredBulb | -.802 | .240 | .321 |
| Lipping | .937 | -.165 | .016 |
| EPA | -.946 | .262 | .030 |
| AbsentBulb | .829 | .426 | -.041 |
| Extraction Method: Principal Component Analysis. | | | |
| a. 3 components extracted. | | | |

| **Total Variance Explained** | | | | | | |
| --- | --- | --- | --- | --- | --- | --- |
| Component | Initial Eigenvalues | | | Extraction Sums of Squared Loadings | | |
|  | Total | % of Variance | Cumulative % | Total | % of Variance | Cumulative % |
| 1 | 9.972 | 66.479 | 66.479 | 9.972 | 66.479 | 66.479 |
| 2 | 2.069 | 13.793 | 80.272 | 2.069 | 13.793 | 80.272 |
| 3 | 1.486 | 9.907 | 90.179 | 1.486 | 9.907 | 90.179 |
| 4 | .793 | 5.284 | 95.462 |  |  |  |
| 5 | .354 | 2.357 | 97.819 |  |  |  |
| 6 | .227 | 1.515 | 99.334 |  |  |  |
| 7 | .100 | .666 | 100.000 |  |  |  |
| 8 | 1.387E-15 | 9.249E-15 | 100.000 |  |  |  |
| 9 | 6.521E-16 | 4.348E-15 | 100.000 |  |  |  |
| 10 | 2.878E-16 | 1.919E-15 | 100.000 |  |  |  |
| 11 | 9.693E-17 | 6.462E-16 | 100.000 |  |  |  |
| 12 | -6.966E-17 | -4.644E-16 | 100.000 |  |  |  |
| 13 | -1.275E-16 | -8.502E-16 | 100.000 |  |  |  |
| 14 | -2.715E-16 | -1.810E-15 | 100.000 |  |  |  |
| 15 | -6.536E-16 | -4.357E-15 | 100.000 |  |  |  |
