# Supplementary material for: Regional patterns of diachronic technological change in the Howiesons Poort of southern Africa
Source: PLoS One. 2020 Sep 17;15(9):e0239195. doi: 10.1371/journal.pone.0239195 (PMC7498030; doi:10.1371/journal.pone.0239195)
Supplement: S3 File — (DOCX) [file pone.0239195.s003.docx]

**SUPPLEMENTARY INFORMATION S1-S8 TEXT**

*METHODS*

**S1 Text.** Attribute list and attribute expressions recorded for the HP at Sibudu.

I. Main information (taken for all lithic objects)

**1. Find ID** [*Number/Text*]

**2. Stratigraphic unit** [Layer name]

**3**. **Main category** [core, tool, blank, angular debris, small debitage, stone, surface]

**4**. **Raw material category** [dolerite, hornfels, quartz, quartzite, jasper, sandstone, other]

**5.** **Stage of** **reduction sequence** [1 Cobble of raw material, 2 First reduction of cobble, 3 preparation of cobble, 4 core, 5 core rejuvenation, 6 blank production, 7 angular debris, 8 utilization of blank, 9 retouch of blank, 10 tool, 11 retouch of tool, 12 broken tool]

**6. Weight (g)** [*Number*]

**7. Dorsal cortex cover** [*in %*]

II. Metrics

**8**. **Maximum dimension (mm)** [*Number*]

**9**. **Length (mm)** [*Number*]

**10**. **Width (mm)** [*Number*]

**11**. **Thickness (mm)** [*Number*]

**12.** **Exterior platform angle (degrees)** [*Number*]

IIIa. Blanks

**13**. **Blank type** [flake, blade, point, bladelet]

**14**. **Completeness** [complete, proximal, distal, medial, indeterminate]

**15.** **Completeness (%)** [*Numbe*r]

**16.** **Bulb** [not preserved, absent, poor-developed, developed, well-developed, indeterminate]

**17**. **Platform type** [not preserved, punctiform, linear, cortical, plain, dihedral, coarse facetted, medium facetted, fine facetted, crushed/shattered, indeterminate]

**18. Platform completeness (%)** [*Number*]

**19. Platform width (mm)** [*Number*]

**20. Platform thickness (mm)** [*Number*]

**21**. **# Dorsal Negatives** [*Number*]

**22. Orientation Dorsal Negatives** [orthogonal, centripetal, opposed, parallel, irregular, indeterminate]

**23. Core reduction system** [Discoid, Laminar, Levallois, Informal/Indeterminate, Bipolar]

**24. Product core reduction system** [Dos limité (D), Debordant (D), Central flake (D), Invasive flake (D), Eclat debordant (La), Unidirectional (La), Bidirectional (La), Orthogonal (La), Plunging (La), Cortical (La), Flake/Point (Le), Maintenance (Le), Preparation (Le), Broken (I), Cortical (I), Flake (I)]

**25**. **Contact point** [*Checkbox*]

**26. Lip** [*Checkbox*]

**27. Hertzian cone** [*Checkbox*]

**28. Shattered bulb** [*Checkbox*]

IV. Tools (in addition to blank attributes)

**29**. **Tool category** [Formal, Informal, Groundstone tool]

**30. Techno-functional tool class** [Tongati, Ndwedwe, ACT, NBT, ...]

**31**. **Tool type** [backed piece (segment), backed piece (trapezoid), backed piece (other), scraper, side scraper, transverse scraper, end scraper, denticulate, notch, strangulated piece, burin, hammerstone, anvil, unifacial point, bifacial point lateral retouch, end retouch, minimal retouch on flake, splintered piece, minimal retouch on blade, minimal retouch on point, indeterminate]

**32. Retouch side** [ventral, dorsal, bilateral, indeterminate]

**33. Retouch location** [proximal, medial, distal, proximal+medial, proximal+distal, medial+distal, all edges, indeterminate]

**34.** **Retouch Description** [fine, coarse, notched, steep, backed, surface, stepped, irregular]

**35. Preservation** [complete, nearly complete, fragment]

V. Cores

**36.** **Category** [Bipolar, Initial, Inclined, Parallel, Platform, Multidirectional, Other, Indeterminate Broken]

**37. HP core?** [*Checkbox*]

**38.** **Product** [Flake, Blade, Point, Bladelet]

**39**. **Direction of removals** [bidirectional, centripetal, preferential (=1), unidirectional (>1), unifacial, bifacial, adjacent, irregular]

**40**. **# Striking platforms** [*Number*]

**41. Orientation striking platforms (if >1)** [not preserved, adjacent, opposed, alternating, irregular, indeterminate]

**42**. **# Removal surfaces** [*Number*]

**43**. **Technique** [bipolar, bipolar (?), handheld, indeterminate]

*DISCUSSION*

**S2 Text.** Summary of statistical analyses for the HP sequence at Diepkloof. Raw data on lithic attributes from Porraz et al. (2013).

**a) Kendall’s tau correlations summary (individual SPSS output below)**

- Unidirectional trends are found for: %quartz, %quartzite / %backedpiece, %splinteredpiece
  - INCREASE in %backedpiece and %quartz throughout HP sequence (significant)
  - DECREASE in %quartzite and %splinteredpiece throughout HP sequence (significant)
- No unidirectional trends are found for: %silcrete %blades, %blades&bladelets
- Also: No significant correlation between %blades&bladelets and %backedpieces (t=-0.149; p=0.687)
- But: Significant correlation between %silcrete and %backedpieces (t=-0.363; p=0.016)

**Nonparametric Correlations (Layer and %silcrete)**

| **Correlations** | | | | |
| --- | --- | --- | --- | --- |
|  | | | Layer | Silcrete |
| Kendall's tau_b | Layer | Correlation Coefficient | 1.000 | -.203 |
|  |  | Sig. (2-tailed) | . | .165 |
|  |  | N | 24 | 24 |
|  | Silcrete | Correlation Coefficient | -.203 | 1.000 |
|  |  | Sig. (2-tailed) | .165 | . |
|  |  | N | 24 | 24 |

**Nonparametric Correlations (Layer and %quartz)**

| **Correlations** | | | | |
| --- | --- | --- | --- | --- |
|  | | | Layer | Quartz |
| Kendall's tau_b | Layer | Correlation Coefficient | 1.000 | **.464^**^** |
|  |  | Sig. (2-tailed) | . | .001 |
|  |  | N | 24 | 24 |
|  | Quartz | Correlation Coefficient | .464^**^ | 1.000 |
|  |  | Sig. (2-tailed) | .001 | . |
|  |  | N | 24 | 24 |
| **. Correlation is significant at the 0.01 level (2-tailed). | | | | |

**Nonparametric Correlations (Layer and %quartzite)**

|  | | | | |
| --- | --- | --- | --- | --- |
|  | | | Layer | Quartzite |
| Kendall's tau_b | Layer | Correlation Coefficient | 1.000 | **-.642^**^** |
|  |  | Sig. (2-tailed) | . | .000 |
|  |  | N | 24 | 24 |
|  | Quartzite | Correlation Coefficient | -.642^**^ | 1.000 |
|  |  | Sig. (2-tailed) | .000 | . |
|  |  | N | 24 | 24 |
| **. Correlation is significant at the 0.01 level (2-tailed). | | | | |

**Nonparametric Correlations (Layer and %backed piece)**

|  | | | | |
| --- | --- | --- | --- | --- |
|  | | | Layer | BackedPiece |
| Kendall's tau_b | Layer | Correlation Coefficient | 1.000 | **.544^**^** |
|  |  | Sig. (2-tailed) | . | .000 |
|  |  | N | 24 | 24 |
|  | BackedPiece | Correlation Coefficient | .544^**^ | 1.000 |
|  |  | Sig. (2-tailed) | .000 | . |
|  |  | N | 24 | 24 |
| **. Correlation is significant at the 0.01 level (2-tailed). | | | | |

**Nonparametric Correlations (Layer and %splintered piece)**

|  | | | | |
| --- | --- | --- | --- | --- |
|  | | | Layer | SplinteredPiece |
| Kendall's tau_b | Layer | Correlation Coefficient | 1.000 | **-.410^**^** |
|  |  | Sig. (2-tailed) | . | .005 |
|  |  | N | 24 | 24 |
|  | SplinteredPiece | Correlation Coefficient | -.410^**^ | 1.000 |
|  |  | Sig. (2-tailed) | .005 | . |
|  |  | N | 24 | 24 |
| **. Correlation is significant at the 0.01 level (2-tailed). | | | | |

**Nonparametric Correlations (Layer and %blade)**

|  | | | | |
| --- | --- | --- | --- | --- |
|  | | | Layer | Blades |
| Kendall's tau_b | Layer | Correlation Coefficient | 1.000 | .200 |
|  |  | Sig. (2-tailed) | . | .573 |
|  |  | N | 24 | 6 |
|  | Blades | Correlation Coefficient | .200 | 1.000 |
|  |  | Sig. (2-tailed) | .573 | . |
|  |  | N | 6 | 6 |

**Nonparametric Correlations (Layer and %blade&bladelets)**

|  | | | | |
| --- | --- | --- | --- | --- |
|  | | | Layer | BladesAndBladelets |
| Kendall's tau_b | Layer | Correlation Coefficient | 1.000 | .067 |
|  |  | Sig. (2-tailed) | . | .851 |
|  |  | N | 24 | 6 |
|  | BladesAndBladelets | Correlation Coefficient | .067 | 1.000 |
|  |  | Sig. (2-tailed) | .851 | . |
|  |  | N | 6 | 6 |

**b) Scatter plots**

**Layer and %Silcrete**

- Non-directional patterns through time with multiple peaks and minima (middle part on average higher)


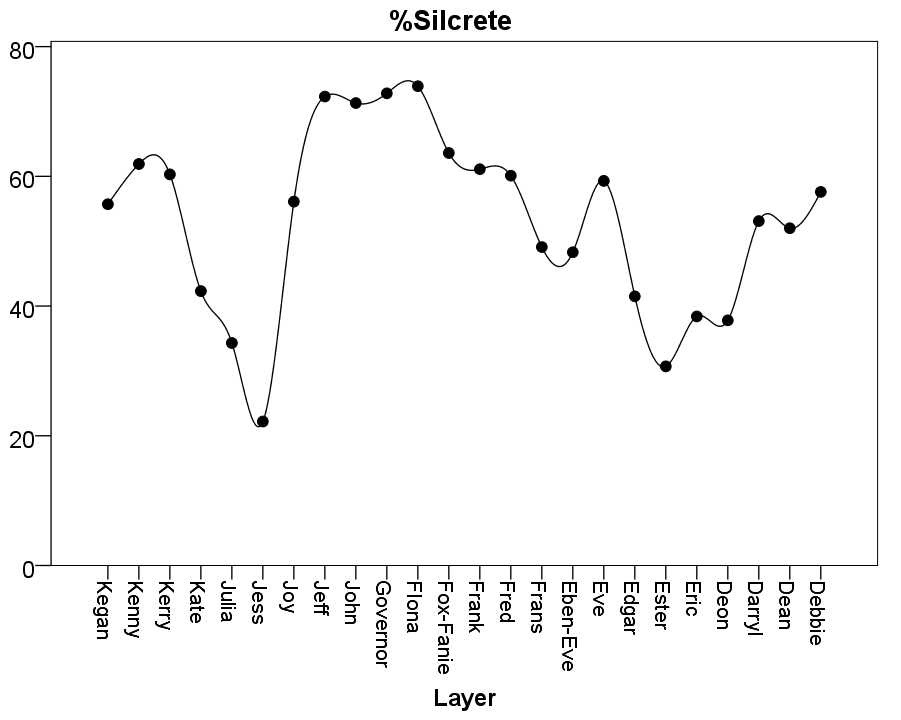


**Layer and %Quartz / %Quartzite**

- Clear and contradictory unidirectional trends (Quartz -> increase; quartzite -> decrease)


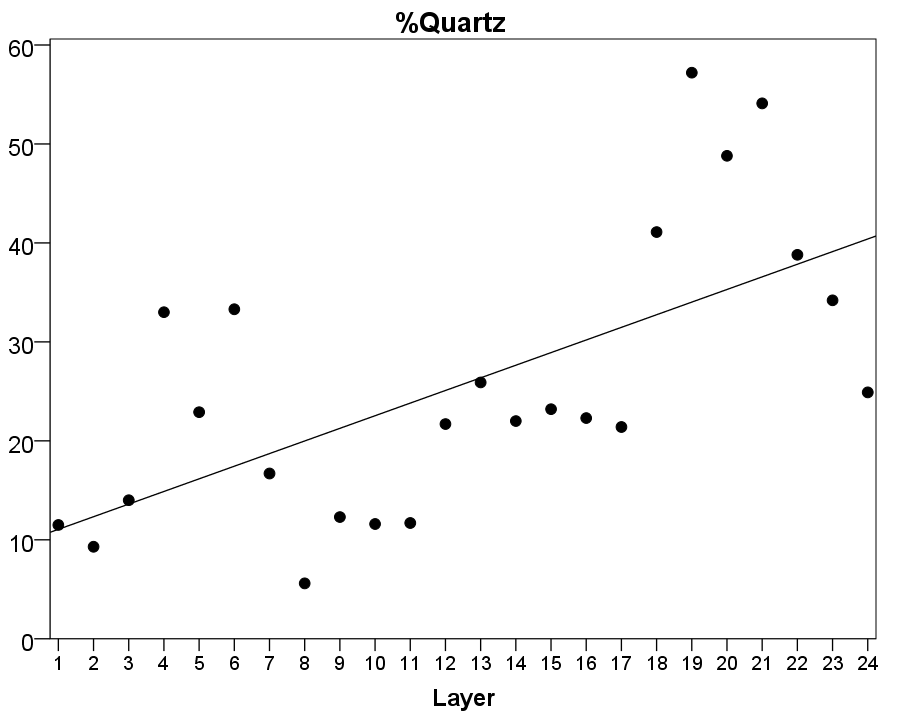


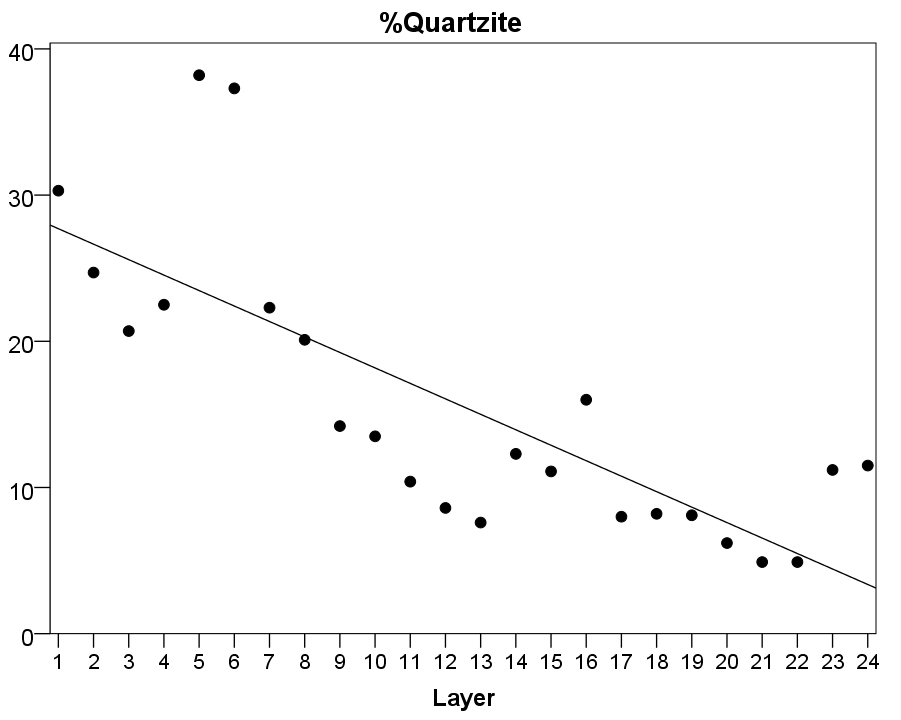


**Layer and %Backed pieces / %splintered pieces**

- Clear and contradictory unidirectional trends (backed piece -> increase; splintered -> decrease)


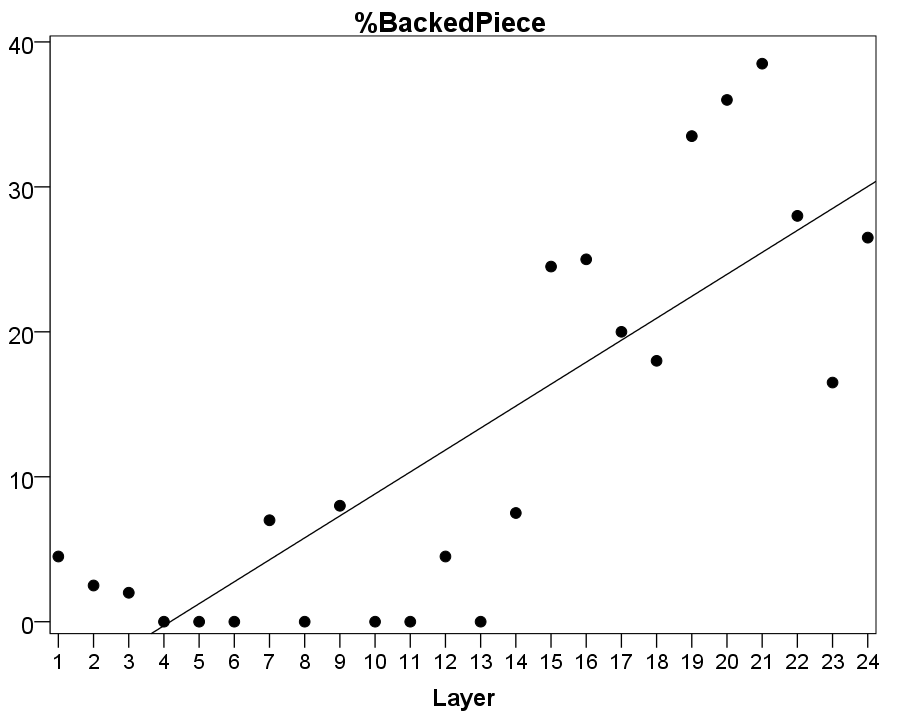


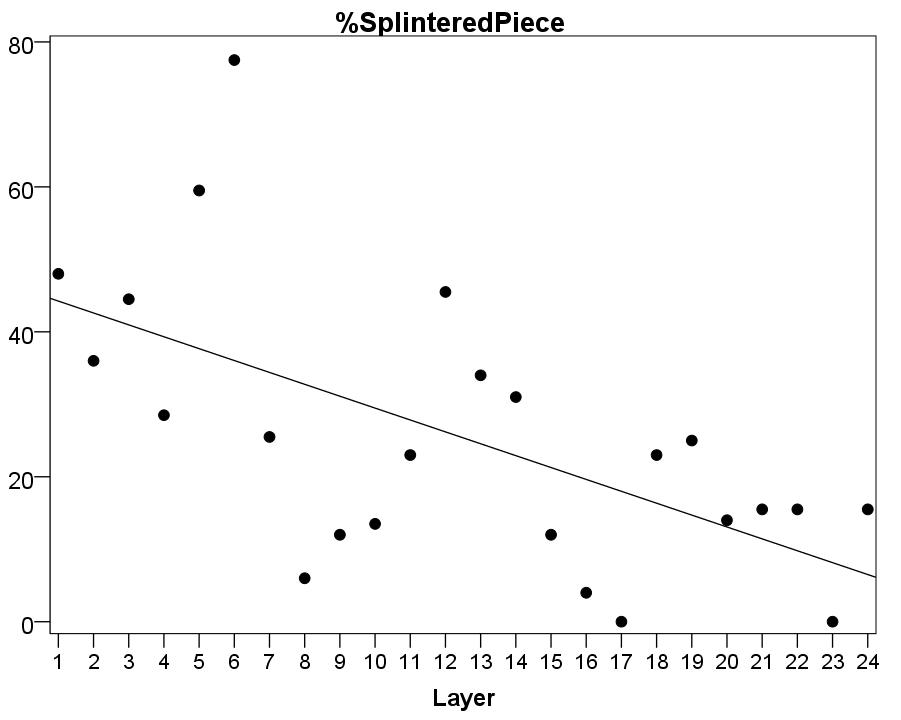


**Layer and %Blade&Bladelet**

- Shows no clear unidirectional trends but rather a curving pattern with at least 1 peak and 1 minimum


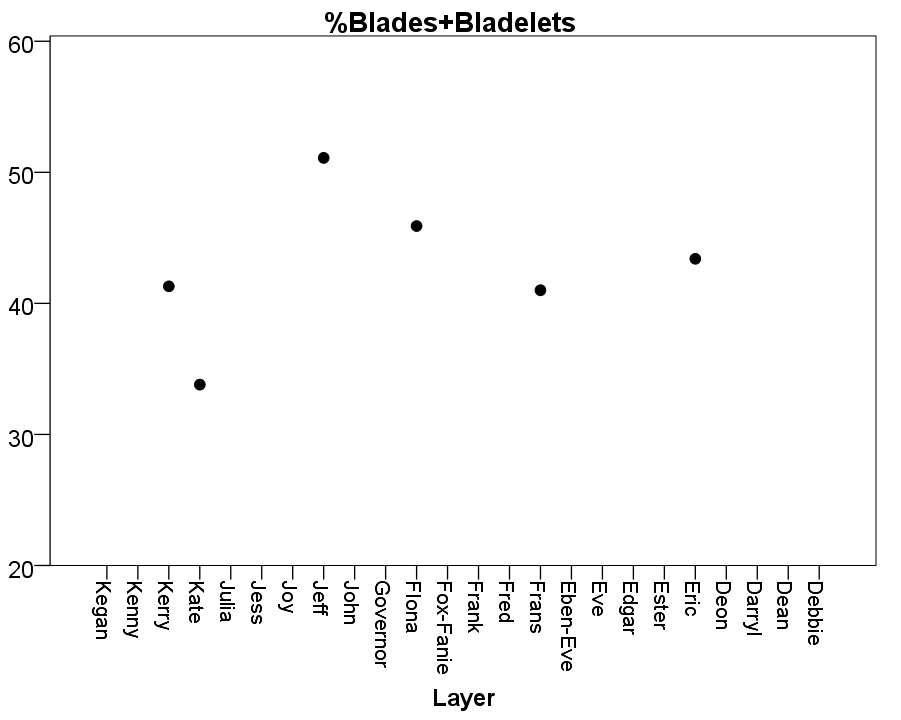


**S3 Text.** Summary of statistical analyses for the HP sequence at Klipdrift. Raw data on lithic attributes from Douze et al. (2018).

**a) Kendall’s tau correlations summary (individual SPSS output below)**

- **No significant unidirectional trends** are found **for any of the studied variables** (RMU, tool types, blank types)!
  - **Mostly curving/cubic patterns** (silcrete, quartz, and elongation>2)
  - But: backed pieces show a general (non-significant) increase throughout the sequence
- Also: No significant correlation between %elongation>2 and %backedpieces (t=-0.295; p=0.189) and between %silcrete and %backedpieces (t=-0.047; p=0.836)

**Nonparametric Correlations (Layer and %silcrete)**

|  | | | | |
| --- | --- | --- | --- | --- |
|  | | | Silcrete | Layer |
| Kendall's tau_b | Silcrete | Correlation Coefficient | 1.000 | .030 |
|  |  | Sig. (2-tailed) | . | .891 |
|  |  | N | 12 | 12 |
|  | Layer | Correlation Coefficient | .030 | 1.000 |
|  |  | Sig. (2-tailed) | .891 | . |
|  |  | N | 12 | 12 |

**Nonparametric Correlations (Layer and %quartz)**

|  | | | | |
| --- | --- | --- | --- | --- |
|  | | | Layer | Quartz |
| Kendall's tau_b | Layer | Correlation Coefficient | 1.000 | .091 |
|  |  | Sig. (2-tailed) | . | .681 |
|  |  | N | 12 | 12 |
|  | Quartz | Correlation Coefficient | .091 | 1.000 |
|  |  | Sig. (2-tailed) | .681 | . |
|  |  | N | 12 | 12 |

**Nonparametric Correlations (Layer and %backed piece)**

|  | | | | |
| --- | --- | --- | --- | --- |
|  | | | Layer | BackedPiece |
| Kendall's tau_b | Layer | Correlation Coefficient | 1.000 | .357 |
|  |  | Sig. (2-tailed) | . | .112 |
|  |  | N | 12 | 12 |
|  | BackedPiece | Correlation Coefficient | .357 | 1.000 |
|  |  | Sig. (2-tailed) | .112 | . |
|  |  | N | 12 | 12 |

**Nonparametric Correlations (Layer and %notches)**

|  | | | | |
| --- | --- | --- | --- | --- |
|  | | | Layer | Notch |
| Kendall's tau_b | Layer | Correlation Coefficient | 1.000 | .016 |
|  |  | Sig. (2-tailed) | . | .944 |
|  |  | N | 12 | 12 |
|  | Notch | Correlation Coefficient | .016 | 1.000 |
|  |  | Sig. (2-tailed) | .944 | . |
|  |  | N | 12 | 12 |

**Nonparametric Correlations (Layer and %Elongation >2:1)**

|  | | | | |
| --- | --- | --- | --- | --- |
|  | | | Layer | Elongate2 |
| Kendall's tau_b | Layer | Correlation Coefficient | 1.000 | -.212 |
|  |  | Sig. (2-tailed) | . | .337 |
|  |  | N | 12 | 12 |
|  | Elongate2 | Correlation Coefficient | -.212 | 1.000 |
|  |  | Sig. (2-tailed) | .337 | . |
|  |  | N | 12 | 12 |

**b) Scatter plots and curve estimations:**

**Layer and %Silcrete**

- Shows a curving (cubic) pattern through time, no consistent increase or decrease

| **Model Summary and Parameter Estimates** | | | | | | | | | |
| --- | --- | --- | --- | --- | --- | --- | --- | --- | --- |
| Dependent Variable: Silcrete | | | | | | | | | |
| Equation | Model Summary | | | | | Parameter Estimates | | | |
|  | R Square | F | df1 | df2 | Sig. | Constant | b1 | b2 | b3 |
| Linear | .000 | .004 | 1 | 10 | .954 | 76.048 | .069 |  |  |
| Cubic | .618 | 4.313 | 3 | 8 | .044 | 99.484 | 23.601 | 4.562 | .238 |
| The independent variable is Layer. | | | | | | | | | |


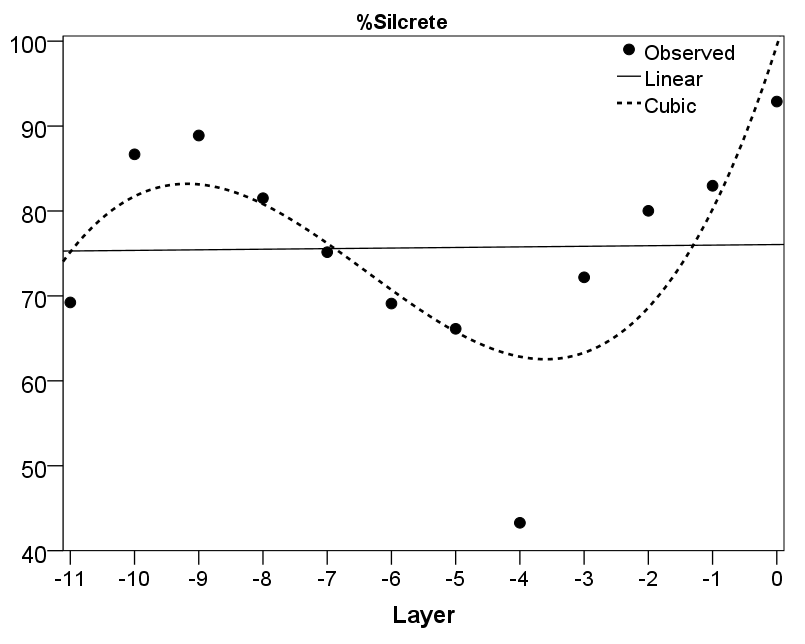


**Layer and %Quartz**

- Shows a unimodal (1 peak) pattern through time, but no consistent increase or decrease


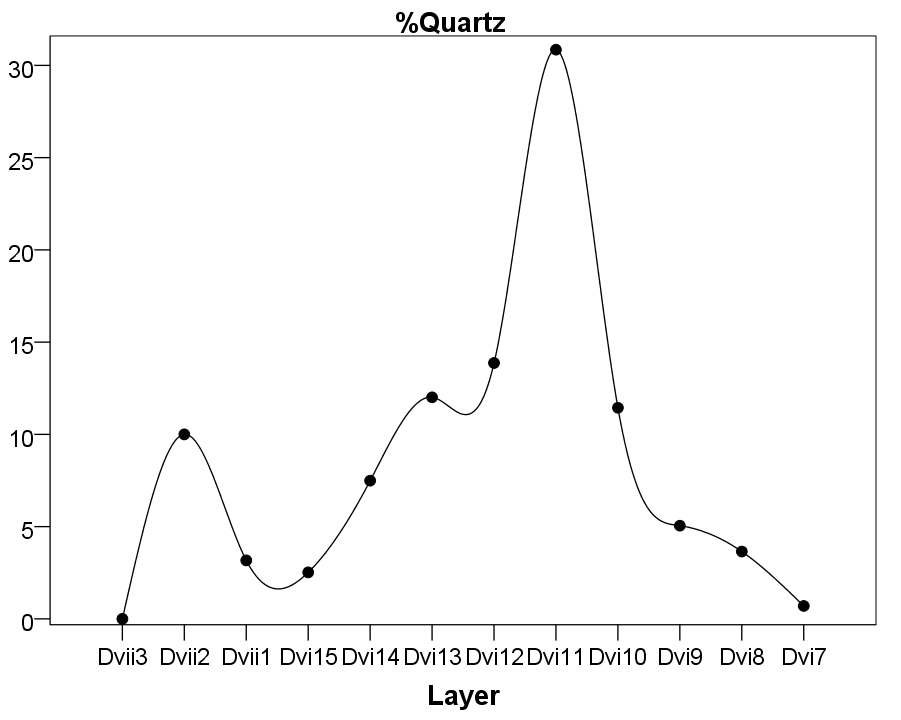


**Layer and %Backed pieces**

- Shows a general increase through time (but not significant in the non-parametric study!)

| **Model Summary and Parameter Estimates** | | | | | | | | | |
| --- | --- | --- | --- | --- | --- | --- | --- | --- | --- |
| Dependent Variable: BackedPiece | | | | | | | | | |
| Equation | Model Summary | | | | | Parameter Estimates | | | |
|  | R Square | F | df1 | df2 | Sig. | Constant | b1 | b2 | b3 |
| Linear | .386 | 6.280 | 1 | 10 | .031 | 65.830 | 4.643 |  |  |
| Cubic | .494 | 2.603 | 3 | 8 | .124 | 48.137 | -8.719 | -1.917 | -.069 |
| The independent variable is Layer. | | | | | | | | | |


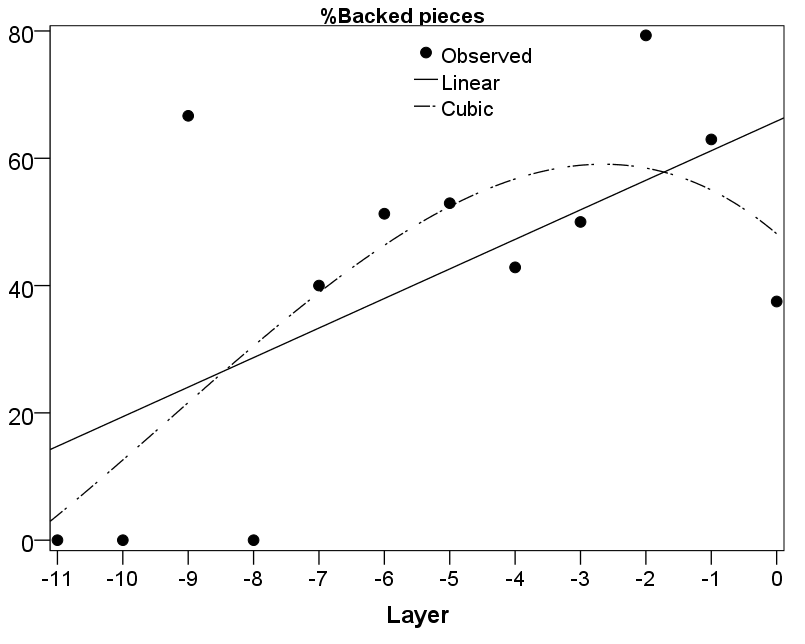


**Layer and %Notched Piece**

- Shows a curving (multimodal) pattern through time, no consistent increase or decrease


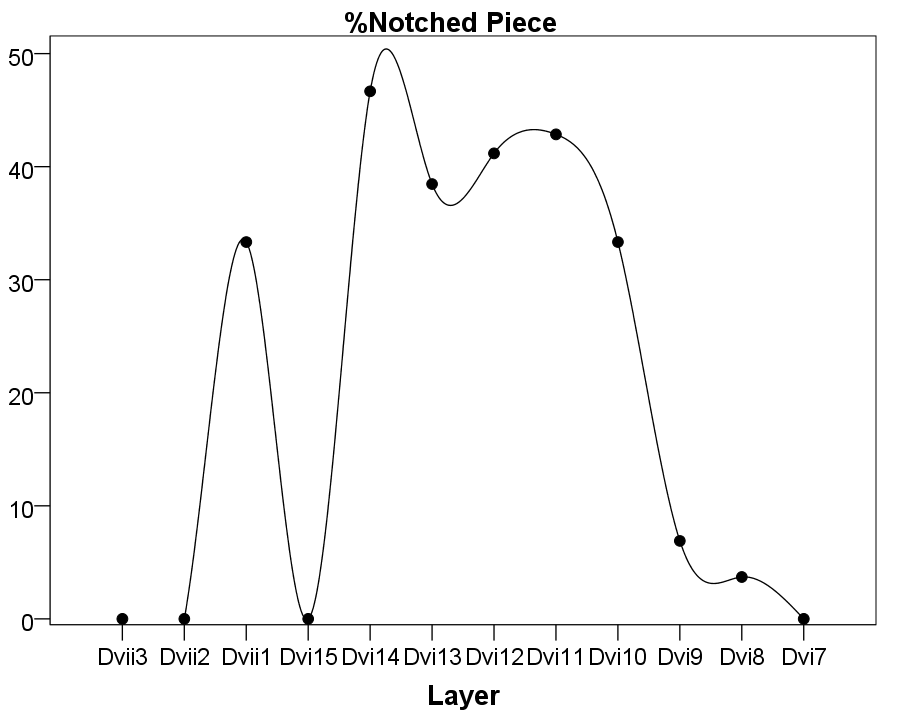


**Layer and %Elongation >2 (matches blades&bladelets)**

- Shows a curving (cubic) pattern through time, with a slight tendency to decrease (but not significant)

| **Model Summary and Parameter Estimates** | | | | | | | | | |
| --- | --- | --- | --- | --- | --- | --- | --- | --- | --- |
| Dependent Variable: Elongate2 | | | | | | | | | |
| Equation | Model Summary | | | | | Parameter Estimates | | | |
|  | R Square | F | df1 | df2 | Sig. | Constant | b1 | b2 | b3 |
| Linear | .157 | 1.868 | 1 | 10 | .202 | 16.188 | -.740 |  |  |
| Cubic | .607 | 4.114 | 3 | 8 | .049 | 14.232 | -6.631 | -1.742 | -.119 |
| The independent variable is Layer. | | | | | | | | | |


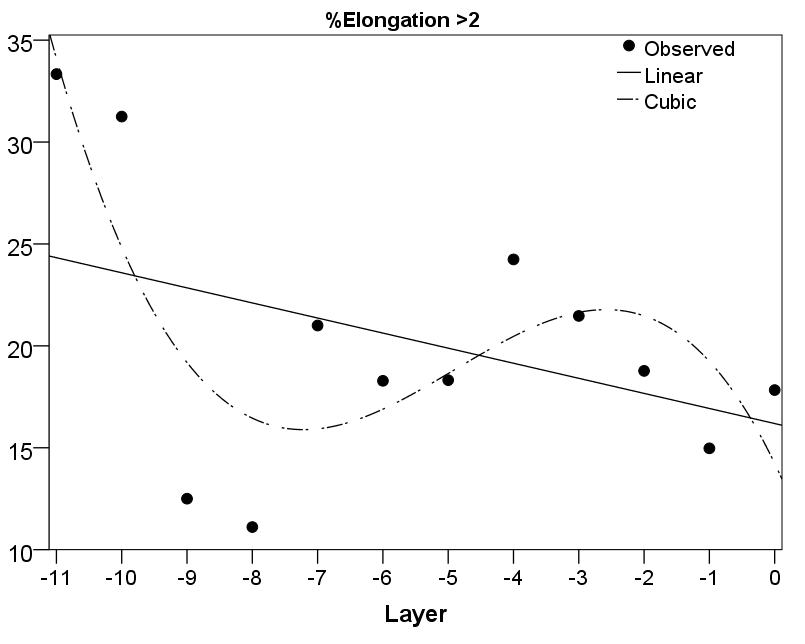


**S4 Text.** Summary of statistical analyses for the HP sequence at Klasies River. Raw data on lithic attributes mostly from Singer & Wymer (1982), with additional data from Wurz (2000) noted when used.

**a) Kendall’s tau correlations summary (individual SPSS output below)**

- Unidirectional trends are found **only for quartz and quartzite**!
  - INCREASE in quartz throughout HP sequence (significant, but not monotonic)
  - DECREASE in quartzite throughout HP sequence (significant, but not monotonic)
- No unidirectional trends are found for: silcrete, nonquartzite, silcreteblades, silcretesegments, backed pieces, notched piece, blades, bladestools, meanlengthbackedpieces, segmentshape, trapezeshape
  - Characteristic is a **curving pattern** for %silcrete and %notches with a peak in the middle of the sequence and a **double-curve** for %backed pieces and %blades with 2 peaks
- Also: No significant correlation between %silcrete and %backedpiece (t=0000; p=1.00) and between %backedpiece and %blade (t=0.031; p=0.890)

**Nonparametric Correlations (Layer and %silcrete)**

|  | | | Layer | Silcrete |
| --- | --- | --- | --- | --- |
| Kendall's tau_b | Layer | Correlation Coefficient | 1.000 | .030 |
|  |  | Sig. (2-tailed) | . | .891 |
|  |  | N | 12 | 12 |
|  | Silcrete | Correlation Coefficient | .030 | 1.000 |
|  |  | Sig. (2-tailed) | .891 | . |
|  |  | N | 12 | 12 |

**Nonparametric Correlations (Layer and %non-quartzite); data from Wurz (2000: Table 16)**

|  | | | | |
| --- | --- | --- | --- | --- |
|  | | | Layer | NonQuartzite |
| Kendall's tau_b | Layer | Correlation Coefficient | 1.000 | -.400 |
|  |  | Sig. (2-tailed) | . | .327 |
|  |  | N | 5 | 5 |
|  | NonQuartzite | Correlation Coefficient | -.400 | 1.000 |
|  |  | Sig. (2-tailed) | .327 | . |
|  |  | N | 5 | 5 |

**Nonparametric Correlations (Layer and %quartz)**

|  | | | Layer | Quartz |
| --- | --- | --- | --- | --- |
| Kendall's tau_b | Layer | Correlation Coefficient | 1.000 | **.595^**^** |
|  |  | Sig. (2-tailed) | . | .007 |
|  |  | N | 12 | 12 |
|  | Quartz | Correlation Coefficient | .595^**^ | 1.000 |
|  |  | Sig. (2-tailed) | .007 | . |
|  |  | N | 12 | 12 |
| **. Correlation is significant at the 0.01 level (2-tailed). | | | | |

**Nonparametric Correlations (Layer and %quartzite)**

|  | | | Layer | Quartzite |
| --- | --- | --- | --- | --- |
| Kendall's tau_b | Layer | Correlation Coefficient | 1.000 | **-.515^*^** |
|  |  | Sig. (2-tailed) | . | .020 |
|  |  | N | 12 | 12 |
|  | Quartzite | Correlation Coefficient | -.515^*^ | 1.000 |
|  |  | Sig. (2-tailed) | .020 | . |
|  |  | N | 12 | 12 |
| *. Correlation is significant at the 0.05 level (2-tailed). | | | | |

**Nonparametric Correlations (Layer and %silcrete all segments)**

|  | | | | |
| --- | --- | --- | --- | --- |
|  | | | Layer | SegmentSilcrete |
| Kendall's tau_b | Layer | Correlation Coefficient | 1.000 | -.273 |
|  |  | Sig. (2-tailed) | . | .217 |
|  |  | N | 12 | 12 |
|  | SegmentSilcrete | Correlation Coefficient | -.273 | 1.000 |
|  |  | Sig. (2-tailed) | .217 | . |
|  |  | N | 12 | 12 |

**Nonparametric Correlations (Layer and %silcrete all blades)**

|  | | | | |
| --- | --- | --- | --- | --- |
|  | | | Layer | BladeSilcrete |
| Kendall's tau_b | Layer | Correlation Coefficient | 1.000 | .000 |
|  |  | Sig. (2-tailed) | . | 1.000 |
|  |  | N | 12 | 12 |
|  | BladeSilcrete | Correlation Coefficient | .000 | 1.000 |
|  |  | Sig. (2-tailed) | 1.000 | . |
|  |  | N | 12 | 12 |

**Nonparametric Correlations (Layer and %backed piece)**

|  | | | | |
| --- | --- | --- | --- | --- |
|  | | | Layer | BackedPiece |
| Kendall's tau_b | Layer | Correlation Coefficient | 1.000 | .061 |
|  |  | Sig. (2-tailed) | . | .784 |
|  |  | N | 12 | 12 |
|  | BackedPiece | Correlation Coefficient | .061 | 1.000 |
|  |  | Sig. (2-tailed) | .784 | . |
|  |  | N | 12 | 12 |

**Nonparametric Correlations (Layer and %notchedpiece)**

|  | | | | |
| --- | --- | --- | --- | --- |
|  | | | Layer | Notching |
| Kendall's tau_b | Layer | Correlation Coefficient | 1.000 | -.154 |
|  |  | Sig. (2-tailed) | . | .491 |
|  |  | N | 12 | 12 |
|  | Notching | Correlation Coefficient | -.154 | 1.000 |
|  |  | Sig. (2-tailed) | .491 | . |
|  |  | N | 12 | 12 |

**Nonparametric Correlations (Layer and %blade)**

|  | | | | |
| --- | --- | --- | --- | --- |
|  | | | Layer | BladeSilcrete |
| Kendall's tau_b | Layer | Correlation Coefficient | 1.000 | .000 |
|  |  | Sig. (2-tailed) | . | 1.000 |
|  |  | N | 12 | 12 |
|  | BladeSilcrete | Correlation Coefficient | .000 | 1.000 |
|  |  | Sig. (2-tailed) | 1.000 | . |
|  |  | N | 12 | 12 |

**Nonparametric Correlations (Layer and %blade including segments)**

|  | | | | |
| --- | --- | --- | --- | --- |
|  | | | Layer | BladesWtools |
| Kendall's tau_b | Layer | Correlation Coefficient | 1.000 | -.061 |
|  |  | Sig. (2-tailed) | . | .784 |
|  |  | N | 12 | 12 |
|  | BladesWtools | Correlation Coefficient | -.061 | 1.000 |
|  |  | Sig. (2-tailed) | .784 | . |
|  |  | N | 12 | 12 |

**Nonparametric Correlations (Layer and mean length of backed pieces), data from Wurz (2000: Table 84)**

|  | | | | |
| --- | --- | --- | --- | --- |
|  | | | Layer | BackedPieceLength |
| Kendall's tau_b | Layer | Correlation Coefficient | 1.000 | -.412 |
|  |  | Sig. (2-tailed) | . | .063 |
|  |  | N | 12 | 12 |
|  | BackedPieceLength | Correlation Coefficient | -.412 | 1.000 |
|  |  | Sig. (2-tailed) | .063 | . |
|  |  | N | 12 | 12 |

**Nonparametric Correlations (Layer and CV mean length backed pieces), data from Wurz (2000: Table 94)**

|  | | | | |
| --- | --- | --- | --- | --- |
|  | | | Layer | CVBackedPieceLength |
| Kendall's tau_b | Layer | Correlation Coefficient | 1.000 | .109 |
|  |  | Sig. (2-tailed) | . | .628 |
|  |  | N | 12 | 12 |
|  | CVBackedPieceLength | Correlation Coefficient | .109 | 1.000 |
|  |  | Sig. (2-tailed) | .628 | . |
|  |  | N | 12 | 12 |

**Nonparametric Correlations (Layer and %segmentshape), data from Wurz (2000: Table 97)**

|  | | | | |
| --- | --- | --- | --- | --- |
|  | | | Layer | PercentSegment |
| Kendall's tau_b | Layer | Correlation Coefficient | 1.000 | .260 |
|  |  | Sig. (2-tailed) | . | .243 |
|  |  | N | 12 | 12 |
|  | PercentSegment | Correlation Coefficient | .260 | 1.000 |
|  |  | Sig. (2-tailed) | .243 | . |
|  |  | N | 12 | 12 |

**Nonparametric Correlations (Layer and %trapezeshape), data from Wurz (2000: Table 97)**

|  | | | | |
| --- | --- | --- | --- | --- |
|  | | | Layer | PercentTrapeze |
| Kendall's tau_b | Layer | Correlation Coefficient | 1.000 | -.137 |
|  |  | Sig. (2-tailed) | . | .536 |
|  |  | N | 12 | 12 |
|  | PercentTrapeze | Correlation Coefficient | -.137 | 1.000 |
|  |  | Sig. (2-tailed) | .536 | . |
|  |  | N | 12 | 12 |

**b) Scatter plots:**

**Layer and %Silcrete, %SegmentSilcrete and %BladeSilcrete**

- Shows a non-directional pattern through time with 1 peak in the middle of the sequence


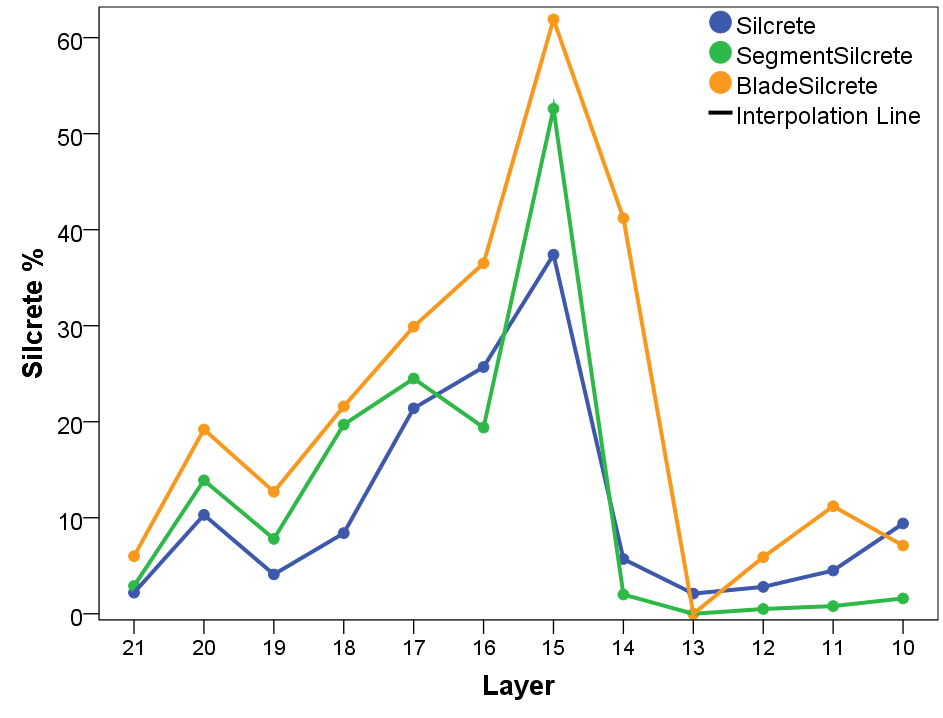


**Layer and %Non-Quartzite (data from Wurz 2000: Table 16)**

- Shows a non-directional pattern through time with 1 peak in the middle of the sequence


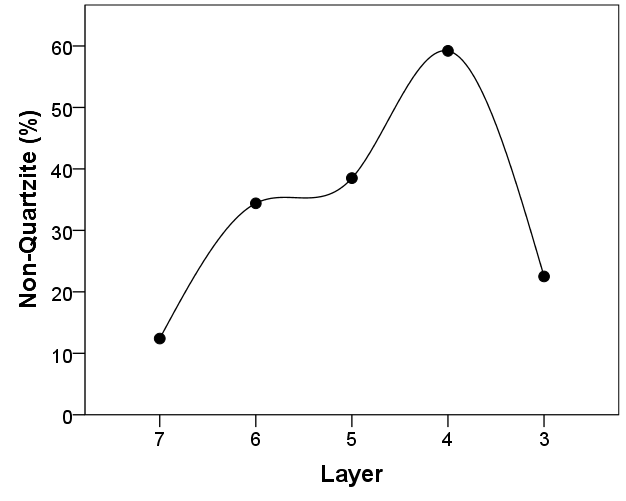


**Layer and %Quartz / %Quartzite**

- Both show curving patterns through time, but with a general increase in quartz and decrease in quartzite


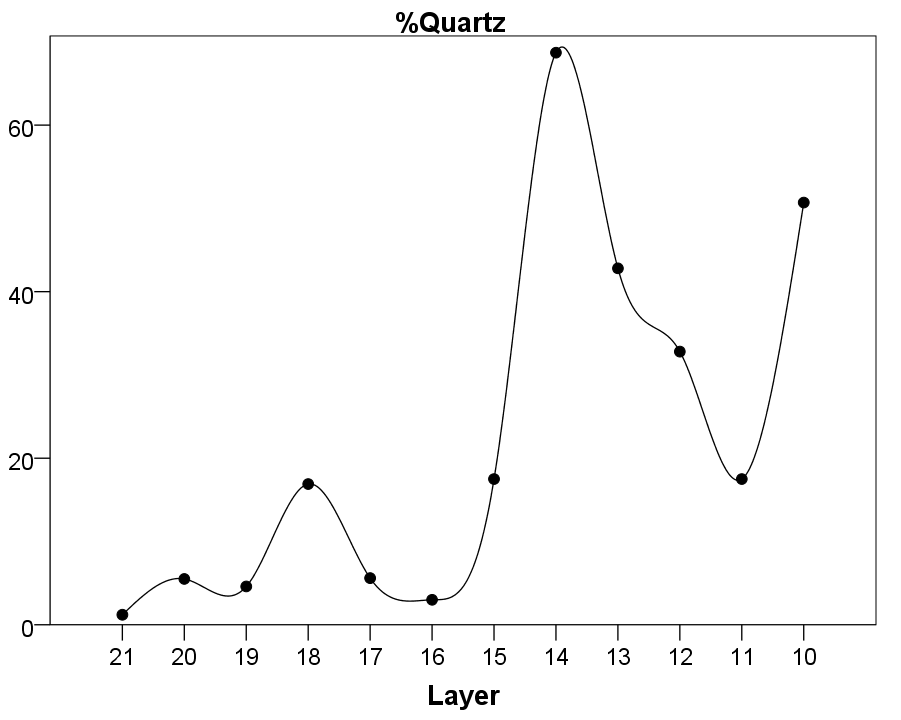


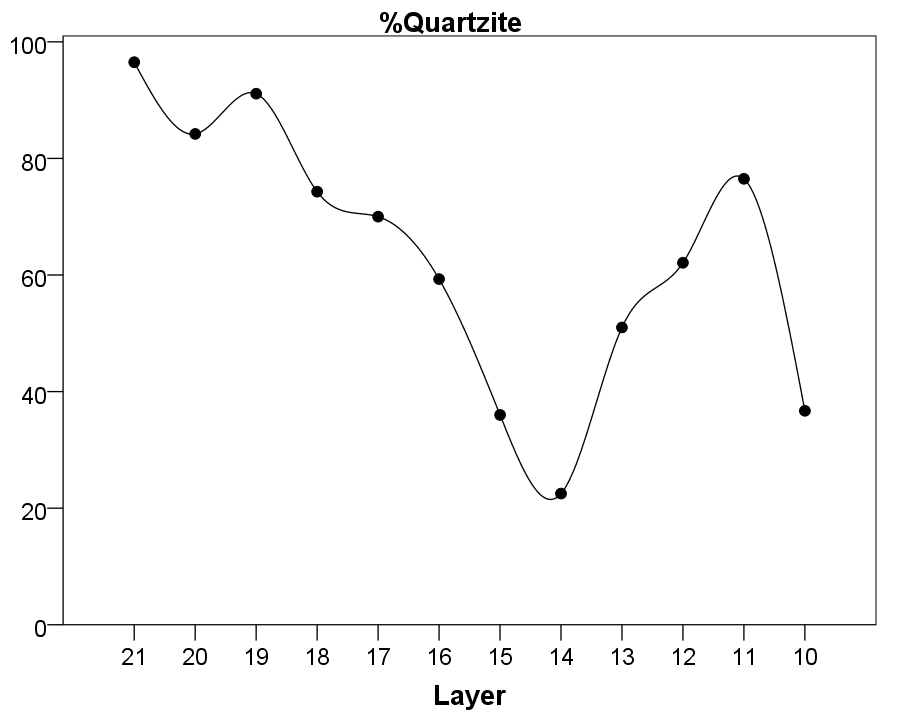


**Layer and %Backed pieces / %notched pieces**

- Both show curving pattern through time with 2 peaks (backed pieces) and 1 peak (notches)


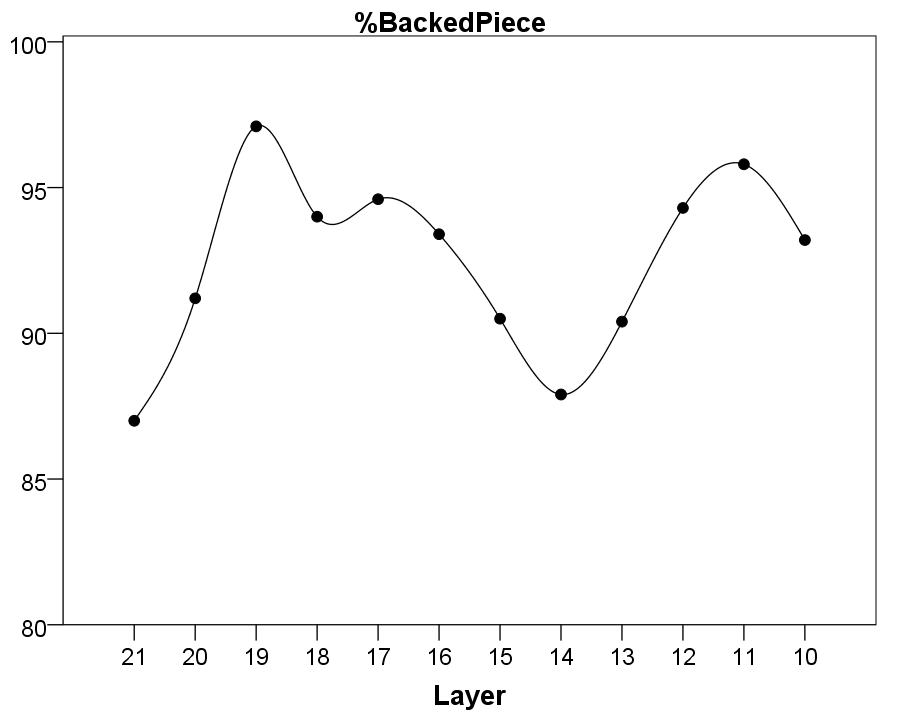


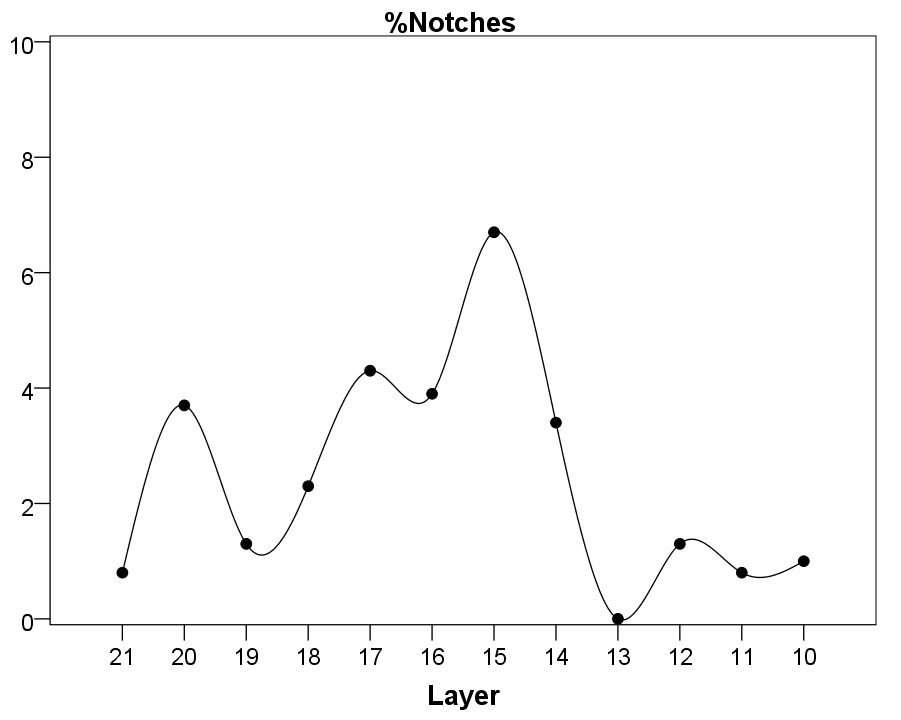


**Layer and %blade**

- Non-directional but curving pattern through time with 2 peaks in the middle and upper part


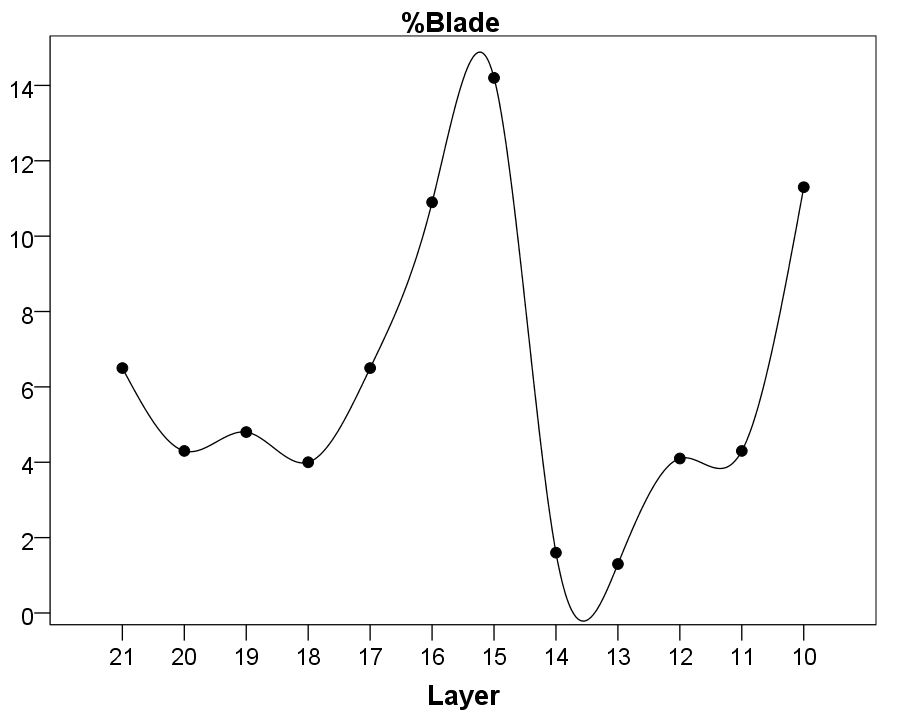


**Layer and mean length of backed pieces (data from Wurz 2000: Table 94)**

- Non-directional but curving pattern through time with 1 peaks in the early part of the sequence


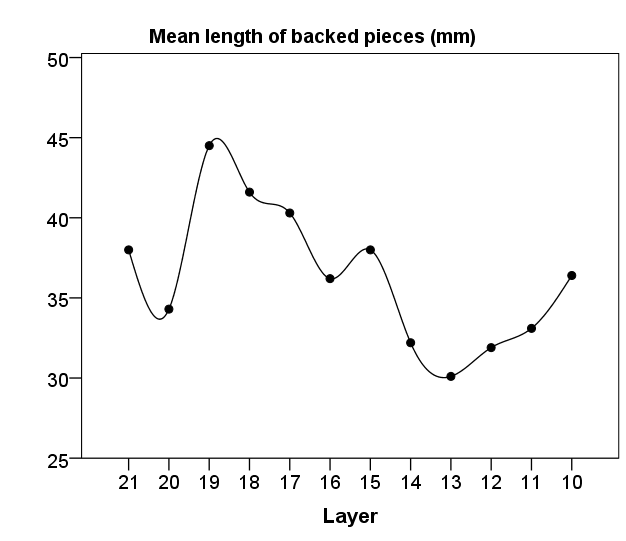


**Layer and mean length of backed pieces (data from Wurz 2000: Table 94)**

- Non-directional but curving pattern through time with 1 peaks in the early part of the sequence


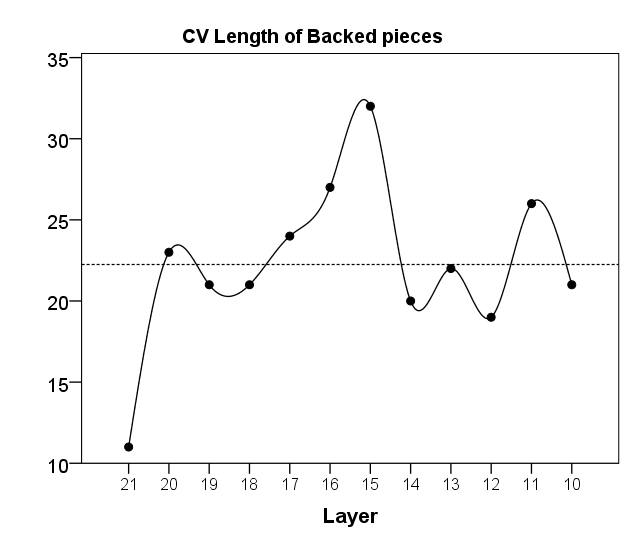


**Layer and shape of backed pieces (data from Wurz 2000: Table 97)**

- Non-directional but curving pattern through time with 2 peak (segments) and 1 peak (trapeze) in the early part of the sequence


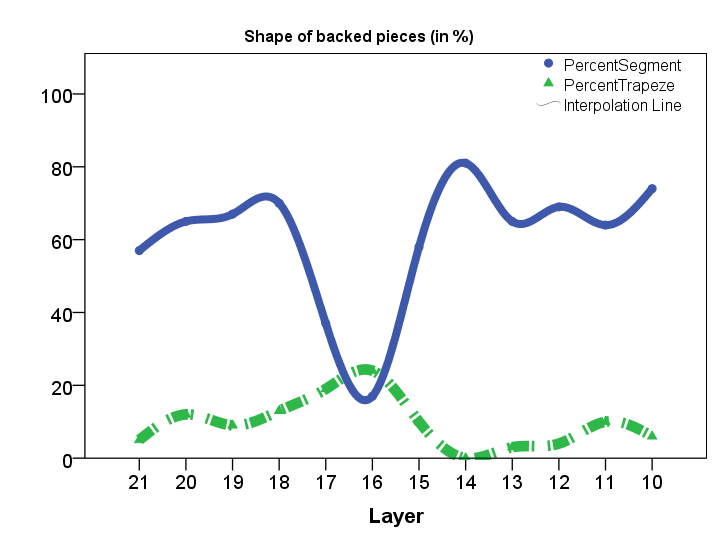


**S5 Text.** Summary of statistical analyses for the HP sequence at Klipdrift Rockshelter. Raw data on lithic attributes from Mackay (2010, 2011) and Mackay (own data, personal communication).

**a) Kendall’s tau correlations summary (individual SPSS output below)**

- Significant unidirectional trends are found **only for blade thickness**!
  - INCREASE in blade thickness throughout HP sequence (significant)
- No unidirectional trends are found for: silcrete, quartzite, quartz, backed pieces, blades, notched pieces, blade length, blade width
  - Characteristic is a **curving pattern** for silcrete, quartzite, quartz, backed pieces,
- Also: No significant correlation between %silcrete and %backedpiece (t=-0.524; p=0.099) and between %backedpiece and %blade (t=-0.048; p=0.881)
- No correlation between measures of site use intensity and backed pieces: Shell density (t=0.333; p=0.293), Macroauna density (t=0.333; p=0.293).
- But: Significant correlation between %quartz and %backedpiece (t=-0.810; p=0.011)

**Nonparametric Correlations (Layer and %silcrete)**

|  | | | | |
| --- | --- | --- | --- | --- |
|  | | | Layer | Silcrete |
| Kendall's tau_b | Layer | Correlation Coefficient | 1.000 | -.238 |
|  |  | Sig. (2-tailed) | . | .453 |
|  |  | N | 7 | 7 |
|  | Silcrete | Correlation Coefficient | -.238 | 1.000 |
|  |  | Sig. (2-tailed) | .453 | . |
|  |  | N | 7 | 7 |

**Nonparametric Correlations (Layer and %quartz)**

|  | | | | |
| --- | --- | --- | --- | --- |
|  | | | Layer | Quartz |
| Kendall's tau_b | Layer | Correlation Coefficient | 1.000 | .143 |
|  |  | Sig. (2-tailed) | . | .652 |
|  |  | N | 7 | 7 |
|  | Quartz | Correlation Coefficient | .143 | 1.000 |
|  |  | Sig. (2-tailed) | .652 | . |
|  |  | N | 7 | 7 |

**Nonparametric Correlations (Layer and %quartzite)**

|  | | | | |
| --- | --- | --- | --- | --- |
|  | | | Layer | Quartzite |
| Kendall's tau_b | Layer | Correlation Coefficient | 1.000 | .143 |
|  |  | Sig. (2-tailed) | . | .652 |
|  |  | N | 7 | 7 |
|  | Quartzite | Correlation Coefficient | .143 | 1.000 |
|  |  | Sig. (2-tailed) | .652 | . |
|  |  | N | 7 | 7 |

**Nonparametric Correlations (Layer and %backed piece)**

|  | | | | |
| --- | --- | --- | --- | --- |
|  | | | Layer | BackedPiece |
| Kendall's tau_b | Layer | Correlation Coefficient | 1.000 | .143 |
|  |  | Sig. (2-tailed) | . | .652 |
|  |  | N | 7 | 7 |
|  | BackedPiece | Correlation Coefficient | .143 | 1.000 |
|  |  | Sig. (2-tailed) | .652 | . |
|  |  | N | 7 | 7 |

**Nonparametric Correlations (Layer and %notchedpiece)**

|  | | | | |
| --- | --- | --- | --- | --- |
|  | | | Layer | Notching |
| Kendall's tau_b | Layer | Correlation Coefficient | 1.000 | -.333 |
|  |  | Sig. (2-tailed) | . | .293 |
|  |  | N | 7 | 7 |
|  | Notching | Correlation Coefficient | -.333 | 1.000 |
|  |  | Sig. (2-tailed) | .293 | . |
|  |  | N | 7 | 7 |

**Nonparametric Correlations (Layer and %blade)**

|  | | | | |
| --- | --- | --- | --- | --- |
|  | | | Layer | Blades |
| Kendall's tau_b | Layer | Correlation Coefficient | 1.000 | -.429 |
|  |  | Sig. (2-tailed) | . | .176 |
|  |  | N | 7 | 7 |
|  | Blades | Correlation Coefficient | -.429 | 1.000 |
|  |  | Sig. (2-tailed) | .176 | . |
|  |  | N | 7 | 7 |

**Nonparametric Correlations (Layer and MeanBladeLength)**

|  | | | | |
| --- | --- | --- | --- | --- |
|  | | | Layer | BladeLength |
| Kendall's tau_b | Layer | Correlation Coefficient | 1.000 | .143 |
|  |  | Sig. (2-tailed) | . | .652 |
|  |  | N | 7 | 7 |
|  | BladeLength | Correlation Coefficient | .143 | 1.000 |
|  |  | Sig. (2-tailed) | .652 | . |
|  |  | N | 7 | 7 |

**Nonparametric Correlations (Layer and MeanBladeWidth)**

|  | | | | |
| --- | --- | --- | --- | --- |
|  | | | Layer | Bladewidth |
| Kendall's tau_b | Layer | Correlation Coefficient | 1.000 | .524 |
|  |  | Sig. (2-tailed) | . | .099 |
|  |  | N | 7 | 7 |
|  | Bladewidth | Correlation Coefficient | .524 | 1.000 |
|  |  | Sig. (2-tailed) | .099 | . |
|  |  | N | 7 | 7 |

**Nonparametric Correlations (Layer and MeanBladeThickness)**

|  | | | | |
| --- | --- | --- | --- | --- |
|  | | | Layer | BladeThick |
| Kendall's tau_b | Layer | Correlation Coefficient | 1.000 | **.714^*^** |
|  |  | Sig. (2-tailed) | . | .024 |
|  |  | N | 7 | 7 |
|  | BladeThick | Correlation Coefficient | .714^*^ | 1.000 |
|  |  | Sig. (2-tailed) | .024 | . |
|  |  | N | 7 | 7 |
| *. Correlation is significant at the 0.05 level (2-tailed). | | | | |

**Nonparametric Correlations (Layer and %Cortex0)**

|  | | | | |
| --- | --- | --- | --- | --- |
|  | | | Layer | Cortex0 |
| Kendall's tau_b | Layer | Correlation Coefficient | 1.000 | -.333 |
|  |  | Sig. (2-tailed) | . | .293 |
|  |  | N | 7 | 7 |
|  | Cortex0 | Correlation Coefficient | -.333 | 1.000 |
|  |  | Sig. (2-tailed) | .293 | . |
|  |  | N | 7 | 7 |

**b) Scatter plots**

**Layer and %Silcrete**

- Shows a non-directional curving pattern through time with a minimum in the middle of the sequence


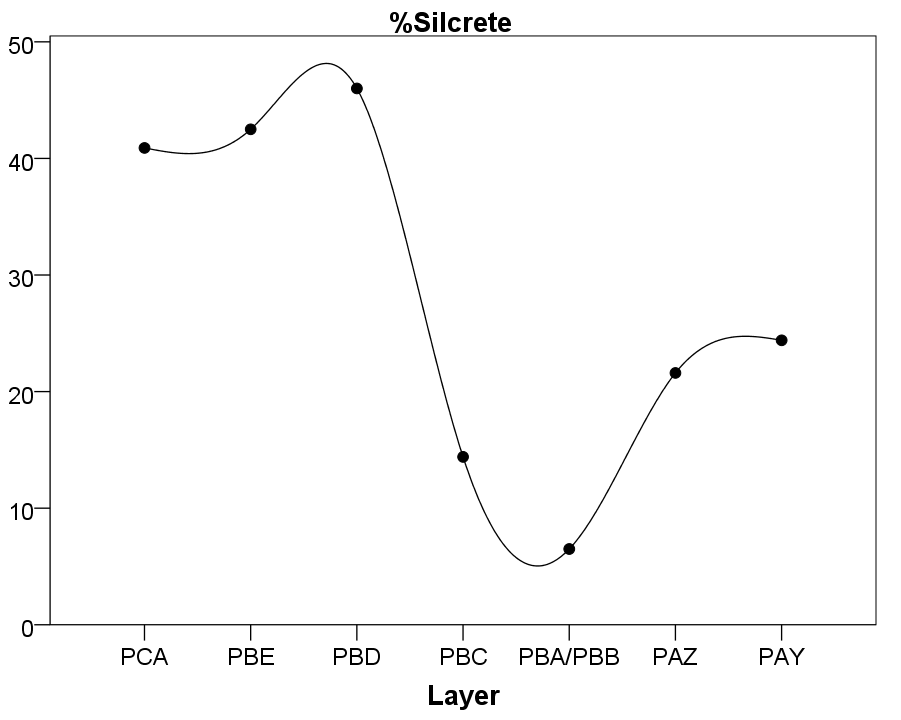


**Layer and %Quartz / %Quartzite**

- Both show non-directional curving pattern through time, with 2 peaks for quartzite


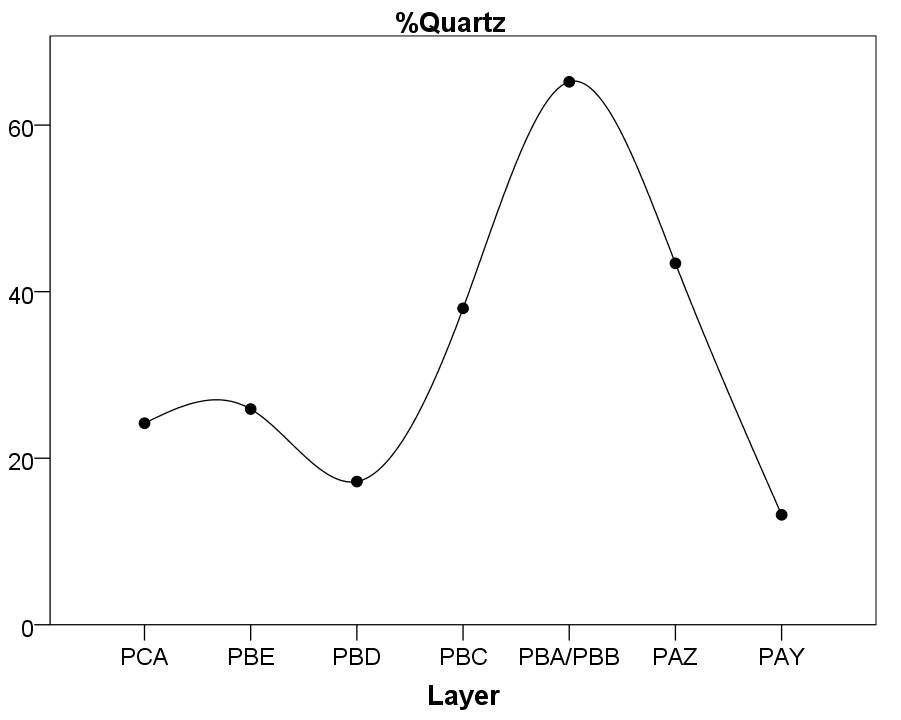


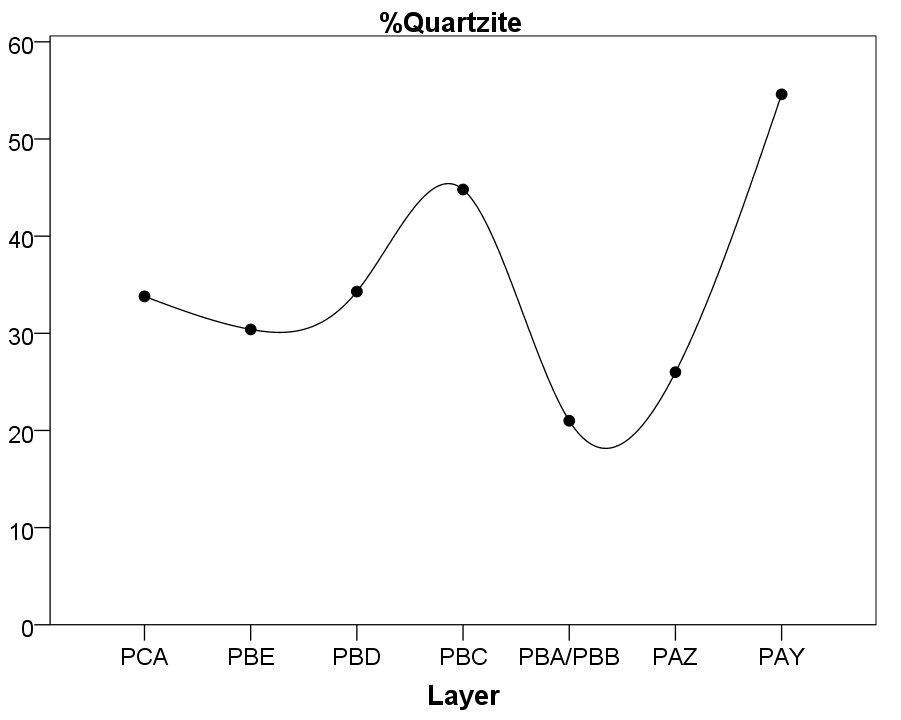


**Layer and %Backed pieces / %notched pieces**

- Backed piece: non-directional pattern with 1 peak in the middle
- Notch piece: general decrease through time but with a curving pattern


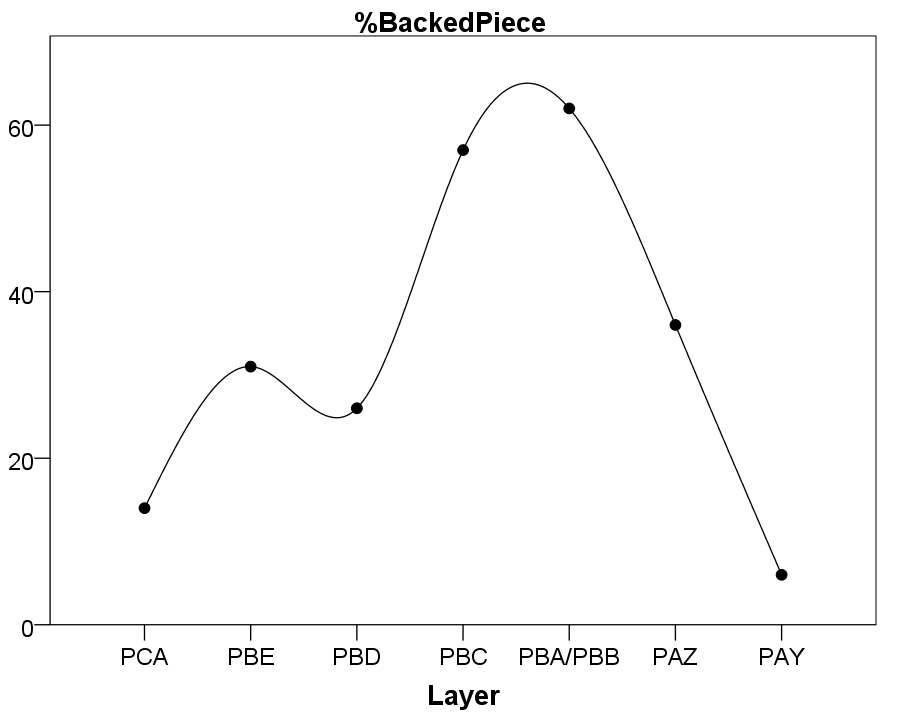


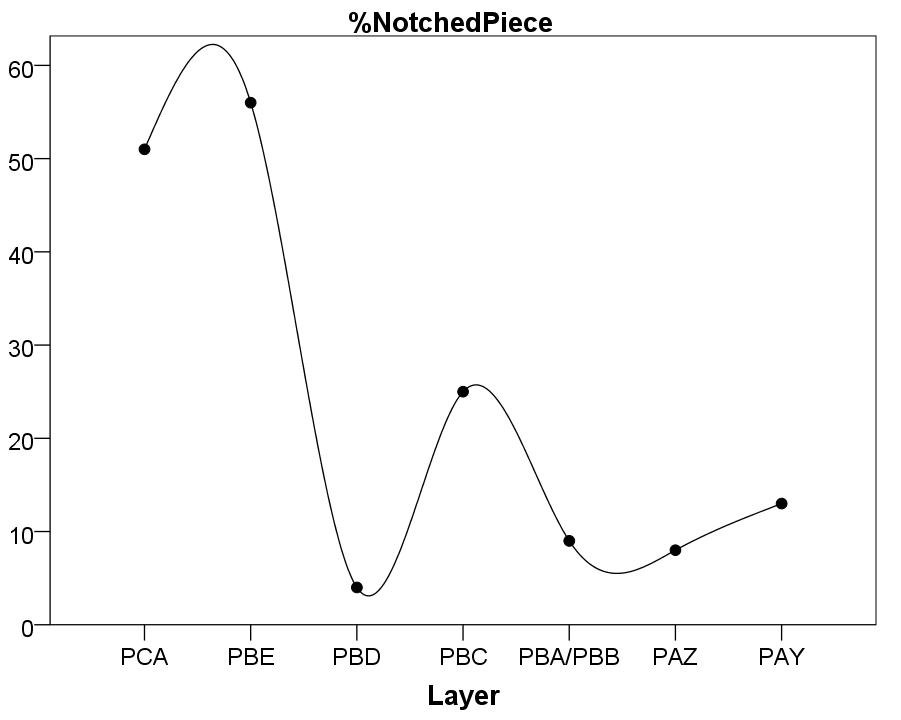


**Layer and %blade**

- Shows a non-directional pattern through time with one peak in early/middle part, though there is some decrease overall from top to bottom


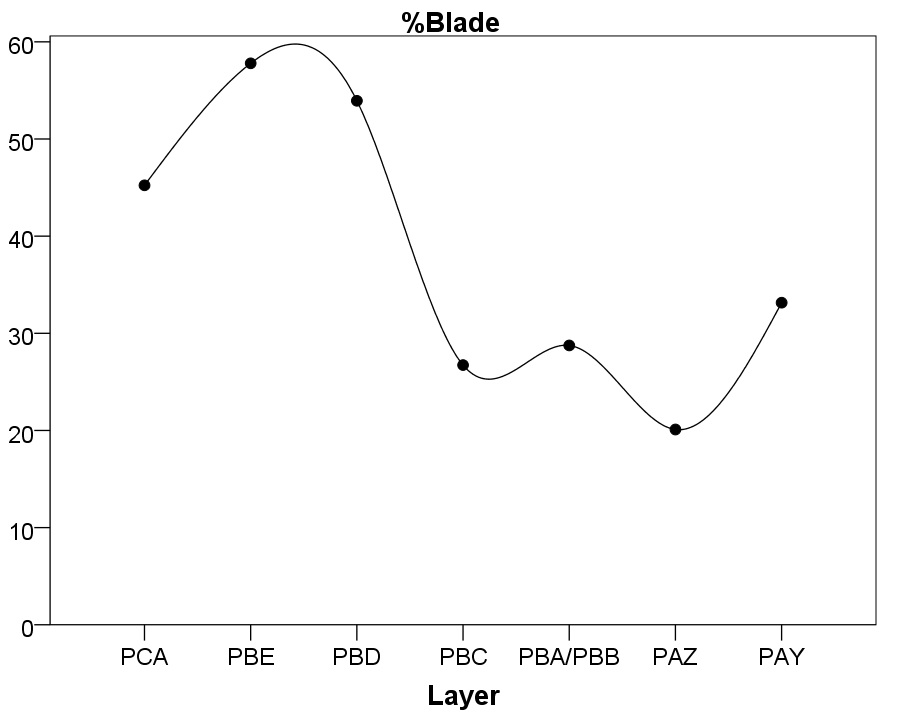


**Layer and Blade dimensions**

- Blade length (blue): Remains mostly constant
- Blade width (green): Remains mostly stable
- Blade thickness (beige): Unidirectional increase


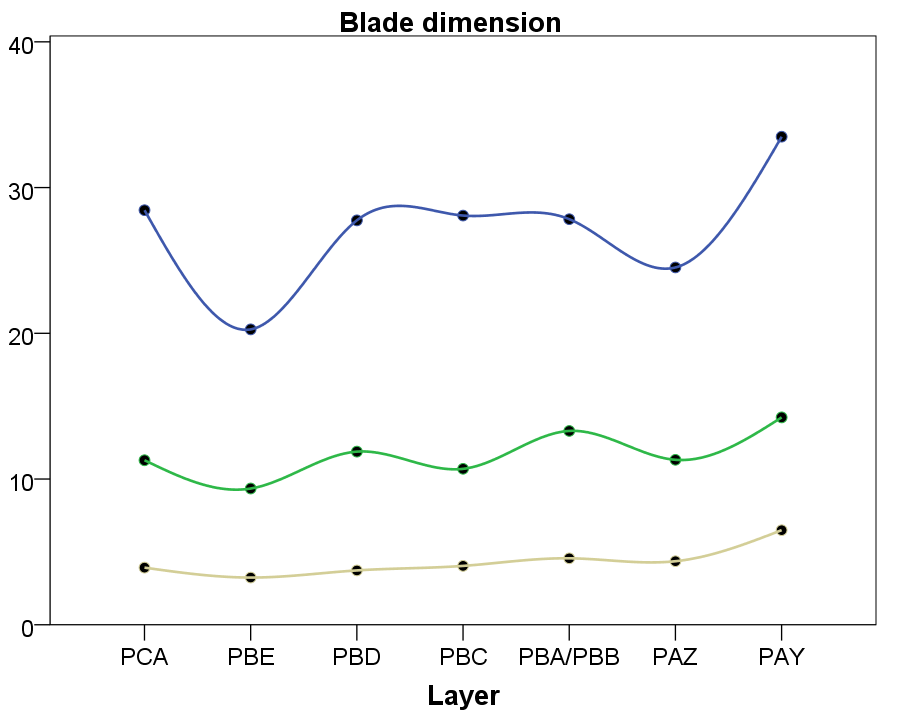


**S6 Text.** Summary of statistical analyses for the HP sequence at Pinnacle Point 5-6. Raw data on lithic attributes from Brown et al. (2012) and Wilkins et al. (2017).

**a) Kendall’s tau correlations summary (individual SPSS output below)**

- Significant unidirectional trends are found **only for segment width**!
  - INCREASE in segment width throughout HP sequence (significant); increase in backed pieces and notched pieces (but non-significant)
- No unidirectional trends are found for: silcrete, quartzite, backed pieces (total assemblage), blades, segment thickness, segment length, CV of segment length
  - Characteristic is a **curving pattern** for silcrete, blades, segment length, CV segment length (2 peaks), backed pieces and quartzite (1 peak)
- Also: No significant correlation between %silcrete and %backedpiece (t=-0.183; p=0.718)

**Nonparametric Correlations (Layer and %silcrete)**

|  | | | | |
| --- | --- | --- | --- | --- |
|  | | | Layer | Silcrete |
| Kendall's tau_b | Layer | Correlation Coefficient | 1.000 | .000 |
|  |  | Sig. (2-tailed) | . | 1.000 |
|  |  | N | 4 | 4 |
|  | Silcrete | Correlation Coefficient | .000 | 1.000 |
|  |  | Sig. (2-tailed) | 1.000 | . |
|  |  | N | 4 | 4 |

**Nonparametric Correlations (Layer and %quartzite)**

|  | | | | |
| --- | --- | --- | --- | --- |
|  | | | Layer | Quartzite |
| Kendall's tau_b | Layer | Correlation Coefficient | 1.000 | .000 |
|  |  | Sig. (2-tailed) | . | 1.000 |
|  |  | N | 4 | 4 |
|  | Quartzite | Correlation Coefficient | .000 | 1.000 |
|  |  | Sig. (2-tailed) | 1.000 | . |
|  |  | N | 4 | 4 |

**Nonparametric Correlations (Layer and %backed piece)**

|  | | | | |
| --- | --- | --- | --- | --- |
|  | | | Layer | BackedPiece |
| Kendall's tau_b | Layer | Correlation Coefficient | 1.000 | .548 |
|  |  | Sig. (2-tailed) | . | .279 |
|  |  | N | 4 | 4 |
|  | BackedPiece | Correlation Coefficient | .548 | 1.000 |
|  |  | Sig. (2-tailed) | .279 | . |
|  |  | N | 4 | 4 |

**Nonparametric Correlations (Layer and %notched piece)**

|  | | | Layer | NotchedPiece |
| --- | --- | --- | --- | --- |
| Kendall's tau_b | Layer | Correlation Coefficient | 1.000 | .913 |
|  |  | Sig. (2-tailed) | . | .071 |
|  |  | N | 4 | 4 |
|  | NotchedPiece | Correlation Coefficient | .913 | 1.000 |
|  |  | Sig. (2-tailed) | .071 | . |
|  |  | N | 4 | 4 |

**Nonparametric Correlations (Layer and %blade)**

|  | | | | |
| --- | --- | --- | --- | --- |
|  | | | Layer | Blades |
| Kendall's tau_b | Layer | Correlation Coefficient | 1.000 | -.200 |
|  |  | Sig. (2-tailed) | . | .624 |
|  |  | N | 5 | 5 |
|  | Blades | Correlation Coefficient | -.200 | 1.000 |
|  |  | Sig. (2-tailed) | .624 | . |
|  |  | N | 5 | 5 |

**Nonparametric Correlations (Layer and Segment metrics)**

|  | | | |
| --- | --- | --- | --- |
|  | | | Layer |
| Kendall's tau_b | Layer | Correlation Coefficient | 1.000 |
|  |  | Sig. (2-tailed) | . |
|  |  | N | 4 |
|  | SegmentLength | Correlation Coefficient | .333 |
|  |  | Sig. (2-tailed) | .602 |
|  |  | N | 3 |
|  | SegmentWidth | Correlation Coefficient | ****1.000** |
|  |  | Sig. (2-tailed) | . |
|  |  | N | 3 |
|  | SegmentThick | Correlation Coefficient | .333 |
|  |  | Sig. (2-tailed) | .602 |
|  |  | N | 3 |
|  | SegmentCVLength | Correlation Coefficient | .333 |
|  |  | Sig. (2-tailed) | .602 |
|  |  | N | 3 |

**b) Scatter plots**

**Layer and %Silcrete**

- Both silcrete and quartzite show a non-directional patterns through time (silcrete with 1 marked minimum, quartzite with 1 peak)


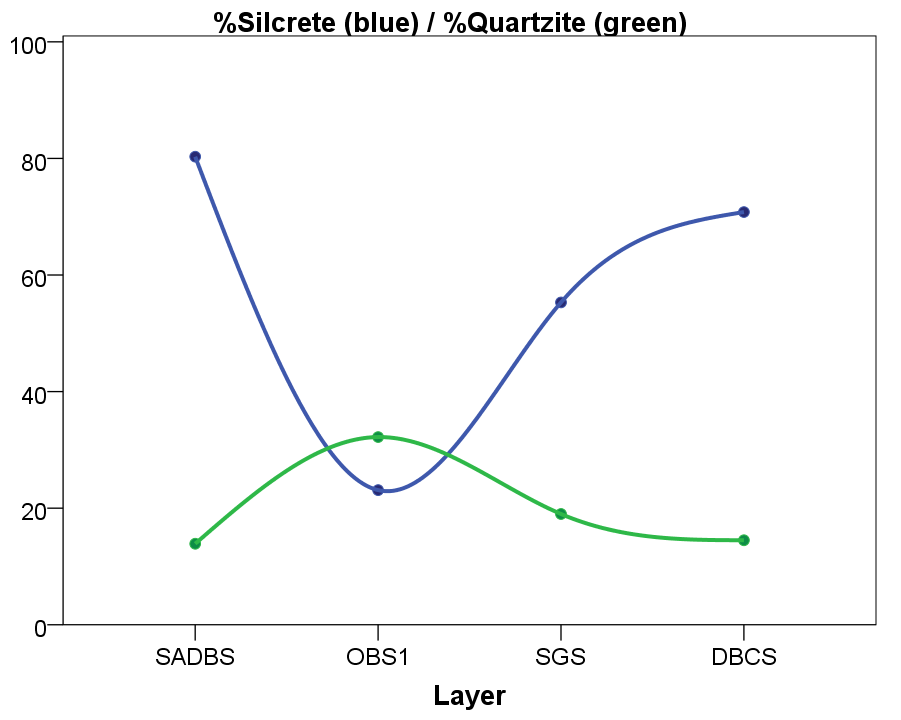


**Layer and %Backed pieces / %backedpieces TOTAL**

- Backed piece total assemblage: non-directional pattern through time (1 peak)
- Backed pieces by blanks+tools only: increase through time


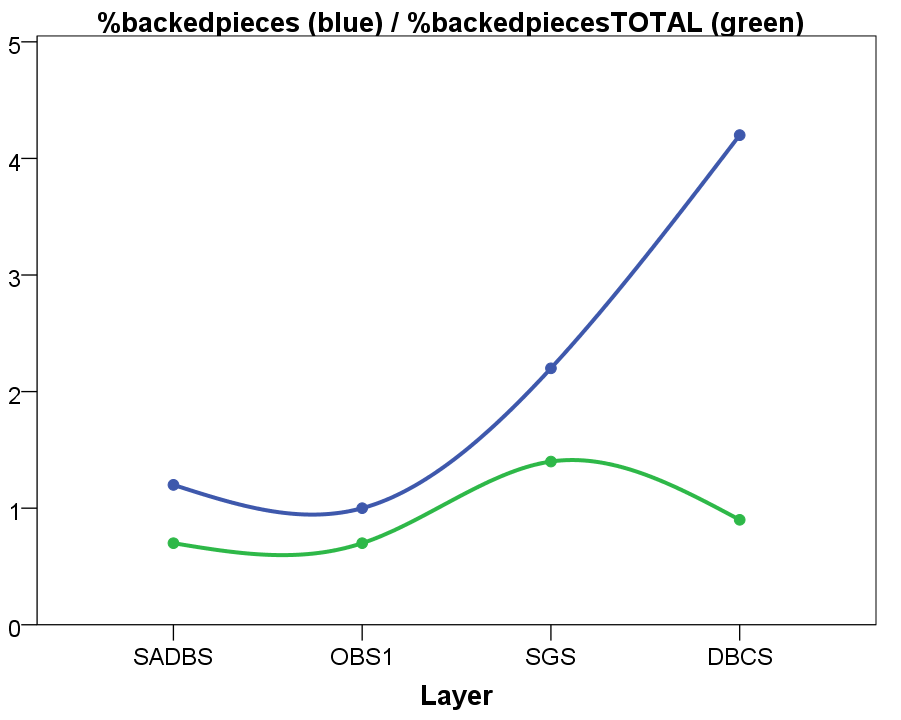


**Layer and %Notched Piece**

- Unidirectional increase through time


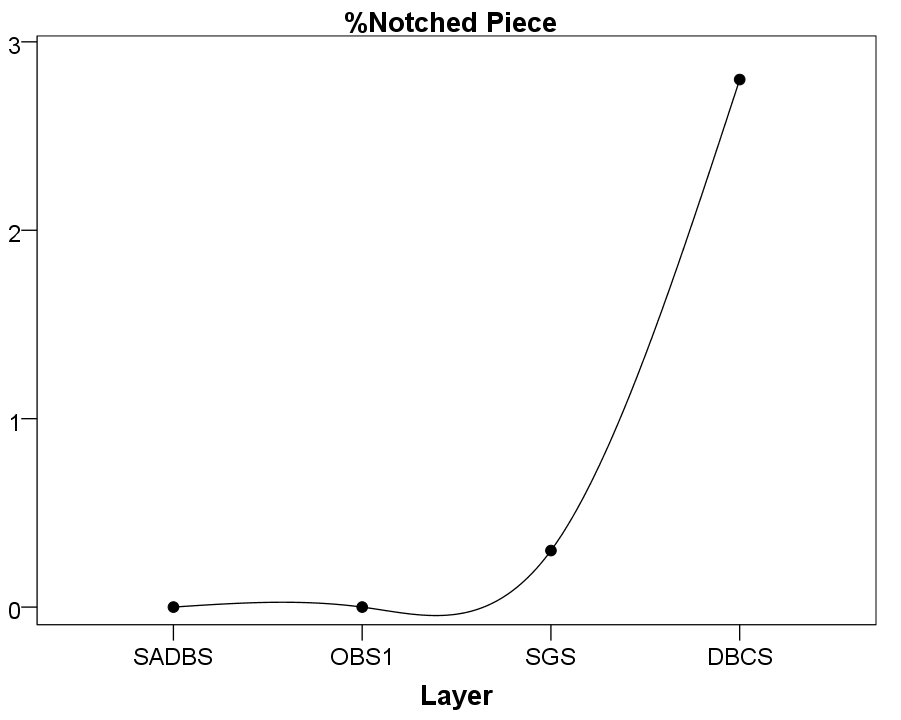


**Layer and %blade**

- Shows a non-directional curving pattern through time (with 3 peaks), with a slight increase again towards the end of the sequence


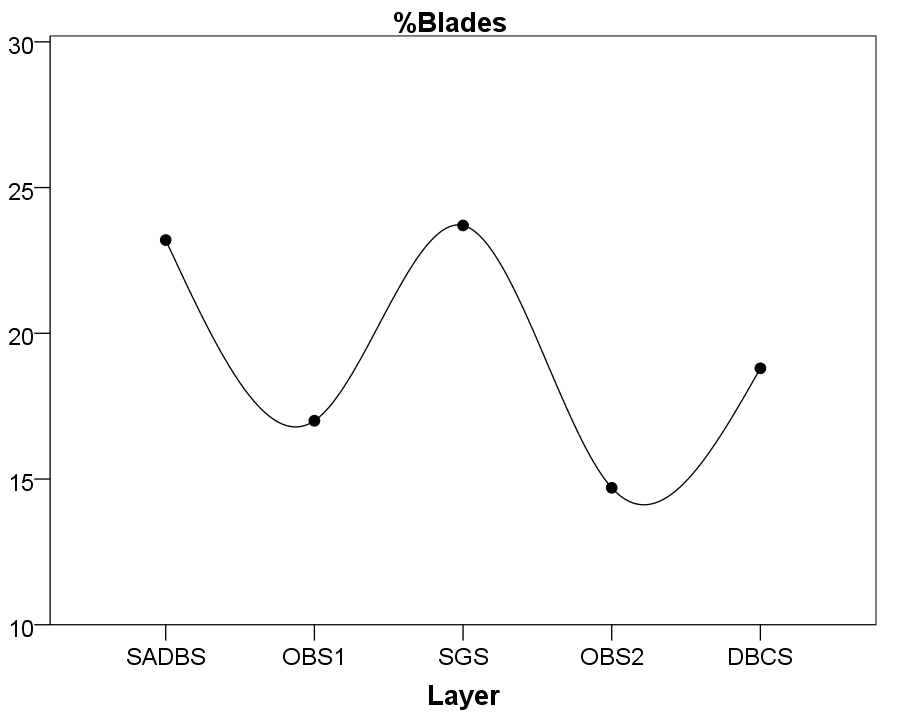


**Layer and Segment dimensions**

- Segment length (blue): Non-directional
- Segment width (green): Unidirectional increase
- Segmentthickness (beige): Non-directional, constant
- Segment CV length (purple): Non-directional


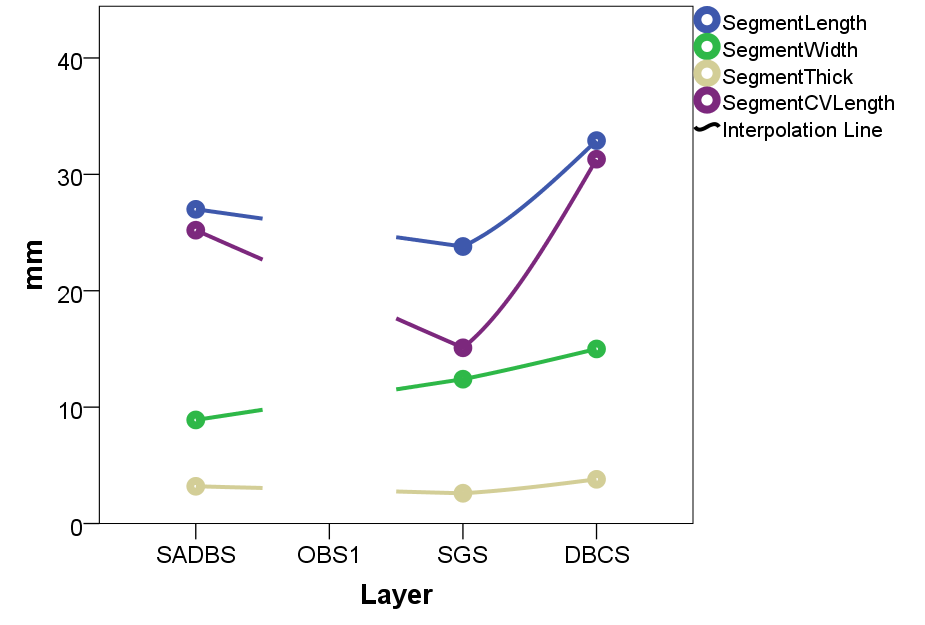


**S7 Text.** Summary of statistical analyses for the HP sequence at Rose Cottage Cave. Raw data on lithic attributes from Soriano et al. (2007).

**a) Kendall’s tau correlations summary (individual SPSS output below)**

- **Problem**: Only 4 (!) layers, which is very low even for Kendall-correlation. Thus an assessment of unidirectional trends also requires close inspection of the scatterplots.
- **Significant monotonous (!) unidirectional** trends are found **for %backedpieces, %blades, %abrasion and %impactpoint**
  - **Unidirectional trends** (though not significant and strictly monotonous) can be observed on the scatterplots **for %opaline, %tuff, %HPandBladeCores and %Platforms<2mm**,
    - INCREASE in %tuff, %impactpoint, %notches throughout HP sequence
    - DECREASE in %backedpieces, %blades, %HPandBladeCores, %opaline, %Platforms<2mm, %abrasion throughout HP sequence

**Nonparametric Correlations (Layer and %opaline)**

|  | | | | |
| --- | --- | --- | --- | --- |
|  | | | Layer | Tuff |
| Kendall's tau_b | Layer | Correlation Coefficient | 1.000 | .667 |
|  |  | Sig. (2-tailed) | . | .174 |
|  |  | N | 4 | 4 |
|  | Tuff | Correlation Coefficient | .667 | 1.000 |
|  |  | Sig. (2-tailed) | .174 | . |
|  |  | N | 4 | 4 |

**Nonparametric Correlations (Layer and %tuff)**

|  | | | | |
| --- | --- | --- | --- | --- |
|  | | | Layer | Tuff |
| Kendall's tau_b | Layer | Correlation Coefficient | 1.000 | .667 |
|  |  | Sig. (2-tailed) | . | .174 |
|  |  | N | 4 | 4 |
|  | Tuff | Correlation Coefficient | .667 | 1.000 |
|  |  | Sig. (2-tailed) | .174 | . |
|  |  | N | 4 | 4 |

**Nonparametric Correlations (Layer and %backed piece)**

|  | | | | |
| --- | --- | --- | --- | --- |
|  | | | Layer | BackedPiece |
| Kendall's tau_b | Layer | Correlation Coefficient | 1.000 | -1.000 |
|  |  | Sig. (2-tailed) | . | . |
|  |  | N | 4 | 3 |
|  | BackedPiece | Correlation Coefficient | **-1.000^**^** | 1.000 |
|  |  | Sig. (2-tailed) | . | . |
|  |  | N | 3 | 3 |
| **. Correlation is significant at the 0.01 level (2-tailed). | | | | |

**Nonparametric Correlations (Layer and %notched piece)**

|  | | | | |
| --- | --- | --- | --- | --- |
|  | | | NotchedPiece | Layer |
| Kendall's tau_b | NotchedPiece | Correlation Coefficient | 1.000 | .333 |
|  |  | Sig. (2-tailed) | . | .602 |
|  |  | N | 3 | 3 |
|  | Layer | Correlation Coefficient | .333 | 1.000 |
|  |  | Sig. (2-tailed) | .602 | . |
|  |  | N | 3 | 4 |

**Nonparametric Correlations (Layer and %HPandBladeCores)**

|  | | | | |
| --- | --- | --- | --- | --- |
|  | | | Layer | HPandBladeCores |
| Kendall's tau_b | Layer | Correlation Coefficient | 1.000 | -.333 |
|  |  | Sig. (2-tailed) | . | .497 |
|  |  | N | 4 | 4 |
|  | HPandBladeCores | Correlation Coefficient | -.333 | 1.000 |
|  |  | Sig. (2-tailed) | .497 | . |
|  |  | N | 4 | 4 |

**Nonparametric Correlations (Layer and %blade)**

|  | | | | |
| --- | --- | --- | --- | --- |
|  | | | Layer | BackedPiece |
| Kendall's tau_b | Layer | Correlation Coefficient | 1.000 | -1.000 |
|  |  | Sig. (2-tailed) | . | . |
|  |  | N | 4 | 3 |
|  | BackedPiece | Correlation Coefficient | **-1.000^**^** | 1.000 |
|  |  | Sig. (2-tailed) | . | . |
|  |  | N | 3 | 3 |
| **. Correlation is significant at the 0.01 level (2-tailed). | | | | |

**Nonparametric Correlations (Layer and various knapping traces)**

|  | | | | | | | | |
| --- | --- | --- | --- | --- | --- | --- | --- | --- |
|  | | | Layer | Abrasion | Platform2mm | ImpactPoint | Lip | ShatteredBulb |
| Kendall's tau_b | Layer | Correlation Coefficient | 1.000 | **-1.000^*^** | -.667 | **1.000^*^** | -.333 | .000 |
|  |  | Sig. (2-tailed) | . | . | .174 | . | .497 | 1.000 |
|  |  | N | 4 | 4 | 4 | 4 | 4 | 4 |
| *. Correlation is significant at the 0.05 level (2-tailed). | | | | | | | | |
| **. Correlation is significant at the 0.01 level (2-tailed). | | | | | | | | |

**b) Scatter plots**

**Layer and %Opaline / %Tuff**

- Both show directional trends, with opaline showing a relative consistent decrease and tuff an increase


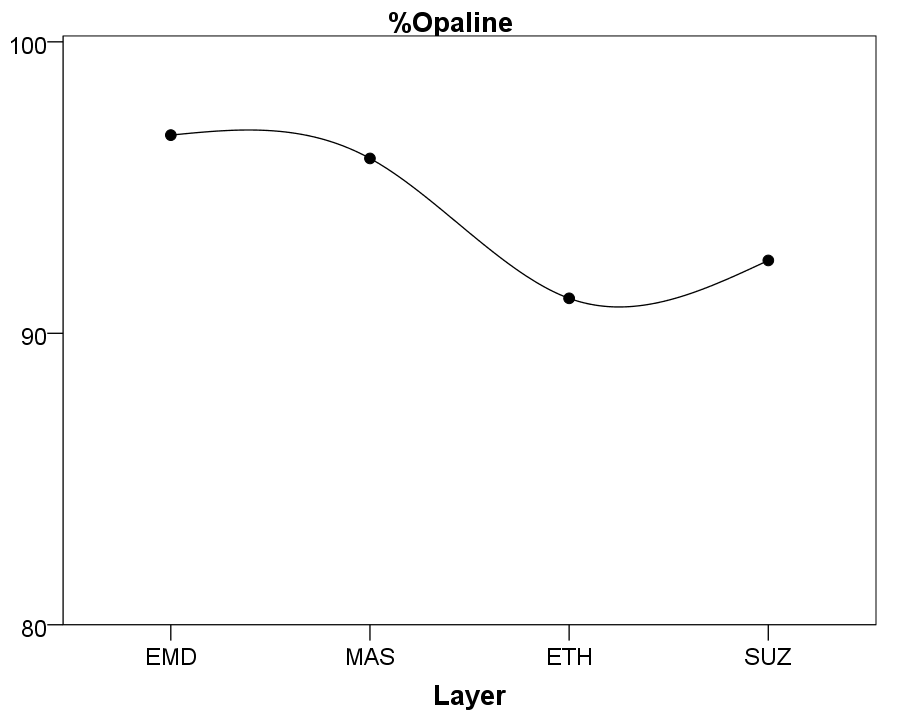


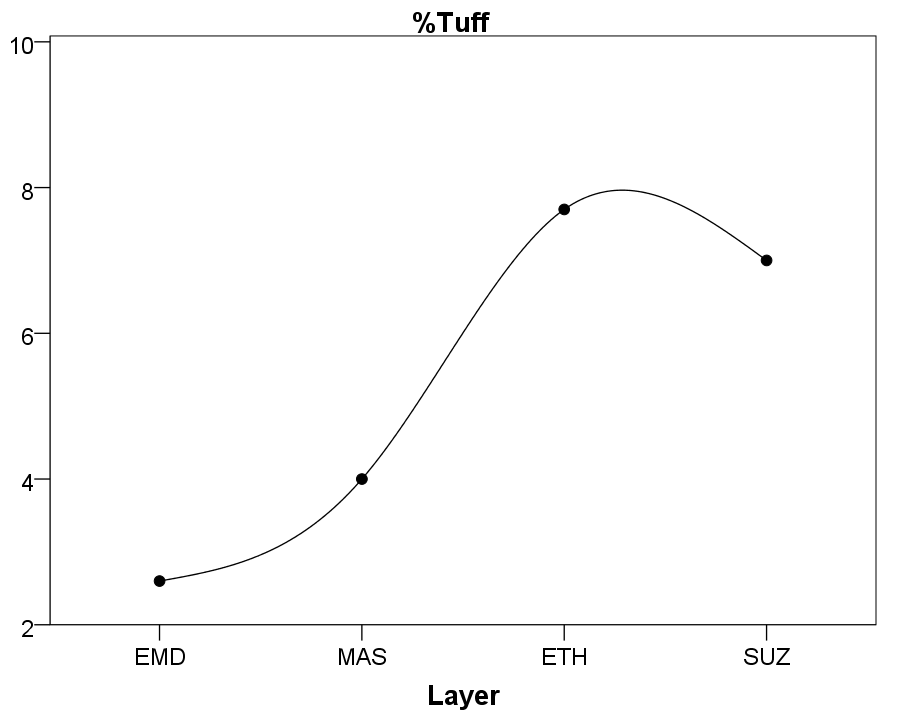


**Layer and %BackedPiece**

- Consistent monotonous decrease throughout the sequence (but data originally pooled for ETH&SUZ)


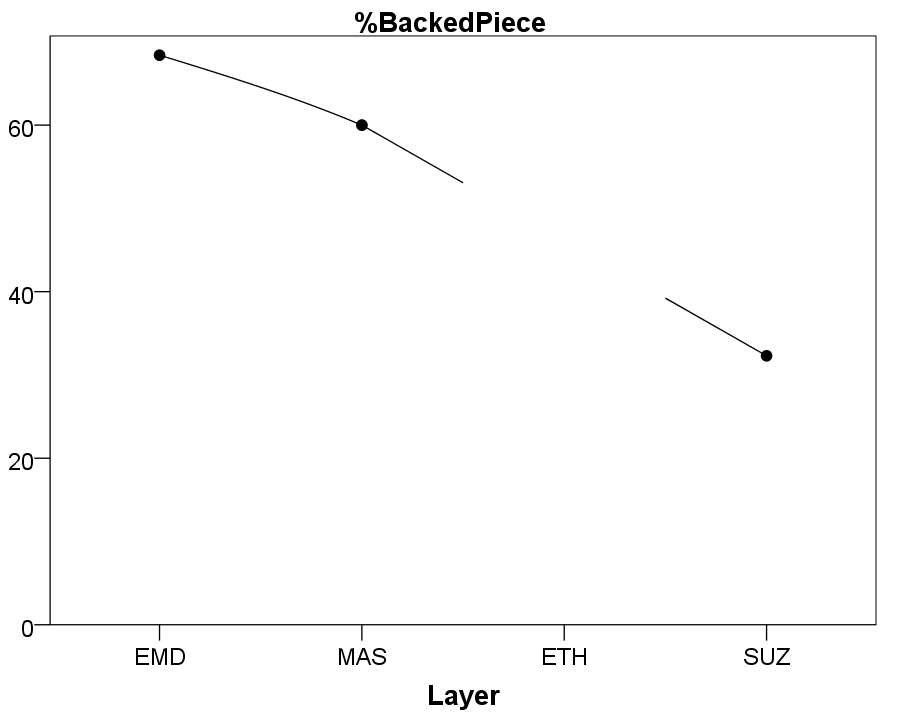


**Layer and %Notched piece**

- No clear unidirectional pattern (rather U-shaped with 1 minimum), but a tendency towards an increase throughout the sequence


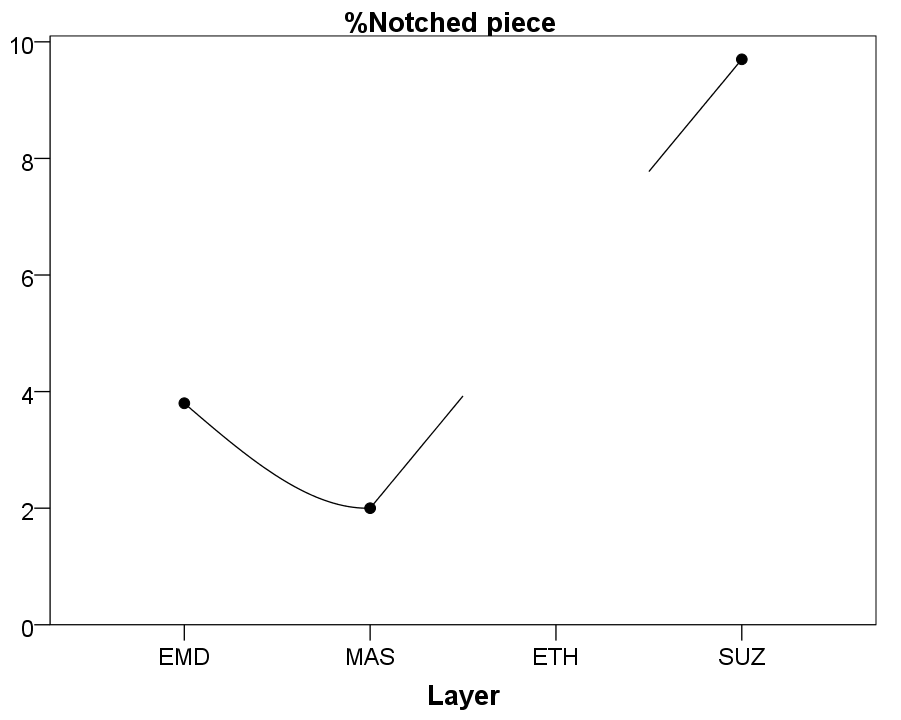


**Layer and %blade**

- Monotonous decrease throughout the sequence


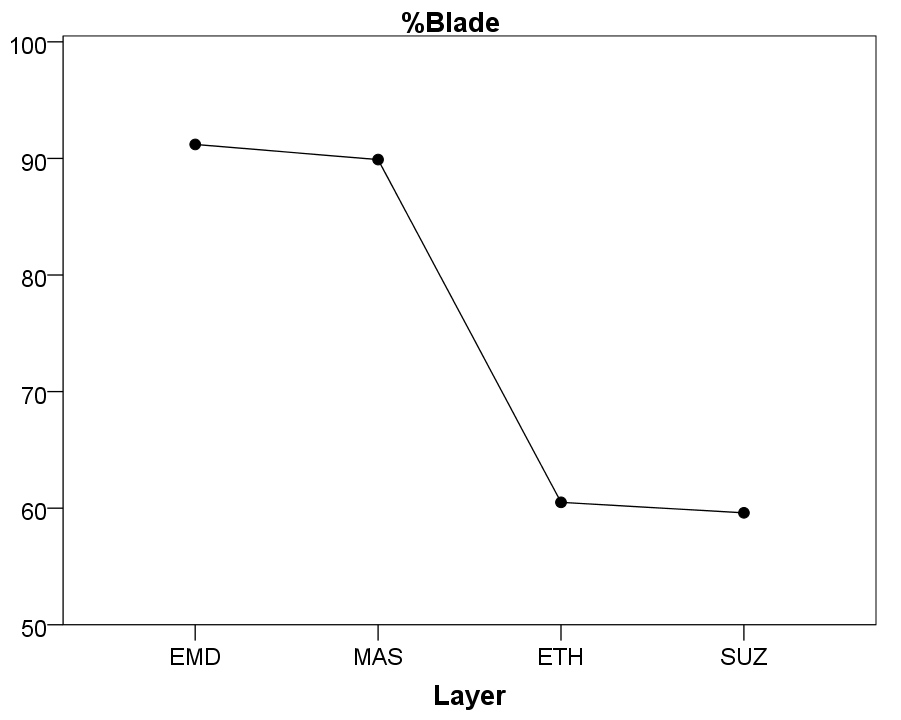


**Layer and %HPandBladeCores**

- Relative consistent unidirectional decrease throughout the sequence


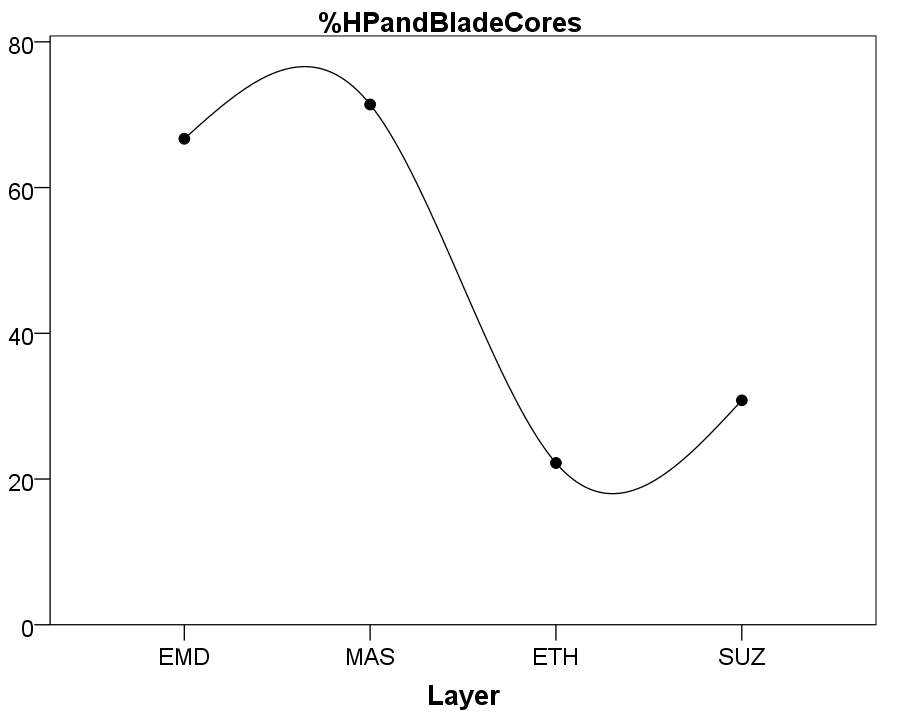


**Layer and % of various knapping traces**

- Consistent unidirectional trends in abrasion and platform<2mm (decrease) and %impactpoints (increase)
- No consistent trends for shattered bulb and lips


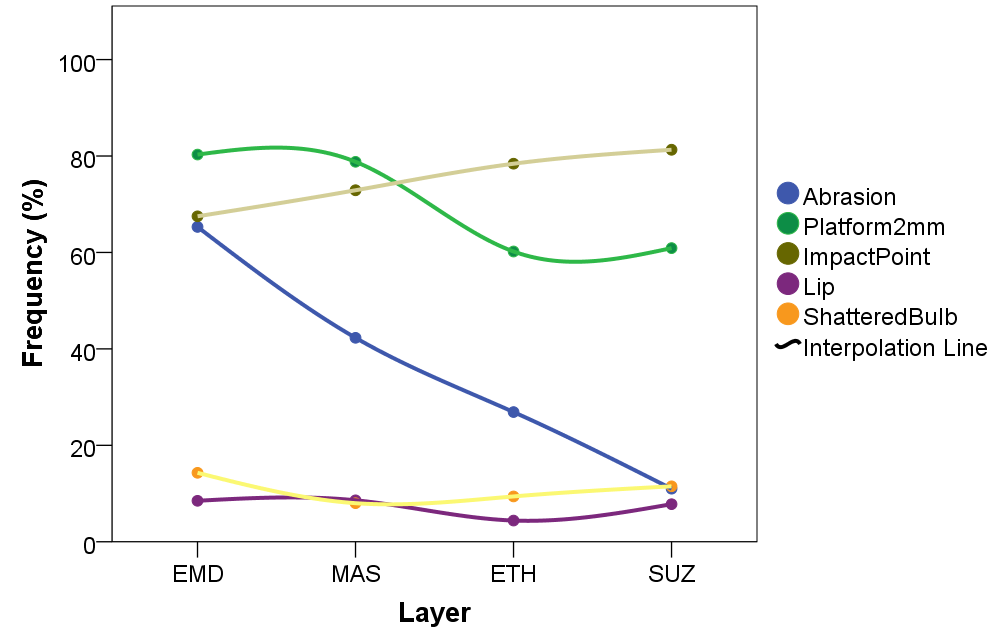


**S8 Text** Summary of statistical analyses for the HP sequence at Umhlatuzana. Raw data on lithic attributes from Kaplan (1990).

**a) Kendall’s tau correlations summary (individual SPSS output below)**

- Unidirectional trends are found **for no variable in a significant manner** but some general directional tendencies visible in the scatter plots.
  - INCREASE in hornfels (but non-significant)
  - DECREASE in quartz (but non-significant)
- No unidirectional trends are found for: %AllBackedPiece, %segments, %trapezoids, Segment/Trapezoid-Ratio, %BladesAndBladelets
  - Characteristic is a **curving pattern** for %AllBackedPieces, %trapezoids, Segment/Trapezoid-Ratio, and %Splintered piece (1peak), BladesAndBladelets (2 peaks)
- Overall, some of the typical HP characteristics at Umhlatuzana decrease when looking only at the uppermost layer 22 (%Quartz, %AllBackedPieces) – comparable to Sibudu and RCC – but not all (e.g. %BladesAndBladelets).
- Also: No significant correlation between %quartz and %backedpiece (t=0.600; p=0.142) and %AllBackedPieces and %BladesAndBladelets (t=0.316; p=0.448)

**Nonparametric Correlations (Layer and %quartz)**

|  | | | | |
| --- | --- | --- | --- | --- |
|  | | | Layer | Quartz |
| Kendall's tau_b | Layer | Correlation Coefficient | 1.000 | -.600 |
|  |  | Sig. (2-tailed) | . | .142 |
|  |  | N | 5 | 5 |
|  | Quartz | Correlation Coefficient | -.600 | 1.000 |
|  |  | Sig. (2-tailed) | .142 | . |
|  |  | N | 5 | 5 |

**Nonparametric Correlations (Layer and %hornfels)**

|  | | | | |
| --- | --- | --- | --- | --- |
|  | | | Layer | Hornfels |
| Kendall's tau_b | Layer | Correlation Coefficient | 1.000 | .600 |
|  |  | Sig. (2-tailed) | . | .142 |
|  |  | N | 5 | 5 |
|  | Hornfels | Correlation Coefficient | .600 | 1.000 |
|  |  | Sig. (2-tailed) | .142 | . |
|  |  | N | 5 | 5 |

**Nonparametric Correlations (Layer and %AllBackedPieces, %Segments, %Trapzeoid, SegmentTrapezoidRatio)**

|  | | | | |
| --- | --- | --- | --- | --- |
|  | | | Layer | BackedPieceAll |
| Kendall's tau_b | Layer | Correlation Coefficient | 1.000 | -.200 |
|  |  | Sig. (2-tailed) | . | .624 |
|  |  | N | 5 | 5 |
|  | BackedPieceAll | Correlation Coefficient | -.200 | 1.000 |
|  |  | Sig. (2-tailed) | .624 | . |
|  |  | N | 5 | 5 |
|  | Segments | Correlation Coefficient | .200 | .600 |
|  |  | Sig. (2-tailed) | .624 | .142 |
|  |  | N | 5 | 5 |
|  | Trapezoid | Correlation Coefficient | -.400 | .800 |
|  |  | Sig. (2-tailed) | .327 | .050 |
|  |  | N | 5 | 5 |
|  | SegmentTrapezoidratio | Correlation Coefficient | .200 | -.600 |
|  |  | Sig. (2-tailed) | .624 | .142 |
|  |  | N | 5 | 5 |

**Nonparametric Correlations (Layer and %SplinteredPiece)**

|  | | | | |
| --- | --- | --- | --- | --- |
|  | | | Layer | SplinteredPiece |
| Kendall's tau_b | Layer | Correlation Coefficient | 1.000 | -.200 |
|  |  | Sig. (2-tailed) | . | .624 |
|  |  | N | 5 | 5 |
|  | SplinteredPiece | Correlation Coefficient | -.200 | 1.000 |
|  |  | Sig. (2-tailed) | .624 | . |
|  |  | N | 5 | 5 |

**Nonparametric Correlations (Layer and %BladeAndBladelets)**

|  | | | Layer | BladesAndBladelets |
| --- | --- | --- | --- | --- |
| Kendall's tau_b | Layer | Correlation Coefficient | 1.000 | -.527 |
|  |  | Sig. (2-tailed) | . | .207 |
|  |  | N | 5 | 5 |
|  | BladesAndBladelets | Correlation Coefficient | -.527 | 1.000 |
|  |  | Sig. (2-tailed) | .207 | . |
|  |  | N | 5 | 5 |

**b) Scatter plots**

**Layer and %Quartz / %Hornfels**

- Both quartz and hornfels show a general directional patters through time, though not monotonously. The trends become particularly apparent in the uppermost 3 layers (Layer 24-22)
- Quartz: decrease throughout the sequence**;** Hornfels: increase throughout the sequence


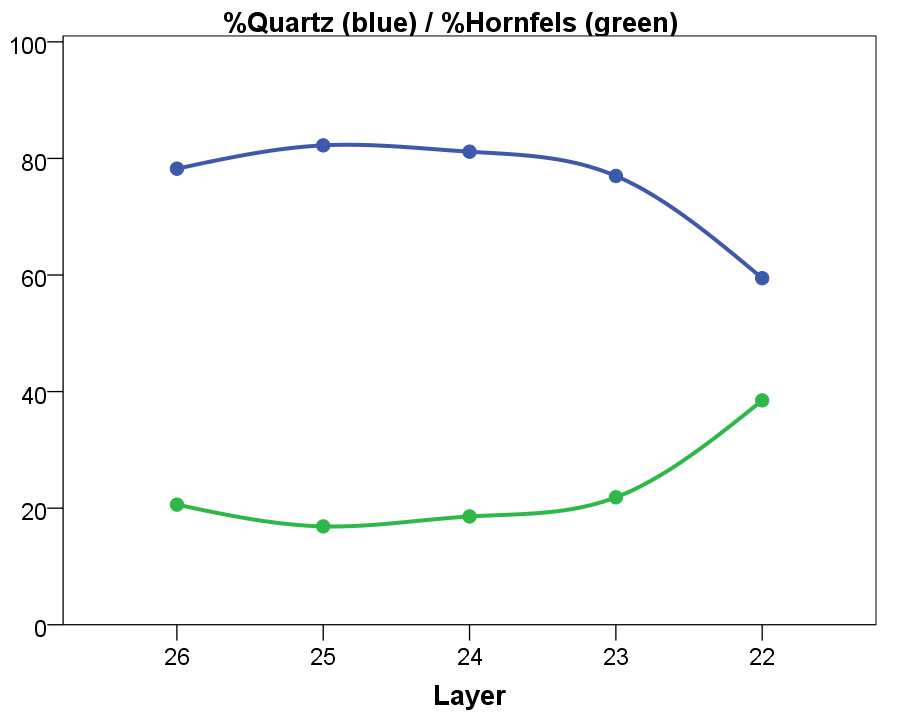


**Layer and %AllBackedPieces / %Segments / %Trapezoid**

- All three variables show non-directional patterns, mostly with one peak
- The middle three layers show a trend towards an increase in segments (Layers 25-23)


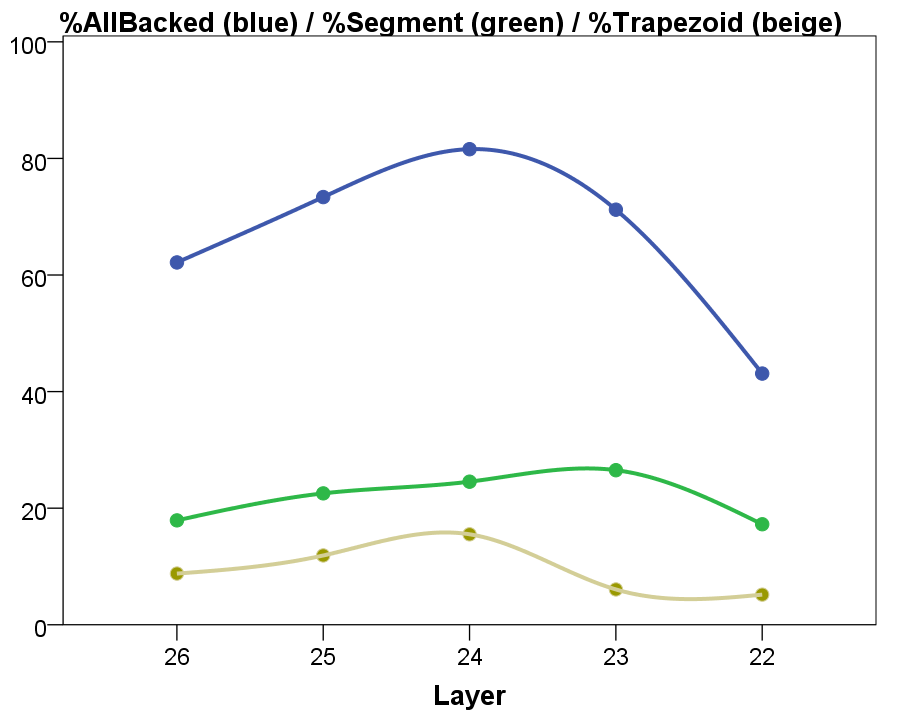


**Layer and Segment/Trapezoid-Ratio**

- Shows a non-directional pattern through time (with 1 peak), with a slight decrease at the end, but still quite high values


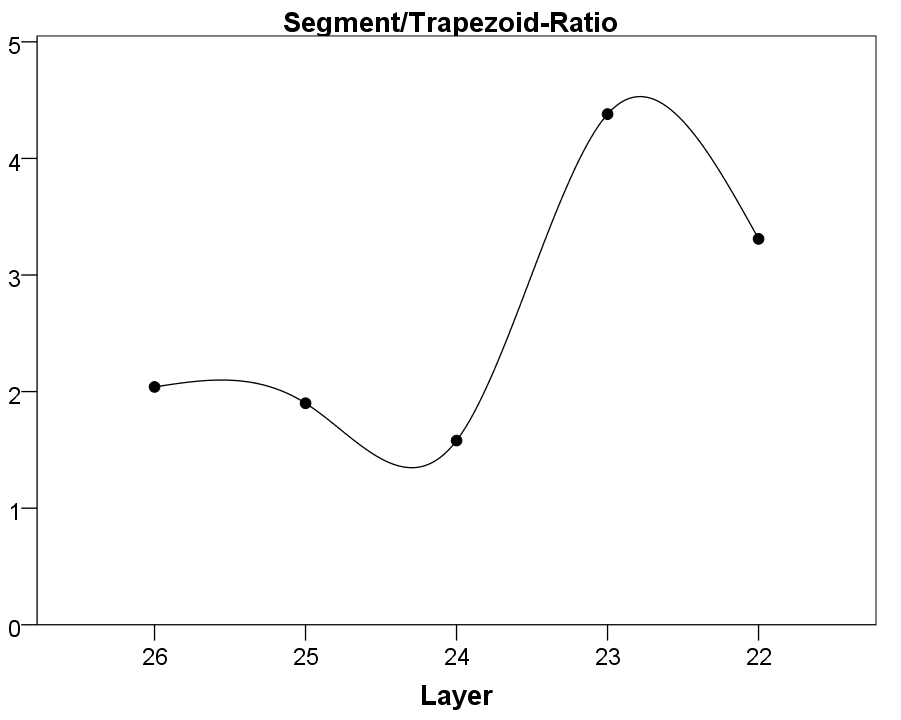


**Layer and %SplinteredPiece**

- Splintered pieces show non-directional pattern, with one peak in the middle of the sequence


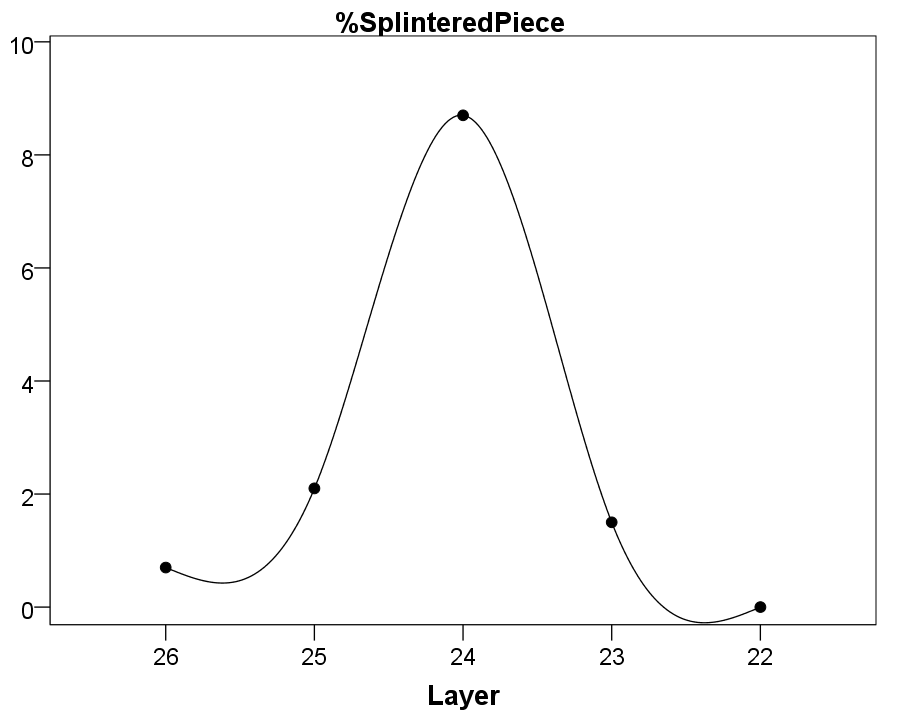


**Layer and %BladeAndBladelets**

- Shows a non-directional curving pattern through time (with 2 peaks), with a slight increase again towards the end of the sequence


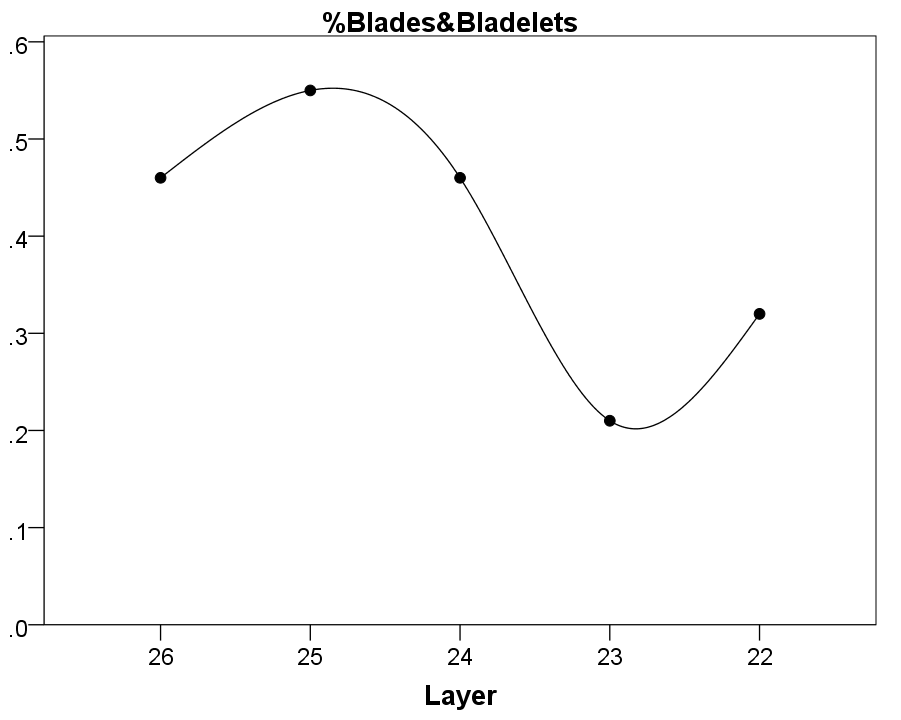


**Additional supplementary references**

- Henshilwood, C. S., Sealy, J. C., Yates, R., Cruz-Uribe, K., et al., 2001. Blombos Cave, southern Cape, South Africa: preliminary report on the 1992–1999 excavations of the Middle Stone Age levels. J. Arch. Sci. 28, 421-448.
- Lombard, M., Phillipson, L., 2010. Indications of bow and stone-tipped arrow use 64 000 years ago in KwaZulu-Natal, South Africa. Antiquity 84, 635–648.
- Marean, C.W., 2010. Pinnacle Point Cave 13B (Western Cape Province, South Africa) in context: the Cape floral kingdom, shellfish, and modern human origins. J. Hum. Evol. 59, 425-443.
- Sharpe, Donald (2015) "Chi-Square Test is Statistically Significant: Now What?," Practical Assessment, Research, and Evaluation: Vol. 20 , Article 8. DOI: https://doi.org/10.7275/tbfa-x148
